# Real-Time Vibrational Spectroscopy Reveals an Inversion Transition State in the Photoisomerization of Phenylazoimidazole

**DOI:** 10.1021/acs.jpclett.6c01358

**Published:** 2026-07-03

**Authors:** Sena Hashimoto, Izumi Iwakura, Tsubasa Tanaka, Akira Takahashi, Atsushi Yabushita, Takayoshi Kobayashi, Atsushi Kameyama

**Affiliations:** † Department of Applied Chemistry, Faculty of Chemistry and Life Science, 12853 Kanagawa University, 3-27-1 Rokkakubashi, Kanagawa-ku, Yokohama 221-8686, Japan; ‡ Department of Chemical Science and Engineering, Institute of Science Tokyo, 2-12-1 Ookayama, Meguro-ku, Tokyo 152-8550, Japan; § Research Institute of Engineering, Kanagawa University, 3-27-1 Rokkakubashi, Kanagawa-ku, Yokohama 221-8686, Japan; ∥ Department of Electrophysics, 34882 National Yang Ming Chiao Tung University, 1001 Ta-Hsueh Road, Hsinchu 300, Taiwan

## Abstract

Whether the productive pathway in azo photoisomerization is N=N rotation or N-inversion remains debated. We use visible 5 fs pump–probe spectroscopy to track the N=N stretching mode (ν_N=N_) during photoisomerization of *trans*-2-(phenylazo)­imidazole (*t*-PAI) after n−π* excitation. Spectrogram analysis reveals an upshift-then-downshift trajectory of ν_N=N_, with an initial upshift from 1450 to 1850 cm^–1^ followed by a downshift. This indicates a transient increase followed by a decrease in N=N bond order, consistent with rehybridization of one azo nitrogen atom toward sp-like character near the quasi-linear inversion geometry. This agrees with TD-DFT and multireference perturbation theory predictions for inversion and is incompatible with those for rotation, which predict the opposite sequence: an initial downshift followed by an upshift toward the *cis*-like geometry. These results provide the first direct vibrational evidence for a transient increase in N=N bond order during azo photoisomerization, supporting the inversion coordinate as the dominant productive pathway.

## Graphical abstract

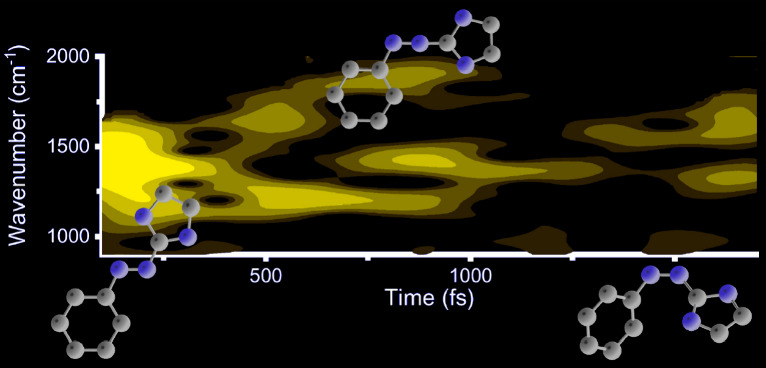

Photoisomerization of azo compounds has long attracted interest owing to their utility as photoswitchable functional materials,^1–10^ yet whether it proceeds via rotation about the azo linkage or inversion at nitrogen remains unresolved (Figure 1).^11,12^ In the rotation pathway,^13–17^ the azo nitrogen atoms rehybridize toward sp^3^-like character^18,19^ as the C–N–N–C dihedral angle (Φ_CNNC_) varies, while the N–N–C bond angle (*A*_NNC_) stays near 120°. In the inversion pathway,^20–27^ one of the azo nitrogen atoms rehybridizes toward sp-like character^28–32^ as *A*_NNC_ opens toward linearity while the other remains near 120°, and the molecular framework stays approximately planar. Several mixed mechanisms^33–43^ have been proposed, including coupled rotation–inversion and concerted pathways, and the prevailing view is that azo photoisomerization involves both coordinates.

**Figure 1:**
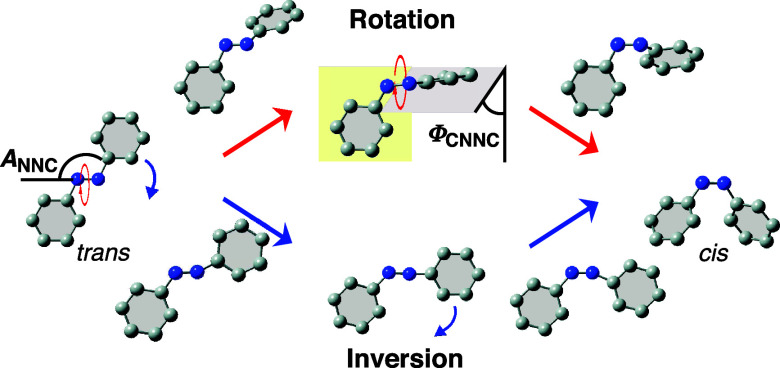
Two candidate pathways for azo photoisomerization. In each model, nitrogen is shown in blue, and carbon in gray. Hydrogen atoms are omitted for clarity.

Direct experimental evidence has remained elusive. Early experimental evidence for inversion was provided by studies of conformationally restricted diazenophanes.^20^ A number of time-resolved vibrational spectroscopic studies have reported that ν_N=N_ in the first excited singlet (S_1_) state of azo compounds shifts only slightly from its ground (S_0_) state value.^23,39–41^ On the theoretical side, however, high-level multireference calculations, such as CASSCF/CASPT2, indicated that the rotation pathway is nearly barrierless via a conical intersection, whereas the inversion pathway has a substantial barrier.^13–16,33^ A key theoretical insight was provided by multireference perturbation calculations,^16^ which showed that the frequency of ν_N=N_ downshifts along the rotation pathway but upshifts by ∼300 cm^–1^ along the inversion pathway, suggesting that the direction of the ν_N=N_ frequency shift can serve as a spectroscopic indicator of the isomerization mechanism; however, no such upshift has been detected experimentally.

We propose that this absence arises from quantum-yield-dependent signal weighting rather than the absence of inversion. In *trans*-azobenzene (*t*-AB), the relatively low photoisomerization quantum yield (Φ_t→c_)^44,45^ results in a dominant nonreactive population whose vibrational response masks that of the reactive subpopulation. To overcome this limitation, we investigated *trans*-2-(phenylazo)­imidazole (*t*-PAI), an arylazoimidazole with higher photoisomerization efficiency than *t*-AB. For example, reported data obtained in comparable low-polarity solvents give Φ_t→c(ππ*)_ = 0.23 for *t*-PAI,^46^ approximately twice that for *t*-AB (0.11).^44,45^ As generally observed for azo compounds, Φ_t→c_ is dependent on excitation wavelength; for *t*-AB, Φ_t→c(nπ*)_ = 0.24, higher than Φ_t→c(ππ*)_ = 0.11.^44,45^ The Φ_t→c(nπ*)_ value of *t*-PAI itself has not been reported, because its relatively rapid thermal *cis*-to-*trans* back-isomerization (τ ≈ 100 s)^46^ results in only a small observable spectral change during initial-rate measurements under continuous irradiation, making reliable determination difficult. In contrast, closely related N-substituted *t*-PAI derivatives undergo thermal back-isomerization approximately 400-fold more slowly than *t*-PAI and exhibit high Φ_t→c(nπ*)_ values of 0.35–0.43.^46,47^ These values support the expectation that *t*-PAI also has substantial n−π* photoisomerization efficiency, thereby enhancing the detectability of reactive vibrational signatures.

Here, by combining visible 5 fs pump–probe measurements^39,48–50^ with spectrogram analysis,^51^ we directly tracked the ν_N=N_ frequency in real time during photoisomerization, revealing a transient upshift that identifies the inversion coordinate as the productive isomerization pathway. Full experimental and computational details are provided in Section S1.

*t*-PAI was prepared according to the previously reported procedure.^52^ The steady-state Raman spectrum of *t*-PAI powder is shown in Section S2. Bands at 1580 and 1460 cm^–1^ are assigned to the aromatic C=C stretching mode (ν_C=C_) and ν_N=N_, respectively, whereas features at 1430, 1395, 1355, 1200, and 1145 cm^–1^ are assigned to CH bending modes (δ_CH_). The band at 990 cm^–1^ is assigned to the benzene ring stretching mode (ν_Ph_). In the S_0_ state, *t*-PAI can adopt *s*-*trans* and *s*-*cis* conformations defined by the orientation of the imidazole C=N bond relative to the central azo unit. Geometry optimization^53–55^ indicates that the *s*-*trans* conformer is lower in energy by ∼3 kcal mol^–1^. Calculated Raman spectra for the two conformers (Figures S1 and S2), after application of a 0.96 scaling factor,^56^ show better agreement with the experimental spectrum for the *s*-*trans* conformer, indicating that *t*-PAI predominantly adopts the *s*-*trans* conformation in the S_0_ state. Therefore, in the following sections, only the calculated results for the *s*-*trans* conformation are presented in the main text, while those for the *s-cis* conformation are provided in Section S3.

Theoretical calculations were performed prior to the ultrafast measurements to predict the evolution of Raman-active vibrational modes along the two candidate photoisomerization pathways (see Section S3 for details). Partial geometry optimizations along the rotation and inversion pathways were carried out at the B3LYP/6-31+G* level^54,55^ by constraining Φ_CNNC_ and *A*_NNC_, respectively. S_1_-state Raman spectra were then calculated for each partially optimized geometry at the TD-B3LYP/6-31+G* level (Figure 2). Similar energy profiles and Raman spectral shifts were obtained when the partial optimizations were performed on the S_1_ surface (Figures S9 and S10).

**Figure 2:**
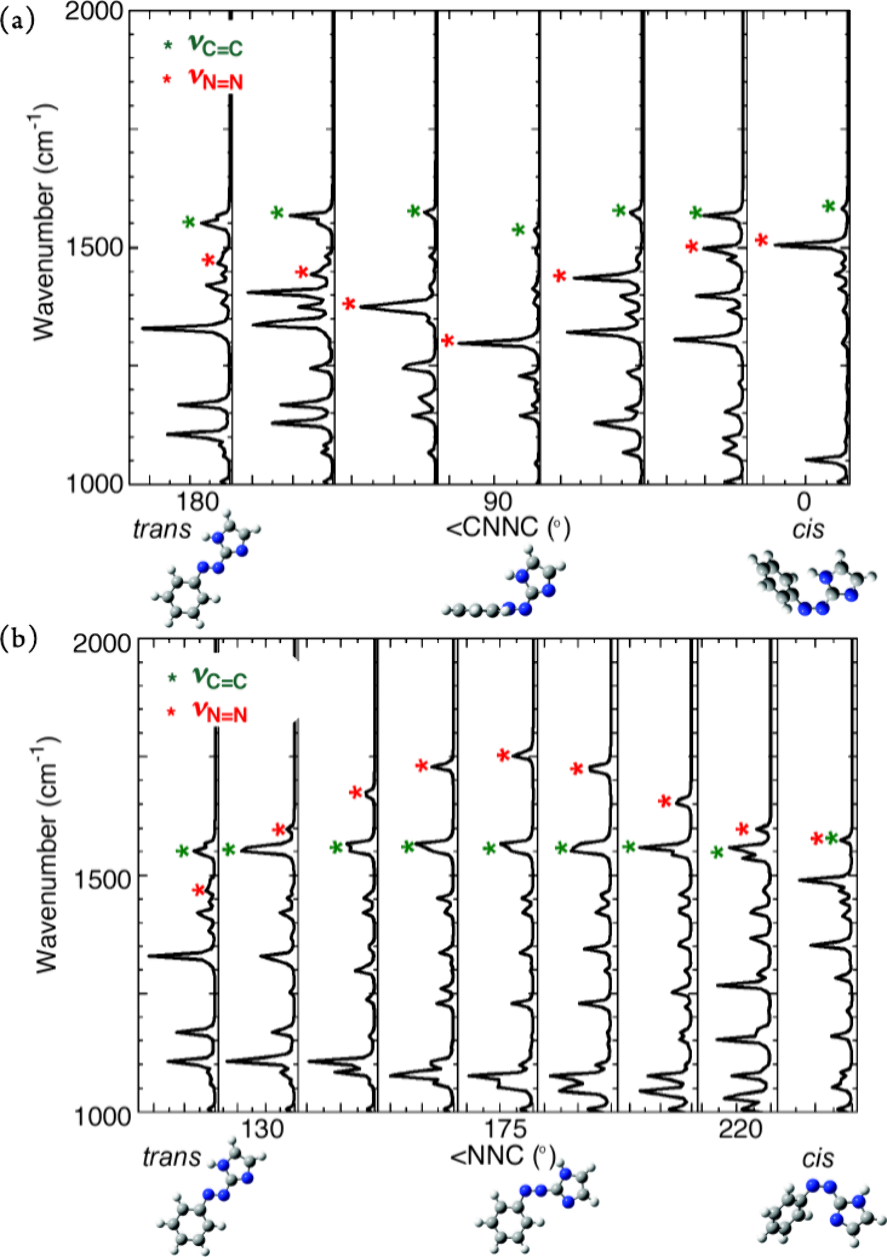
Calculated Raman spectral shifts of *t*-PAI (*s*-*trans* conformer) in the S_1_ state along (a) the rotation pathway and (b) the inversion pathway. Partial geometry optimizations were performed for the S_0_ state.

For *t*-PAI at the Franck–Condon geometry in the S_1_ state (Φ_CNNC_ ≈ 180° and *A*_NNC_ ≈ 115°), the frequency of ν_N=N_ is calculated to be 1470 cm^–1^ and shifts in opposite directions along the two candidate pathways. Along the rotation pathway, ν_N=N_ first downshifts to 1300 cm^–1^ as Φ_CNNC_ approaches 90°, consistent with a decrease in N=N bond order associated with rehybridization of the azo nitrogen atoms toward sp^3^-like character. Subsequently, ν_N=N_ upshifts to 1550 cm^–1^ as twisted PAI proceeds toward the *cis* geometry. Along the inversion pathway, in contrast, ν_N=N_ upshifts to 1750 cm^–1^ as *A*_NNC_ approaches 180°, consistent with an increase in N=N bond order associated with rehybridization of one nitrogen atom toward a quasi-linear sp-like configuration. Thereafter, ν_N=N_ downshifts to 1550 cm^–1^ as quasi-linear PAI proceeds toward the *cis* geometry. By comparison, ν_C=C_ near 1550 cm^–1^ is predicted to remain nearly unchanged. These trends are consistent with the multireference perturbation calculations for *t*-AB.^16^ Thus, the direction of the transient ν_N=N_ frequency shift serves as a practical spectroscopic marker for distinguishing the two pathways, with an initial downshift for rotation and an initial upshift for inversion. Guided by these predictions, visible 5 fs pump–probe measurements were performed to track the real-time frequency evolution of ν_N=N_ following n−π* excitation.

*t*-PAI exhibits a strong π–π* absorption band at 365 nm and a weaker n−π* band near 450 nm (Figure 3a). The spectrum of the generated 5 fs broadband visible pulse (525–725 nm)^57,58^ overlaps with the long-wavelength tail of the n−π* absorption band. A two-dimensional Δ*A* map is shown in Figure 3b, where the horizontal axis, vertical axis, and color scale represent probe wavelength, delay time, and signal intensity, respectively. A positive Δ*A* signal in the longer-wavelength region (550–725 nm) is assigned to induced absorption from the S_1_ state, whereas a negative Δ*A* signal at shorter wavelengths is assigned to stimulated emission. No Δ*A* signal is observed for neat acetonitrile (Section S4), confirming that the observed Δ*A* signal originates from *t*-PAI. In addition, no difference was found between forward- and backward-scan measurements (Section S5), indicating that accumulation of the *cis* isomer (*c*-PAI) does not affect the measurements. The Δ*A* signal amplitude increases linearly with pump pulse energy (Figure S17), confirming that the observed Δ*A* signals predominantly originate from one-photon excitation.

**Figure 3:**
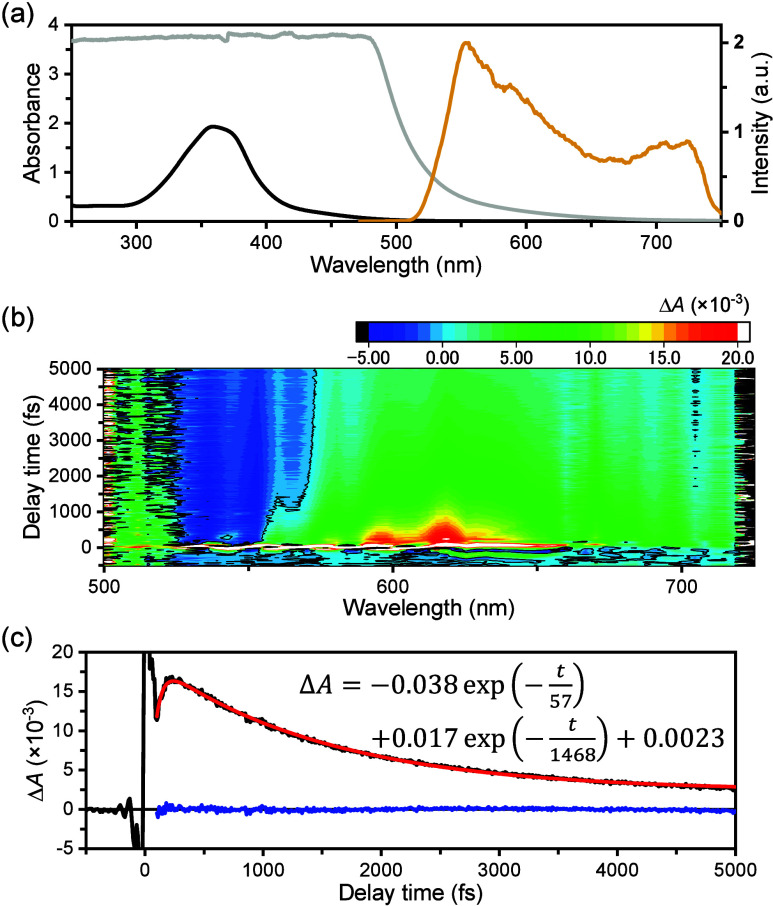
Ultrafast pump–probe measurements of *t*-PAI in acetonitrile. (a) Steady-state absorption spectra measured with a 1 mm optical path length at concentrations of 1.0 × 10^–3^ mol L^–1^ (black) and 8.0 × 10^–2^ mol L^–1^ (gray). The latter sample was used for the pump–probe measurements. The spectrum of the visible 5 fs pulse is also shown (orange). (b) Two-dimensional Δ*A* map (forward scan). (c) Δ*A* trace at 620 nm (black) and its high-frequency (oscillatory) component (blue) obtained by subtracting the biexponential fit (red).

Figure 3c shows the Δ*A* trace probed at 620 nm, where the Δ*A* signal reaches its maximum in Figure 3b; this wavelength is suitable for oscillatory analysis. The trace was fitted with a biexponential function with a constant offset according to eq 1:

1 $$\Delta A\left(\lambda ,t\right)=DAS_{1}\left(\lambda \right)\;\exp\left(-t/\tau_{1}\right)+DAS_{2}\left(\lambda \right)\;\exp\left(-t/\tau_{2}\right)+DAS_{\infty }\left(\lambda \right)$$

where *DAS*_i_(λ) denotes the decay-associated spectrum of each component, yielding lifetimes τ_1_ = 57 fs and τ_2_ = 1.5 ps. These time constants are consistent with those reported for azobenzene derivatives,^23,24^ which typically undergo subpicosecond relaxation from the Franck–Condon region followed by picosecond-scale photoisomerization.

The oscillatory component of Δ*A*, reflecting molecular vibrations, was extracted by subtracting the biexponential fit from the Δ*A* trace. The extracted oscillatory component is shown as the blue curve in Figure 3c, while the biexponential fit is shown as the red curve. A short-time Fourier transform of this periodic modulation (spectrogram analysis) was then applied to obtain the time-dependent instantaneous vibrational frequency, enabling direct visualization of structural evolution from the femtosecond to the picosecond time regime. A greatly enlarged view of the oscillatory component is provided in Figure S16a.

Based on the theoretical prediction that ν_N=N_ initially downshifts along rotation but initially upshifts along inversion, we next examined the experimental spectrograms. Figure 4 shows the time–frequency spectrogram obtained using a Blackman window function with a full width at half-maximum of 320 fs. The horizontal axis represents the pump–probe delay, whereas the vertical axis and color scale represent the instantaneous vibrational frequency and signal intensity, respectively. As a reference, the spectrogram of neat acetonitrile shows a band near 2200 cm^–1^ assigned to ν_C≡N_ (Figure S13). The ν_C≡N_ band shows no detectable frequency shift, consistent with the absence of any photochemical response of the solvent under visible 5 fs excitation.

**Figure 4:**
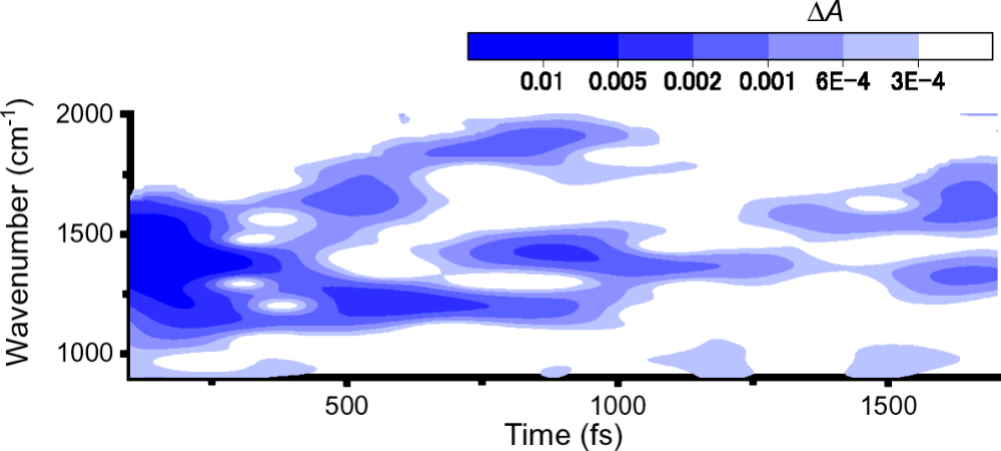
Spectrogram of the acetonitrile solution of *t*-PAI probed at 620 nm (forward scan).

In contrast, the spectrogram of *t*-PAI (Figure 4) immediately after photoexcitation shows distinct bands at 1550, 1450, and 1200 cm^–1^, assigned to ν_C=C_, ν_N=N_ and δ_CH_, respectively. The ν_C=C_ band remains nearly constant in frequency, consistent with the calculations, whereas the ν_N=N_ band, initially observed near 1450 cm^–1^, undergoes a pronounced upshift to 1850 cm^–1^ within the first 1 ps. It then gradually downshifts toward 1500 cm^–1^ over 1.0–1.5 ps. The transient initial upshift of approximately 400 cm^–1^ is fully consistent with the inversion pathway and incompatible with the rotation pathway, which predicts an initial downshift.

The observed spectral evolution can be mapped onto the calculated structural pathway on the S_1_ surface. The initial ultrafast component (τ_1_ ≈ 57 fs) is assigned to structural relaxation following n−π* excitation, accompanied by shortening of the C–N bond linking the aromatic ring to the azo nitrogen and opening of *A*_NNC_ from 115° to 130°. During this relaxation, the excited population is expected to branch into a reactive fraction that proceeds along the inversion coordinate and a nonreactive fraction that returns to the trans S_0_ state; the high intrinsic photoisomerization efficiency of *t*-PAI is expected to provide a substantial reactive fraction.

The subsequent ν_N=N_ evolution occurs on the 1.5 ps time scale of τ_2_: the continuous upshift observed up to 1 ps indicates motion along the inversion coordinate as *A*_NNC_ increases from 130° to the quasi-linear region near 180°, whereas the following downshift over 1.0–1.5 ps is assigned to further motion beyond the quasi-linear region toward the cis-like geometry. The low barrier calculated along the optimized inversion pathway of *t*-PAI (Figure S9) is consistent with this motion occurring on the picosecond time scale. Because the maximum frequency of 1850 cm^–1^ corresponds to the transient quasi-linear configuration near the top of this barrier, through which the wavepacket passes only briefly, this feature appears within a narrow time window of 0.6–1.0 ps rather than persisting over the full τ_2_. Its limited appearance thus directly reflects transient passage through the barrier along the inversion coordinate. This complete upshift-then-downshift pattern reproduces the calculated ν_N=N_ profile along the inversion coordinate (Figure 2b) and is incompatible with the rotation pathway, for which an initial downshift followed by an upshift toward the *c*-PAI frequency is predicted.

Notably, this pronounced structural evolution is accompanied by only a minor change in the induced-absorption band. The 620 nm probe monitors S_1_-state absorption over the range of structures sampled along the inversion coordinate, because the calculated S_1_–S_2_ energy gap changes only modestly along this coordinate (Figure S9). Thus, the structural evolution is manifested not as a pronounced shift of the electronic absorption band, but rather as a time-dependent shift of ν_N=N_. Therefore, the nearly unchanged induced-absorption band does not indicate that the molecular structure remains static.

To clarify why no ν_N=N_ upshift has been reported for *t*-AB, we performed 5 fs pump–probe measurements on *t*-AB under the same conditions (Section S6 for details). The transient dynamics (τ_1_ ≈ 150 fs, τ_2_ ≈ 1.2 ps) agree with previous reports,^23,42^ and no significant ν_N=N_ upshift is observed. Instead, only a minor shift of the ν_C–N_ band is detected, consistent with TD-DFT predictions for relaxation from the Franck–Condon region to the S_1_ minimum.

This contrast can be rationalized by the difference in photoisomerization quantum yields. In *t*-AB, most molecules relax back to the S_0_ state without isomerization. The measured signal therefore reflects a superposition of reactive and nonreactive populations, with the latter dominating and masking the ν_N=N_ upshift associated with inversion. In contrast, the higher quantum yield of *t*-PAI increases the contribution of the reactive population, thereby enabling direct observation of the ν_N=N_ upshift. Thus, the absence of this signature in *t*-AB does not exclude the inversion pathway but reflects limited sensitivity to the minority reactive fraction.

In conclusion, using ultrafast vibrational spectroscopy with visible 5 fs pulses, we directly observed the real-time evolution of ν_N=N_ during photoisomerization of *t*-PAI following n−π* excitation. For *t*-PAI, ν_N=N_, initially observed near 1450 cm^–1^, upshifts to 1850 cm^–1^ within the first 1 ps and then gradually downshifts as the reaction proceeds. Along the inversion pathway, this upshift-then-downshift temporal pattern is consistent with a transient increase followed by a decrease in N=N bond order as one azo nitrogen atom approaches sp-like character, as predicted by theoretical calculations. Along the rotation pathway, in contrast, ν_N=N_ is expected to show the opposite sequence: an initial downshift, reflecting a decrease in N=N bond order induced by rehybridization of the azo nitrogen atoms toward sp^3^-like character, followed by an upshift toward the *cis*-like geometry.

These results provide the first direct vibrational evidence for a transient increase in N=N bond order during azo photoisomerization and indicate that the inversion coordinate is the dominant productive reaction pathway. More broadly, our findings indicate that inversion may play a more important role in azo photoisomerization than previously recognized, particularly under n−π* excitation conditions.

## Supplementary Material



## Notes

Experimental details; Raman spectra in the S_0_ state; calculated Raman spectral shifts of t-PAI in the S_1_ state and energy profiles; pump–probe measurements of neat acetonitrile using a visible 5 fs pulse laser; pump–probe measurements of t-PAI and t-AB using a visible 5 fs pulse laser; optimized structures (PDF)
