# Supplementary material for: Real-Time Vibrational Spectroscopy Reveals an Inversion Transition State in the Photoisomerization of Phenylazoimidazole
Source: J Phys Chem Lett. 2026 Jul 3;17(28):7945–50. doi: 10.1021/acs.jpclett.6c01358 (PMC13383832; doi:10.1021/acs.jpclett.6c01358)
Supplement: Supplementary file 1 [file jz6c01358_si_001.pdf]

Supporting Information

Real-Time Vibrational Spectroscopy Reveals an Inversion Transition State in the Photoisomerization of Phenylazoimidazole

Sena Hashimoto[a], Izumi Iwakura[a,*], Tsubasa Tanaka[a], Akira Takahashi[b], Atsushi Yabushita[c,d], Takayoshi Kobayashi[c,d], and Atsushi Kameyama[a,*]

[a]Department of Applied Chemistry, Faculty of Chemistry and Life Science, Kanagawa University, 3-27-1 Rokkakubashi, Kanagawa-ku, Yokohama 221-8686, Japan

[b]Department of Chemical Science and Engineering, Institute of Science Tokyo, 2-12-1 Ookayama, Meguro-ku, Tokyo 152-8550, Japan

[c]Research Institute of Engineering, Kanagawa University, 3-27-1 Rokkakubashi, Kanagawa-ku, Yokohama 221-8686, Japan

[d]Department of Electrophysics, National Yang Ming Chiao Tung University, 1001 Ta-Hsueh Road, Hsinchu 300, Taiwan

Email: izumi@kanagawa-u.ac.jp, kameya01@kanagawa-u.ac.jp

Contents

Section S1. Experimental Section S2

Section S2. Calculated and Measured Raman Spectra in S_0-State S3

Section S3. Calculated Raman Spectral Shifts of *t*-PAI in S_1-State S7

Section S4. Pump–Probe Measurement of Neat Acetonitrile using Visible 5-fs Pulse Laser S13

Section S5. Pump–Probe Measurement of *t*-PAI using Visible 5-fs Pulse Laser S15

Section S6. Pump–Probe Measurement of *t*-AB using Visible 5-fs Pulse Laser S17

Section S7. Optimized Structure S21

Section S1. Experimental Section

S1.1. Steady-State Spectroscopy

Steady-state Raman, absorption, and fluorescence spectra were measured using commercial spectrometers: V-650 (JASCO) for absorption, FP-6600 (JASCO) for fluorescence, and NRS-5500 (JASCO) for Raman measurements.

S1.2. Theoretical Calculations

All quantum chemical calculations were performed using the Gaussian 16 program package[53] without imposing symmetry constraints. Geometry optimization and calculation of Raman-active molecular vibrational frequencies were performed at the B3LYP/6–31+G(d) level[54,55] for Ground (S_0) state and at the TD-B3LYP/6–31+G(d) level for the singlet electronic excited (S_n) states.

S1.3. Transient-State Spectroscopy with Temporal Resolution of Molecular Vibration

A Ti:sapphire regenerative amplifier (SpectraPhysics, Spitfire model) was used to generate near-infrared (NIR) femtosecond pulses (central wavelength: 800 nm, pulse duration: 100 fs, repetition rate: 1 kHz, pulse energy: 3 mJ). These pulses were converted into ultrashort broadband visible pulses (500–740 nm) using a home-built noncollinear optical parametric amplifier. Details of the optical setup are described elsewhere.[57,58]

The amplified broadband visible pulse was split into pump and probe pulses with an intensity ratio of 10:1. The chirp of both pulses was compensated to achieve sub-10 fs pulse durations at the sample position, enabling temporal resolution of molecular vibrations. The time-resolved absorption change of the probe pulse was measured by modulating the pump pulse with a mechanical chopper at 500 Hz. A fast-scan-rate CCD line-scan camera (Entwicklungsbüro Stresing, Series 2000) was used to detect probe pulses on a shot-to-shot basis, allowing differential absorption signals to be obtained from consecutive pump-on and pump-off measurements. The time delay between pump and probe pulses was scanned from –100 to 5000 fs with a step size of 0.2 fs. For signal-to-noise improvement, every five consecutive delay points were averaged during data processing. Because the pulse duration (~5 fs) is shorter than the vibrational period of relevant molecular vibrations (e.g., ~23 fs for $\nu_{N=N}$ at ~1460 cm^{-1}), the oscillatory wavepacket motion of individual vibrational modes can be temporally resolved as a sinusoidal modulation of the transient absorption signal[48-50]. Application of a short-time Fourier transform (spectrogram analysis)[51] to this modulation yields the instantaneous vibrational frequency as a function of reaction time, thereby enabling direct visualization of transient structural changes along the reaction coordinate.

S1.4. Sample

t-PAI was prepared according to the previously reported procedure.[52] For steady-state absorption and 5-fs pump–probe measurements, *t*-PAI was dissolved in acetonitrile at concentrations of $1.0\text{x}10^{-3}$ mol L^{-1} and $8.0\text{x}10^{-2}$ mol L^{-1}, respectively. In both measurements, the solution was placed in a fused-silica glass cell (GL Sciences Inc., S15–IR–1) with an internal optical path length of 1 mm. For comparison, *t*-AB solutions in acetonitrile at concentrations of 1.0×10^{-3} and 1.0 mol L^{-3} were measured under otherwise identical conditions. Each sample solution was fixed on an XY-motorized stage which continuously moves in an octagonal path in a plane orthogonal to the optical path. This continuous scanning during the pump–probe measurement excludes the accumulation of the *cis* isomer produced by the photoisomerization under study.

Section S2. Calculated and Measured Raman Spectra in S_0-State

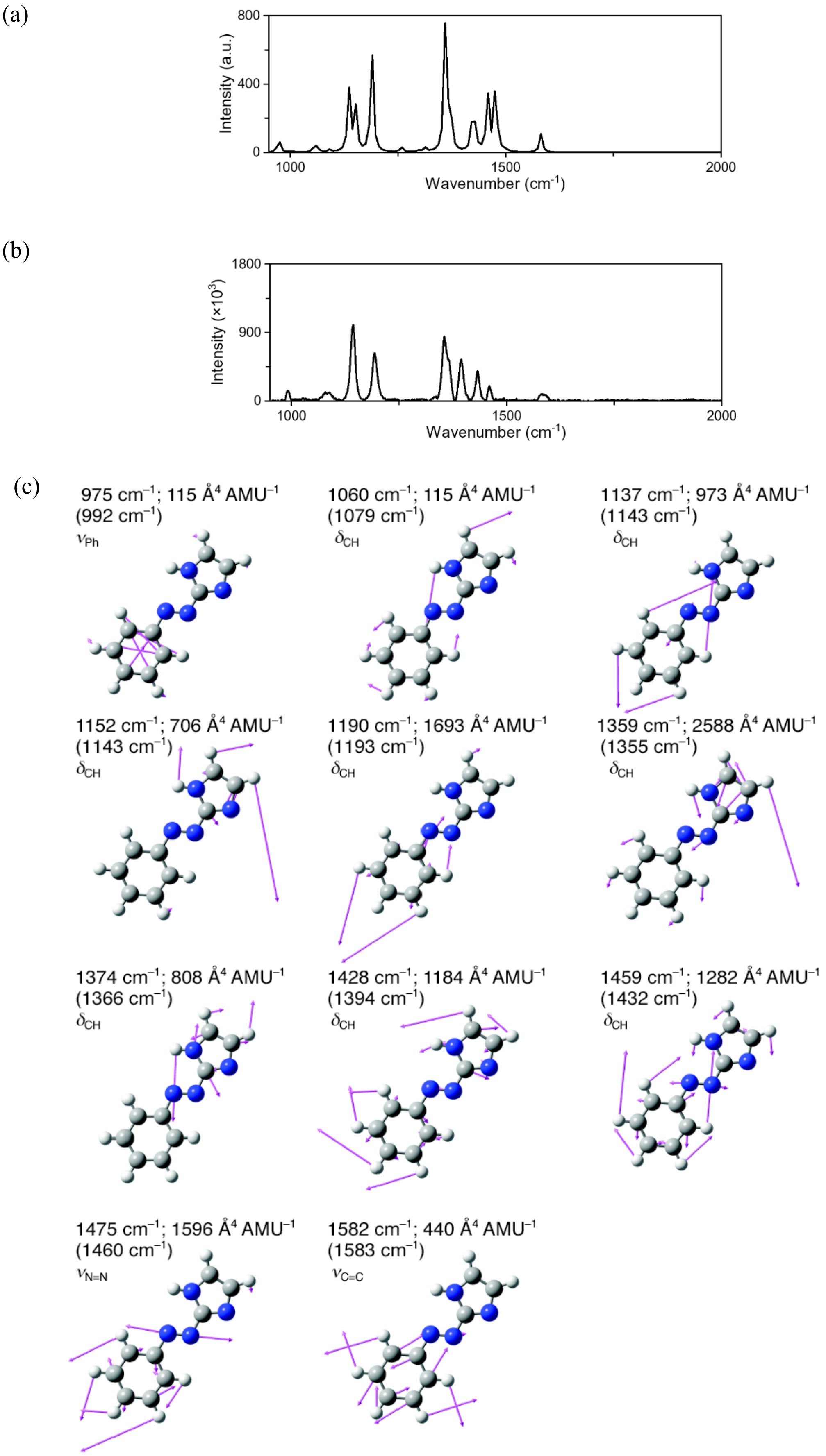

Figure S1. (a) Raman spectra of calculated result of *t*-PAI (*s-trans* conformer) and (b) measured result of *t*-PAI powder (c) Raman active vibrational modes (Values are harmonic frequencies and Raman scattering activities from theoretical calculation. Values within parentheses are the molecular vibrational frequencies resolved from the experiment).

(a)

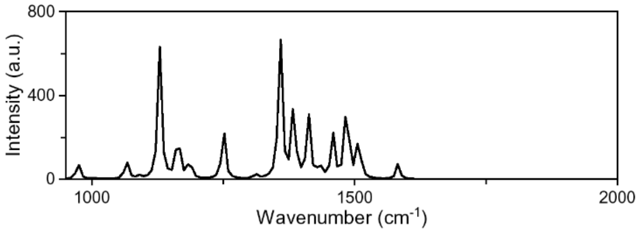

(b)

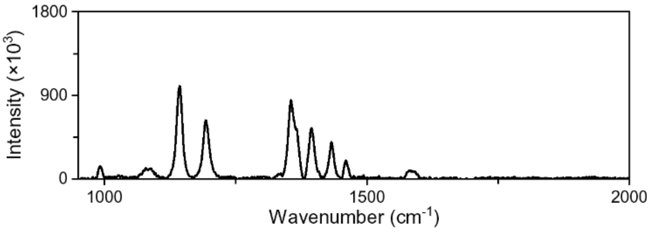

(c)

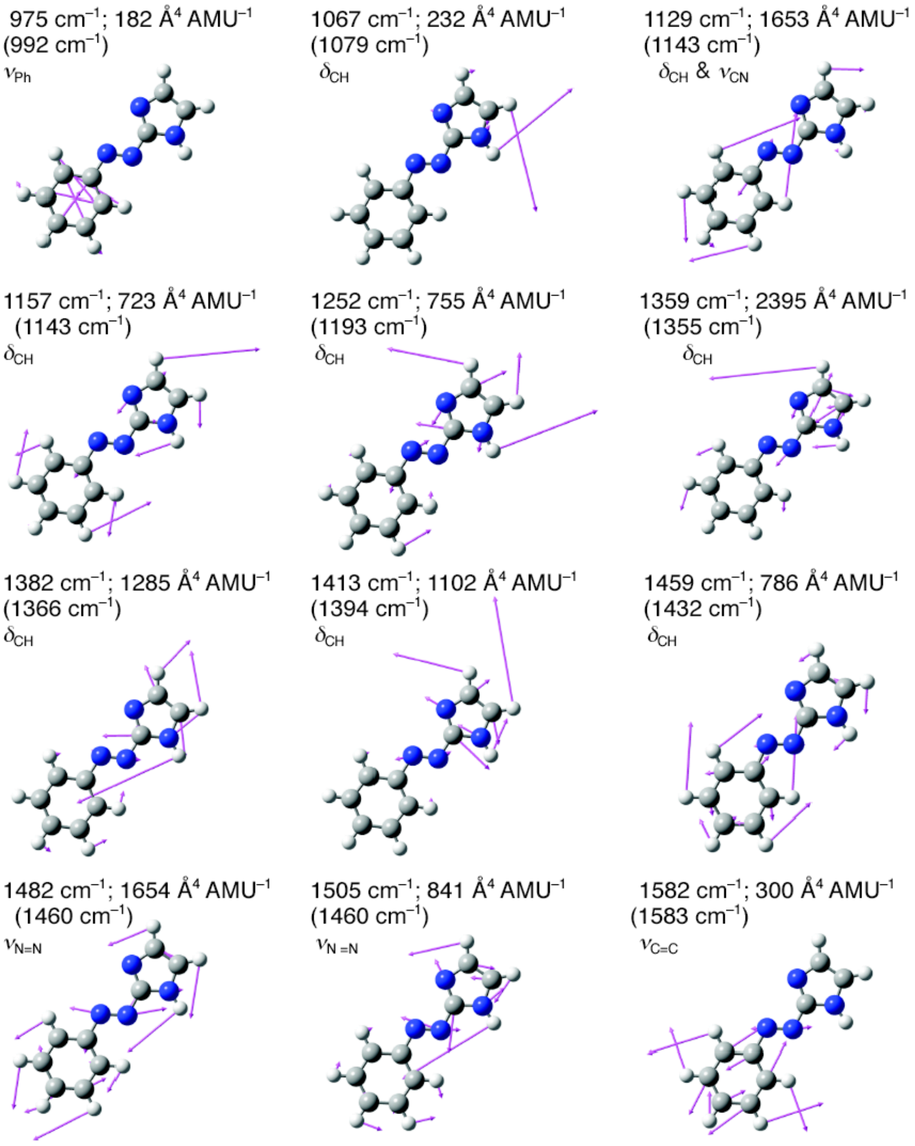

Figure S2. (a) Raman spectra of calculated result of *t*-PAI (*s-cis* conformer) and (b) measured result of *t*-PAI powder (c) Raman active vibrational modes (Values are harmonic frequencies and Raman scattering activities from theoretical calculation. Values within parentheses are the molecular vibrational frequencies resolved from the experiment).

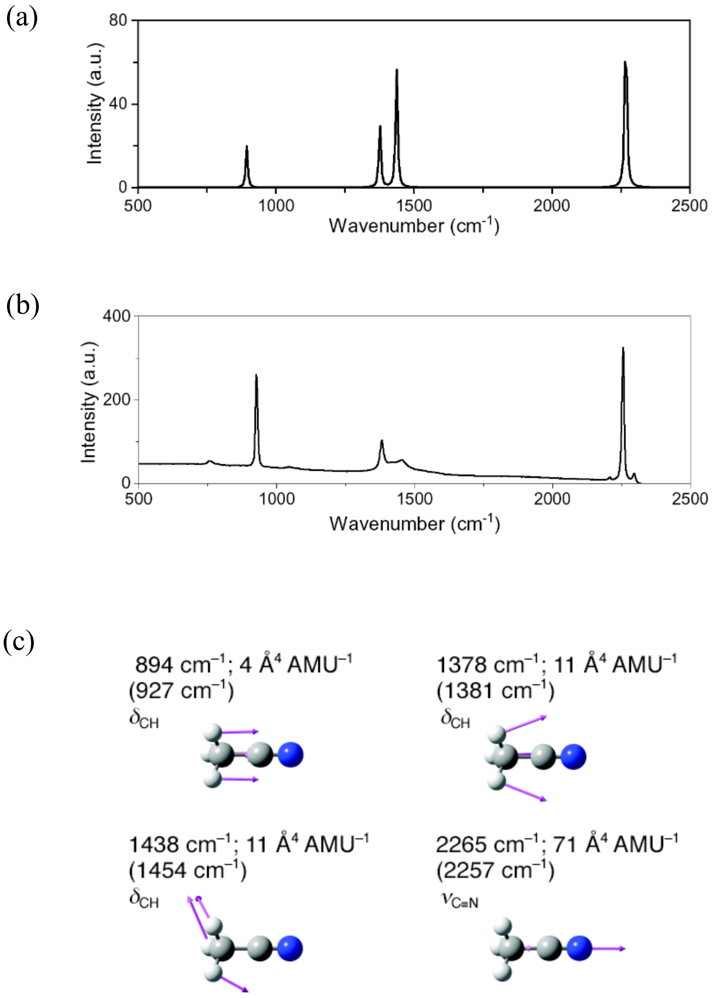

Figure S3. (a) Raman spectra of calculated result and (b) measured result of acetonitrile. (c) Raman active vibrational modes (Values are harmonic frequencies and Raman scattering activities from theoretical calculation. Values within parentheses are the molecular vibrational frequencies resolved from the experiment).

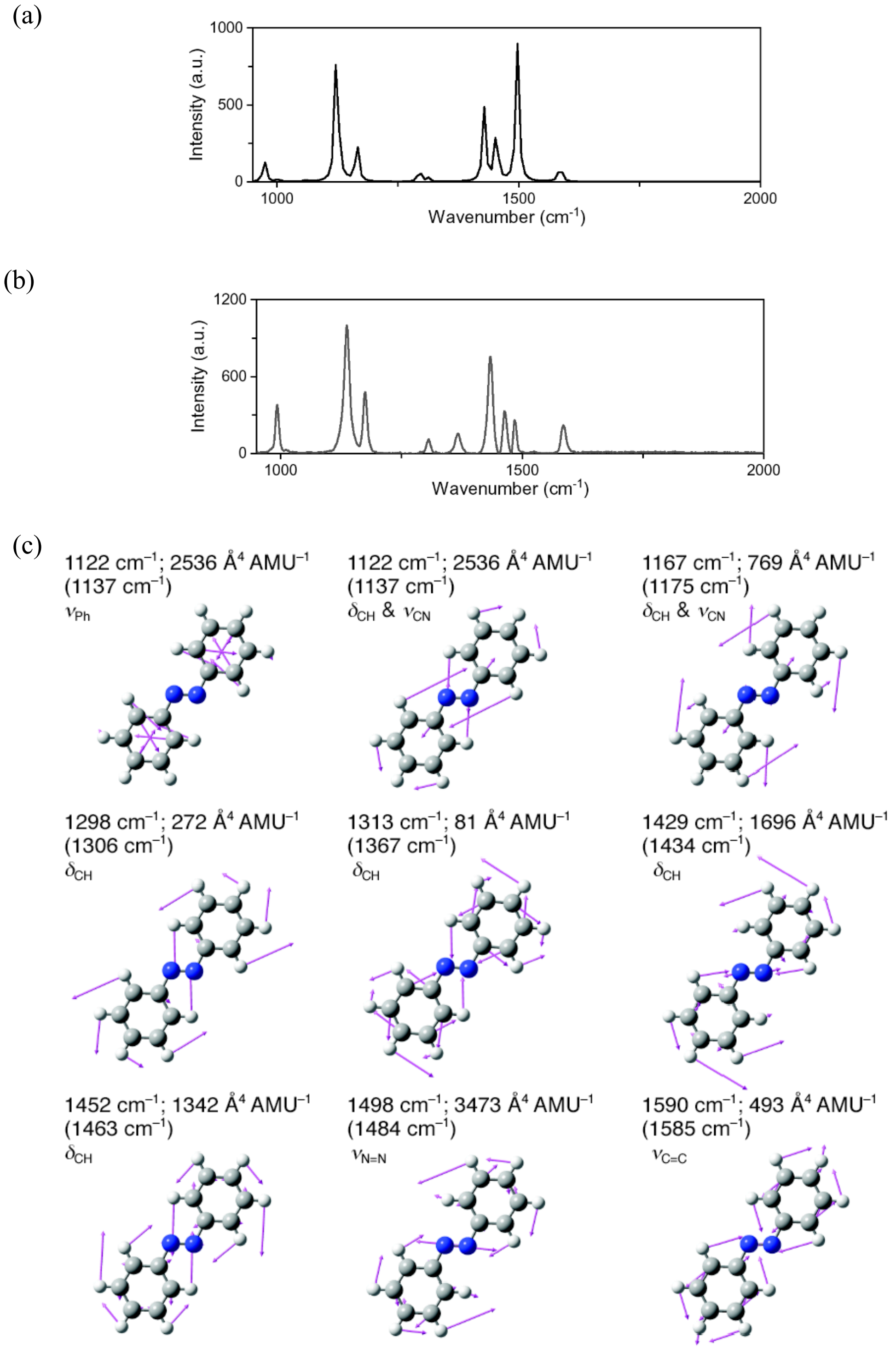

Figure S4. (a) Raman spectra of calculated result of *t*-AB and (b) measured result of *t*-AB powder. (c) Raman active vibrational modes (Values are harmonic frequencies and Raman scattering activities from theoretical calculation. Values within parentheses are the molecular vibrational frequencies resolved from the experiment).

Section S3. Calculated Raman Spectral Shifts of *t*-PAI in S_1-State

For rotation pathway, partial optimization fixed in value of Φ_{CNNC} was performed in S_0-state, with changing Φ_{CNNC} from 0 to 180° with about 30° step. For each calculated partially optimized structure, the energies of S_1-state and S_2-state were calculated by single point calculation. The calculated energy profile of the rotation pathway is shown in Figure S5 where bold and thin lines represent the reaction from *s-trans* and *s-cis* conformers, respectively. The results are nearly comparable with the reported data of *t*-AB.[15,36] Thus, the structures obtained by partial optimization (Section S7; PAI-S0-R150 to PAI-S0-R30) were used for the frequency calculations.

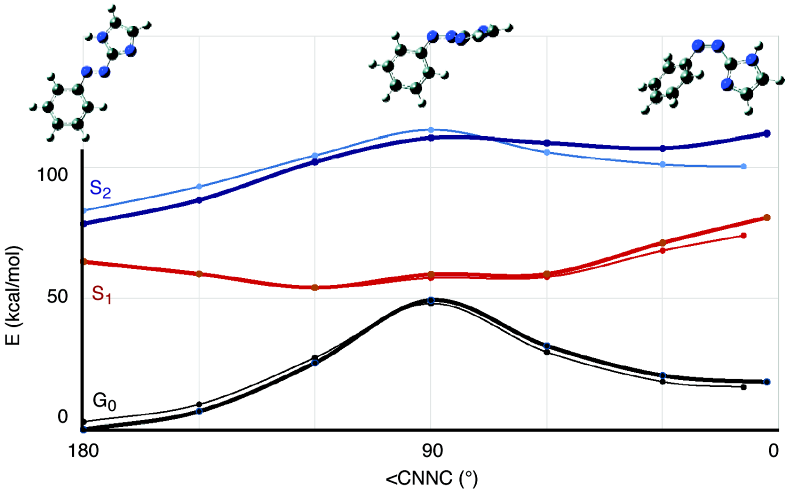

Figure S5. Calculated energy profile of the rotation pathway. Partially optimizations were performed in S_0-state. Reactant (*t*-PAI) was *s-trans* conformer (bold line) and *s-cis* conformer (thin line).

The calculation results (Figure S6) show that, regardless of whether the *s-trans* or *s-cis* conformers, under the rotational pathway, first $\nu_{N=N}$ downshifts, then upshifts. Partially optimized structures of *s-trans* and *s-cis* conformers (PAI-S0-R150 ~ PAI-S0-R30) are shown in Section S7. When the reactant was *s-trans* conformer, $\nu_{N=N}$ downshifts from 1470 cm^{-1} (when Φ_{CNNC}=180°) to 1300 cm^{-1} (when Φ_{CNNC}=90°), then upshifts to 1505 cm^{-1} (when Φ_{CNNC}=0°). Other hand, when the reactant was *s-cis* conformer, $\nu_{N=N}$ downshifts from 1490 cm^{-1} (when Φ_{CNNC}=180°) to 1400 cm^{-1} (when Φ_{CNNC}=90°), then upshifts to 1575 cm^{-1} (when Φ_{CNNC}=0°).

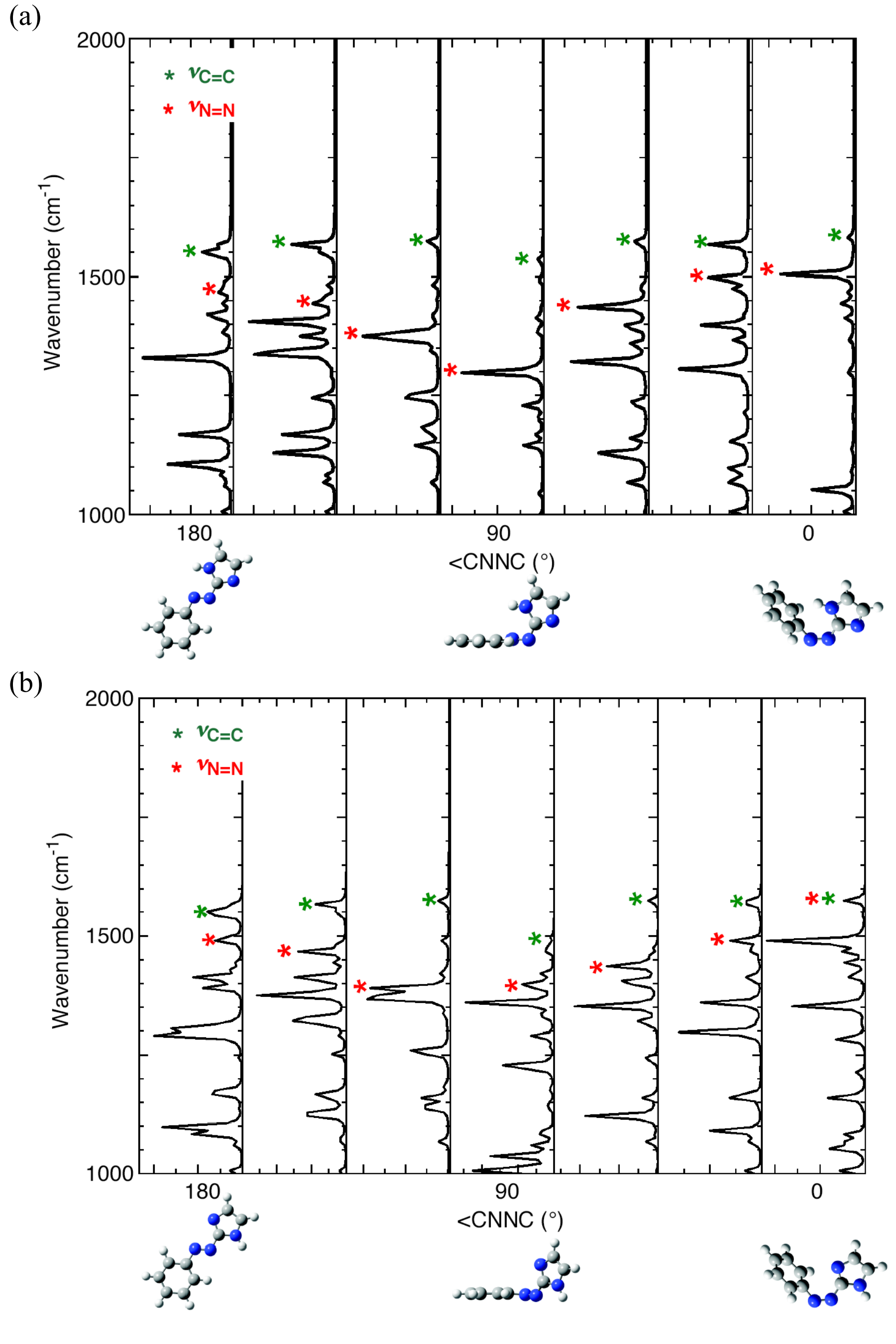

Figure S6. Calculated Raman spectral shifts of *t*-PAI in the S_1-state along the rotation pathway. Partially optimizations were performed in S_0-state. Reactant (*t*-PAI) was (a) *s-trans* conformer and (b) *s-cis* conformer.

For inversion pathway, partial optimization fixed in value of A_{NNC} was performed in S_0-state, with changing A_{NNC} from 120 to 220° with about 15° step. For each calculated partially optimized structure, the energies of S_1-state and S_2-state were calculated by single point calculation. The calculated energy profile of the inversion pathway is shown in Figure S7. The results are nearly comparable with the reported data of *t*-AB.[15,36] Thus, the structures obtained by partial optimization (Section S7; PAI-S0-I130 to PAI-S0-I220, as well as PAI-S0-IPh130 to PAI-S0-IPh240) were used for the frequency calculations.

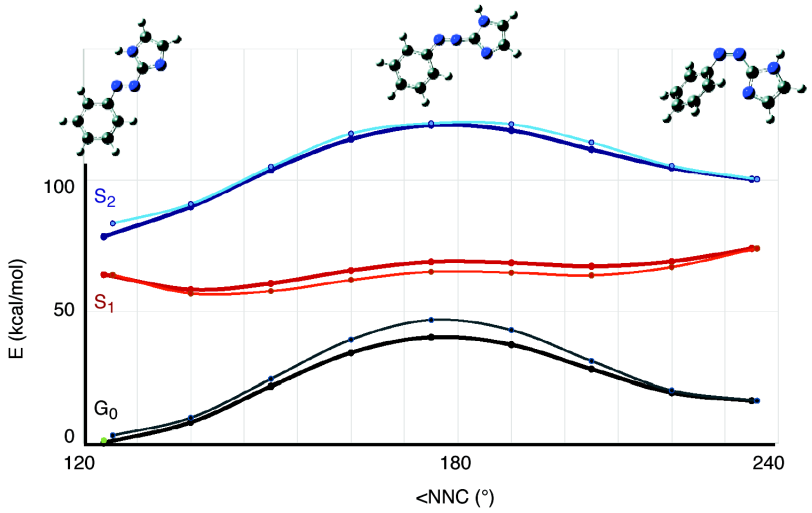

Figure S7. Calculated energy profile of the inversion pathway. Partially optimizations were performed in S_0-state. Reactant (*t*-PAI) was *s-trans* conformer (bold line) and *s-cis* conformer (thin line).

The calculation results (Figure S8) show that, regardless of whether the *s-trans* or *s-cis* conformers, under the inversion pathway, first $\nu_{N=N}$ upshifts, then downshifts. Partially optimized structures (PAI-S0-I130 ~ PAI-S0-I220 and PAI-S0-I_{Ph}130 ~ PAI-S0-I_{Ph}240) are shown in Section S7. When the reactant was *s-trans* conformer, $\nu_{N=N}$ upshifts from 1470 cm^{-1} (when A_{NNC}=180°) to 1750 cm^{-1} (when A_{NNC}=175°), then downshifts to 1575 cm^{-1} (when A_{NNC}=220°). Other hand, when the reactant was *s-cis* conformer, $\nu_{N=N}$ upshifts from 1490 cm^{-1} (when A_{NNC}=180°) to 1650 cm^{-1} (when A_{NNC}=175°), then downshifts to 1575 cm^{-1} (when A_{NNC}=220°).

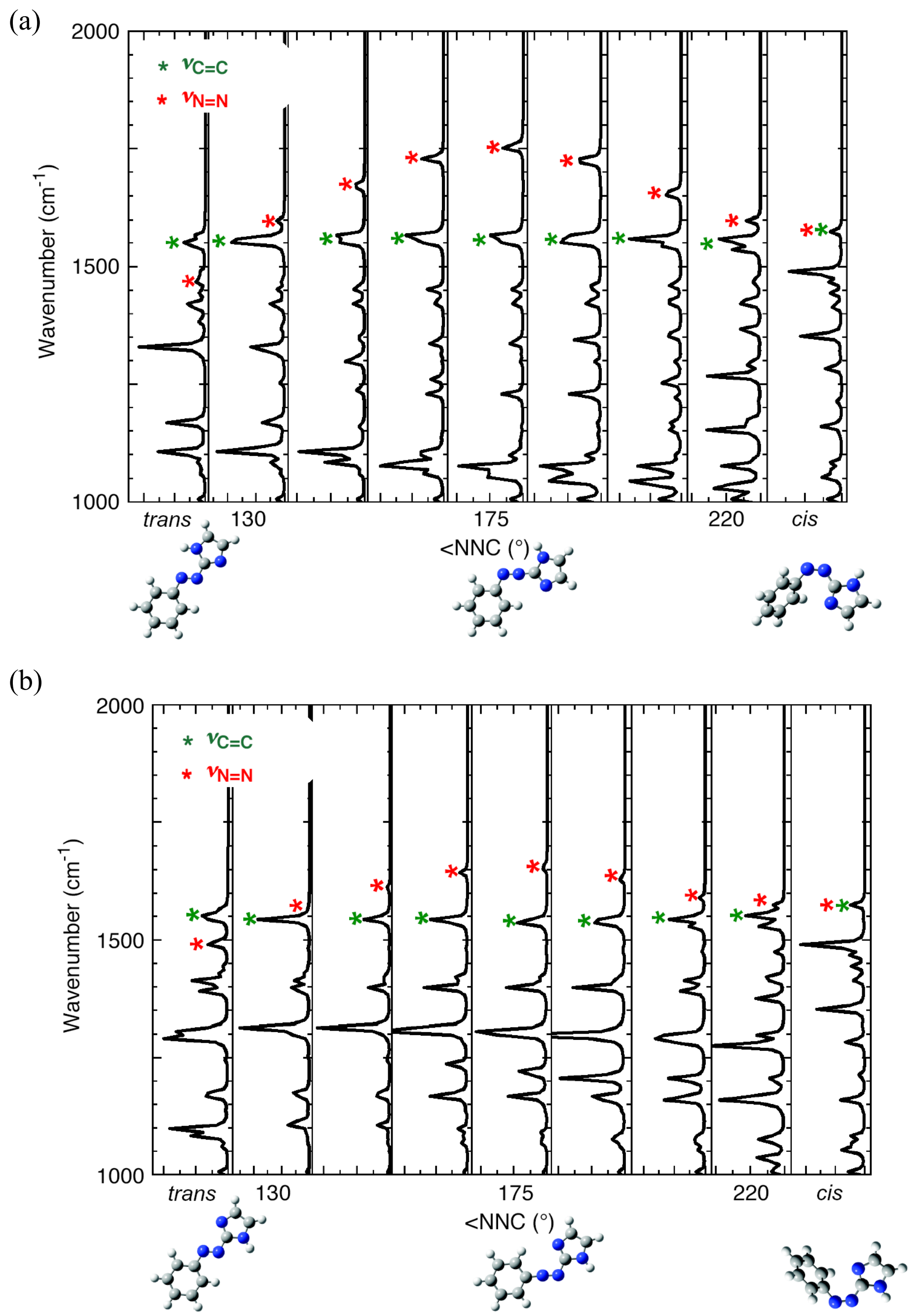

Figure S8. Calculated Raman spectral shifts of *t*-PAI in S_1-state along the inversion pathway. Partially optimizations were performed in the S_0-state. Reactant (*t*-PAI) was (a) *s-trans* conformer and (b) *s-cis* conformer.

For inversion pathway, partial optimization fixed in value of A_{NNC} was performed in S_1-state, with changing A_{NNC} from 120 to 220° with about 15° step. For each calculated partially optimized structure, the energies of S_1-state and S_2-state were calculated by single point calculation. The calculated energy profile of the inversion pathway is shown in Figure S9. The results are nearly comparable with the reported data of *t*-AB.[15,36] Thus, the structures obtained by partial optimization were used for the frequency calculations.

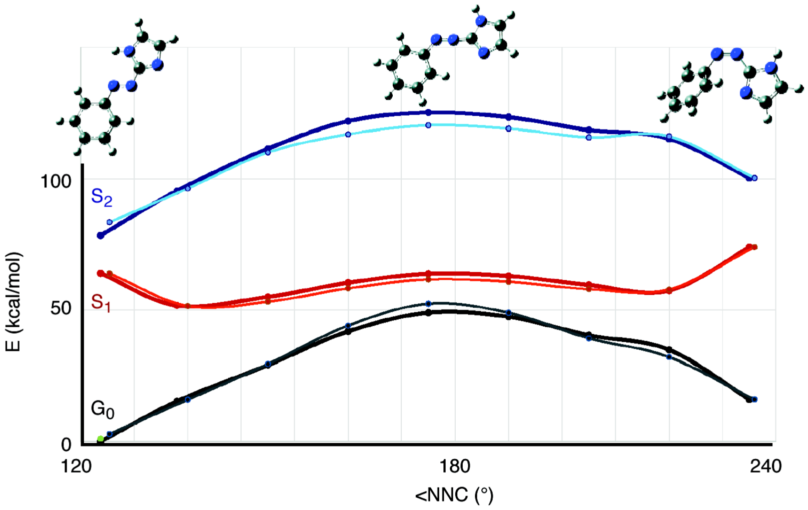

Figure S9. Calculated energy profile of the inversion pathway. Partially optimizations were performed in S_1-state. Reactant (*t*-PAI) was *s-trans* conformer (bold line) and *s-cis* conformer (thin line).

The calculation results (Figure S10) show that, regardless of whether the *s-trans* or *s-cis* conformers, under the inversion pathway, first $\nu_{N=N}$ upshifts, then downshifts. Partially optimized structures (PAI-S1-I130 ~ PAI-S1-I220 and PAI-S1-I$_{Ph}$130 ~ PAI-S1-I$_{Ph}$240) are shown in Section S7. When the reactant was *s-trans* conformer, $\nu_{N=N}$ upshifts from 1470 cm^{-1} (when A_{NNC}=180°) to 1850 cm^{-1} (when A_{NNC}=175°), then downshifts to 1575 cm^{-1} (when A_{NNC}=220°). Other hand, when the reactant was *s-cis* conformer, $\nu_{N=N}$ upshifts from 1490 cm^{-1} (when A_{NNC}=180°) to 1800 cm^{-1} (when A_{NNC}=175°), then downshifts to 1575 cm^{-1} (when A_{NNC}=220°).

(a)

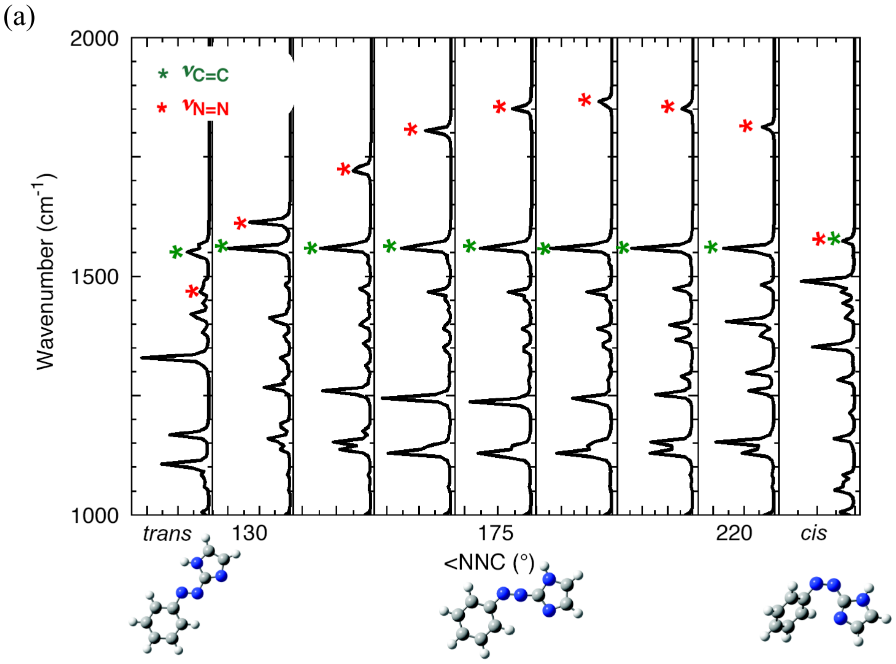

(b)

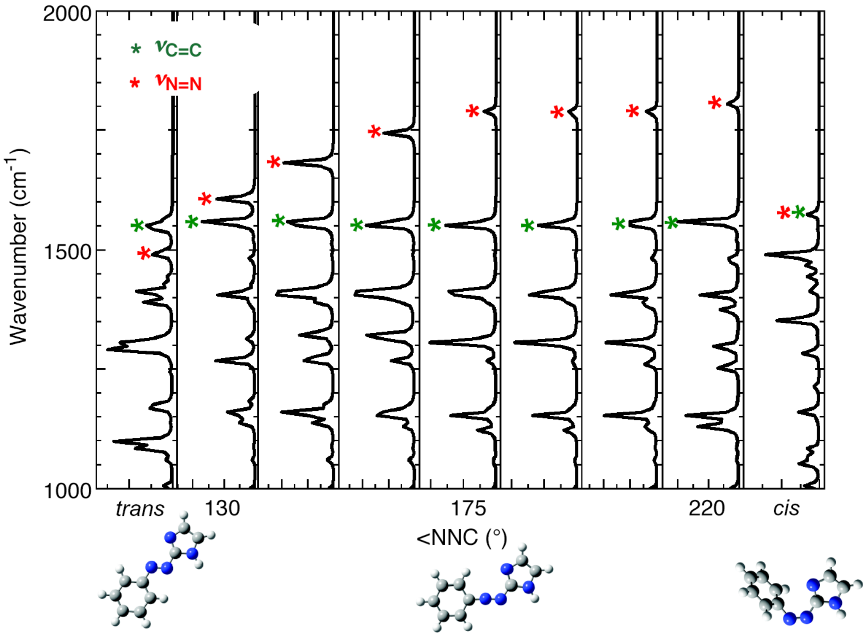

Figure S10. Calculated Raman spectral shifts of *t*-PAI in S$_1$-state along the inversion pathway. Partially optimizations were performed in S$_1$-state. Reactant (*t*-PAI) was (a) *s-trans* conformer and (b) *s-cis* conformer.

Section S4. Pump–Probe Measurement of Neat Acetonitrile using Visible 5-fs Pulse Laser

~ Background Measurement ~

S4.1. Transient Absorption Measurement

Two-dimensional Δ*A* maps of the pump–probe measurements for the forward and backward scans are shown in Figures S11a and S11b, respectively. Figure S12 presents the Δ*A* traces probed at 620 nm. The neat solvent, acetonitrile, exhibits no detectable Δ*A* signal.

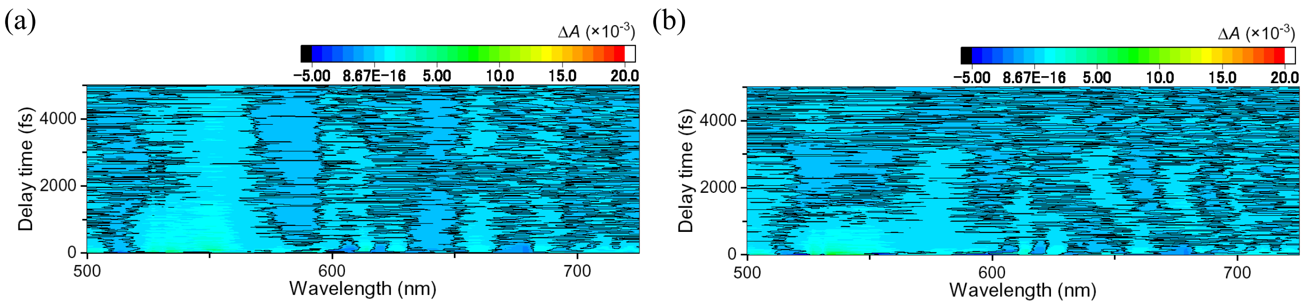

Figure S11. Two-dimensional Δ*A* map of the pump–probe measurement result for acetonitrile solvent in the scan (a) increasing and (b) decreasing the optical delay.

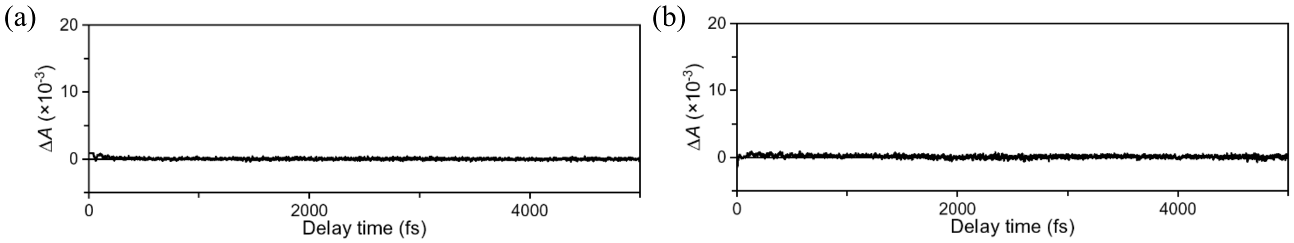

Figure S12. Δ*A* trace of acetonitrile solvent probed at 620 nm in the scan (a) increasing and (b) decreasing the optical delay.

S4.2. Spectrogram Analysis

Short-time Fourier transform (spectrogram analysis) was performed. Figure S13 shows the spectrogram traces calculated using a Blackman window function with a full width at half-maximum (FWHM) of 340 fs. The spectrogram of neat acetonitrile (Figure S13) shows a stationary ν_{CN} band near 2200 cm^{-1} with no observable frequency shift, confirming the absence of photochemical artifacts originating from the solvent.

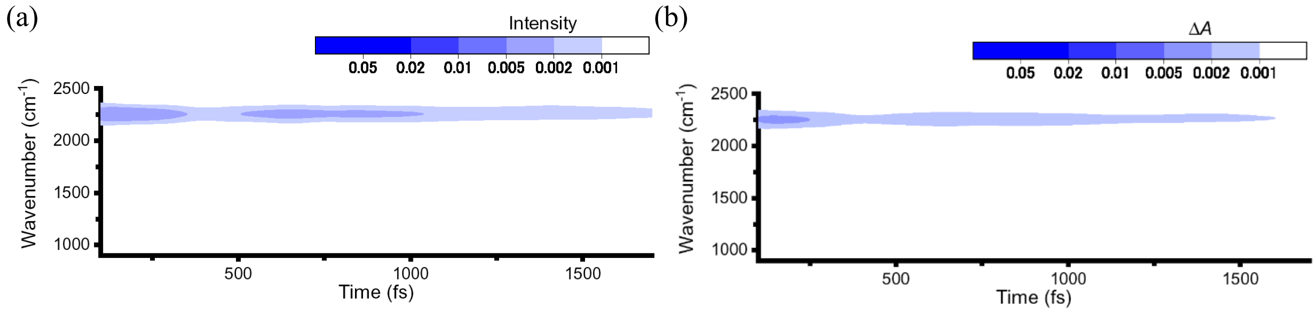

Figure S13. Spectrogram trace of acetonitrile solvent at 620 nm in the scan (a) increasing and (b) decreasing the optical delay.

Section S5. Pump–Probe Measurement of *t*-PAI using Visible 5-fs Pulse Laser

~Data Consistency Check for the Scanning Delay in the Opposite Order~

To confirm that the effect of damage accumulation during the measurement was negligible, we performed measurements to scan the delay in the reverse order (backward scan) from 5000 fs to −100 fs. The two-dimensional ΔA map measured in the backward scan (Figure S14) was comparable to that in the forward scan (Figure 3b). The amplitude of the trace measured in the backward scan (Figure S15) was the same as that in the forward scan (Figure 3c). For both scans, the oscillatory component of ΔA was extracted by subtracting the biexponential fit from the measured ΔA trace. Greatly enlarged views of the oscillatory components obtained in the forward and backward scans are shown in Figures S16a and S16b, respectively. The oscillatory signals observed in the two scan directions were comparable in both amplitude and temporal behavior. Furthermore, the spectrogram of the backward scan (Figure S16c) was the same as that of the forward scan (Figure 4). These results showed that the damage accumulation effect during the measurement was negligible.

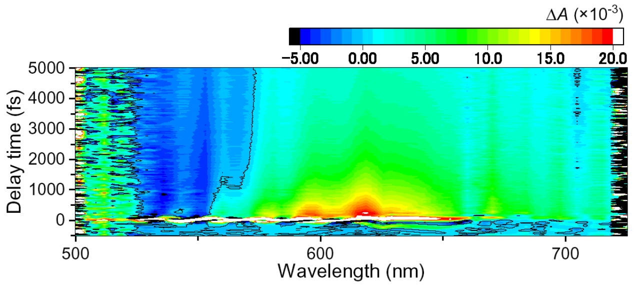

Figure S14. Two-dimensional ΔA map of the pump–probe measurement result for acetonitrile solution of *t*-PAI in the scan decreasing the optical delay.

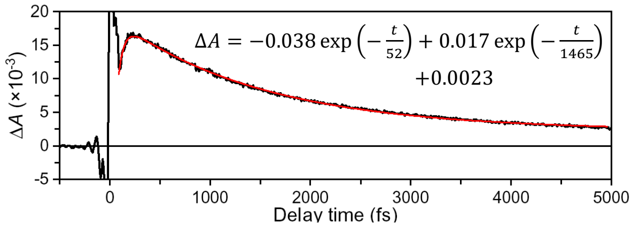

Figure S15. ΔA trace of acetonitrile solution of *t*-PAI at 620 nm (black) and its high-frequency (oscillatory) component obtained by subtracting the biexponential fit (red) in the scan decreasing the optical delay.

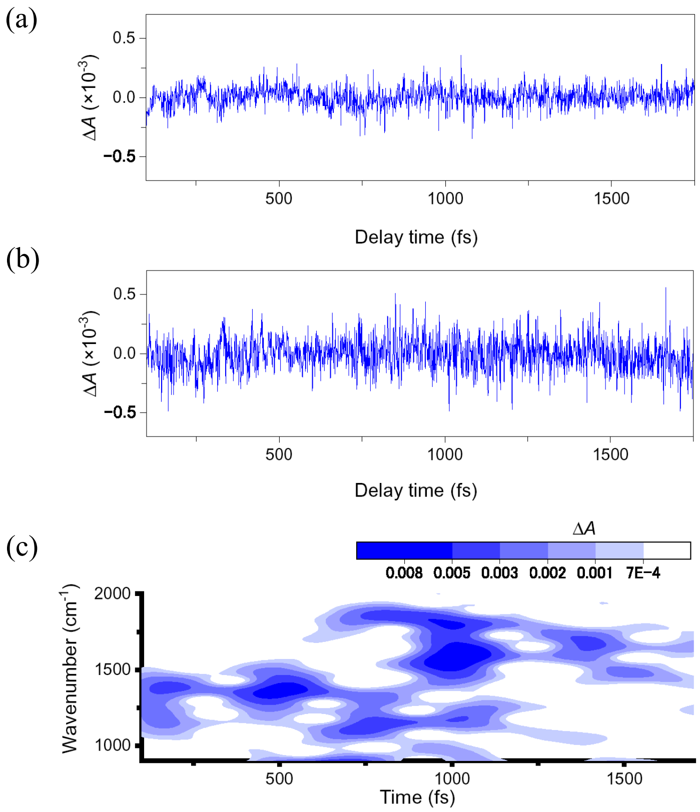

Figure S16. Comparison of the oscillatory components obtained in the forward and backward scans for acetonitrile solution of *t*-PAI probed at 620 nm. The oscillatory components were extracted by subtracting the biexponential fits from the measured ΔA traces. Greatly enlarged views of the oscillatory components obtained in (a) the forward scan, in which the optical delay was increased from −100 to 5000 fs, and (b) the backward scan, in which the optical delay was decreased from 5000 to −100 fs. (c) Spectrogram obtained by applying a short-time Fourier transform to the oscillatory component measured in the backward scan.

To verify that the ΔA signals of *t*-PAI originate from a one-photon excitation process, we investigated the dependence of the signal amplitude on pump pulse energy. The probe pulse energy was fixed at 1.5 nJ pulse^{-1}, while the pump pulse energy was varied from 7.0 to 46 nJ pulse^{-1}. At each pump pulse energy, the ΔA signal at 620 nm was obtained from the forward- and backward-scan measurements, and the resulting average value was plotted against the pump pulse energy. As shown in Figure S17, the ΔA signal increased linearly with pump pulse energy. This result confirms that, under the present experimental conditions, the observed excited-state dynamics predominantly originate from the excited-state population generated by one-photon excitation.

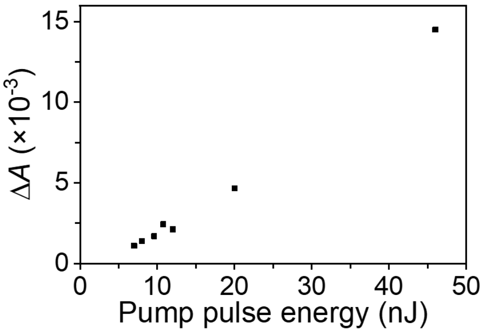

Figure S17. Pump-pulse-energy dependence of the ΔA signal probed at 620 nm.

Section S6. Pump–Probe Measurement of *t*-AB using Visible 5-fs Pulse Laser
~ Control Experiment Establishing the Quantum-Yield Masking Effect ~

To establish a direct comparison between the ultrafast vibrational dynamics of *t*-PAI and conventional azobenzene, identical pump–probe measurements were performed on an acetonitrile solution of *trans*-azobenzene (*t*-AB). This control experiment is essential for validating the quantum-yield-based interpretation presented in the main text.

S6.1. Steady-State Absorption Spectrum

Figure S18 shows the steady-state absorption spectrum of the acetonitrile solution of *t*-AB. It exhibits an intense absorption band around 320 nm corresponding to the optically allowed π–π* transition and a weak absorption band around 445 nm for the optically forbidden n–π* transition. The generated visible 5-fs pulse laser covers the spectral range of 500–750 nm, enabling n–π* excitation via a single-photon process, identical to the excitation scheme employed for *t*-PAI.

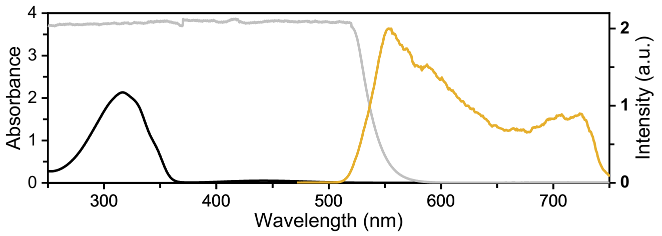

Figure S18. Steady-state absorption spectrum of acetonitrile solution of *t*-AB measured with a 1-mm optical path length at concentrations of 1.0×10^{-3} mol L^{-1} (black) and at 1.0 mol L^{-1} (gray). Orange curves show the spectrum of the visible 5-fs pulse laser.

S6.2. Transient Absorption Measurement

Two-dimensional ΔA maps of the pump–probe measurement results for forward and backward scans are shown in Figures S19a and S19b, respectively. In the entire probe wavelength region, the transient absorption signal ΔA is observed with a positive sign, assigned to induced absorption. The neat solvent acetonitrile does not show positive ΔA (Figure S11), confirming that the observed signal originates from *t*-AB. Comparison between forward and backward scans shows no significant difference, confirming that the measurement is not affected by accumulation of the *cis* isomer during the measurement time (seconds to hours).

Figure S20 shows the ΔA traces probed at 620 nm. Biexponential fitting yields lifetimes of $\tau_1 \approx 150$ fs and $\tau_2 \approx 1200$ fs. In various solutions of *t*-AB, relaxation from the Franck–Condon state to the most stable structure in Sn–π* state has been reported to occur in 180–600 fs, followed by ground-state recovery in 600–3000 fs depending on the solvent,[23,42] which are comparable with the values estimated here. The solvent signal was confirmed to be constant (Figure S12).

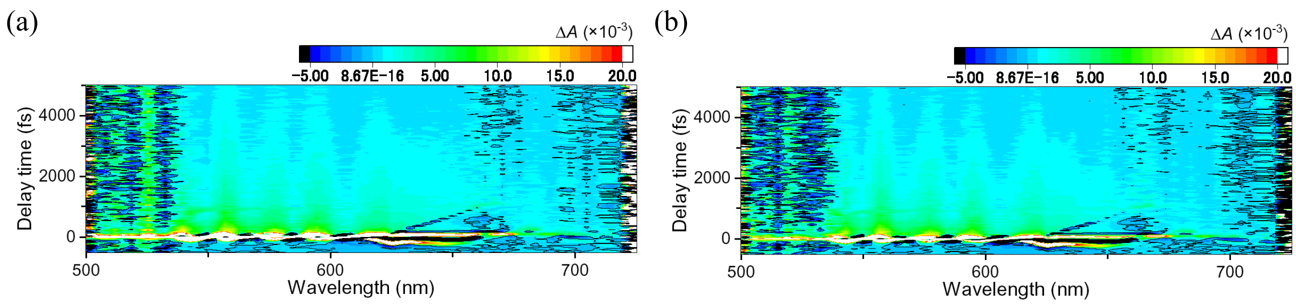

Figure S19. Two-dimensional ΔA map of the pump–probe measurement result for acetonitrile solution of *t*-AB in the scan (a) increasing and (b) decreasing the optical delay.

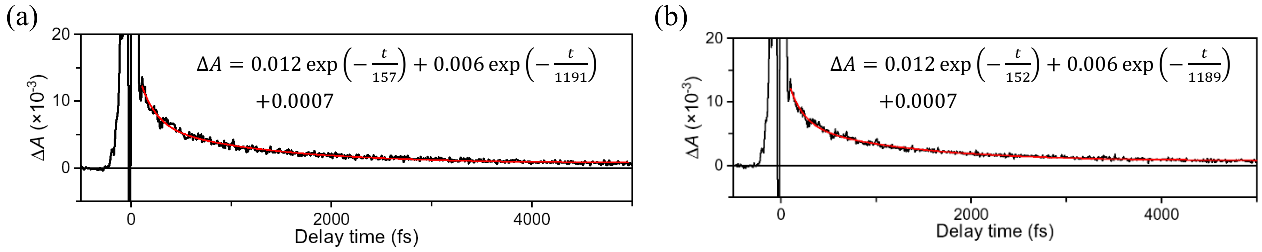

Figure S20. ΔA trace of acetonitrile solution of *t*-AB at 620 nm (black) and its high-frequency (oscillatory) component obtained by subtracting the biexponential fit (red) in the scan (a) increasing and (b) decreasing the optical delay.

S1.3. Spectrogram Analysis

The exponential decay curve was subtracted from the ΔA traces to extract high-frequency modulations reflecting molecular vibrations in the time domain. Short-time Fourier transform (spectrogram analysis) was then performed.

Figure S21 shows spectrogram trace calculated using a Blackman window function with a FWHM of 340 fs. The spectrogram of the acetonitrile solution of *t*-AB shows, immediately after photoexcitation, signals of C=C stretching mode ($\nu_{C=C}$) at around 1500 cm^{-1}, C–N stretching mode (ν_{CN}) at around 1100 cm^{-1}, and benzene ring stretching mode (ν_{ring}) at around 1000 cm^{-1} (experimental and calculated Raman spectra of S_0-state are shown in Figure S4).

Critically, none of these modes exhibit a significant frequency shift in the $\nu_{N=N}$ region — in stark contrast to the pronounced ~400 cm^{-1} upshift observed for *t*-PAI under identical conditions (Figure 4 in the main text). This absence of a $\nu_{N=N}$ upshift in *t*-AB is consistent with the quantum-yield masking hypothesis: with a quantum yield of only ~0.31 for n–π*-induced *trans*-to-*cis* isomerization in acetonitrile (and ca. 0.24 in nonpolar solvent under n–π* excitation),[47,48] the dominant non-reactive population (~70–80%) that relaxes from S_1-state back to the *trans* S_0-state produces vibrational signals near the S_0-state frequencies, effectively obscuring any frequency shift originating from the minority reactive population.

The only detectable frequency change in *t*-AB is a modest upshift of ν_{CN} band, which can be explained by TD-DFT calculations comparing the Raman spectra at the Franck–Condon geometry and the S_1 minimum (Figure S22). The calculation predicts that ν_{CN} shifts from 1075 to 1130 cm^{-1} as the C–N bond shortens from 1.42 to 1.36 Å upon relaxation to the S_1 minimum. This shift reflects the non-reactive relaxation dynamics common to all photoexcited molecules — both reactive and non-reactive — and does not constitute evidence for or against the inversion pathway. As a reference, the spectrogram of neat acetonitrile (Figure S13) exhibits a stationary ν_{CN} band near 2200 cm^{-1} with no frequency shift, confirming the absence of photochemical artifacts from the solvent.

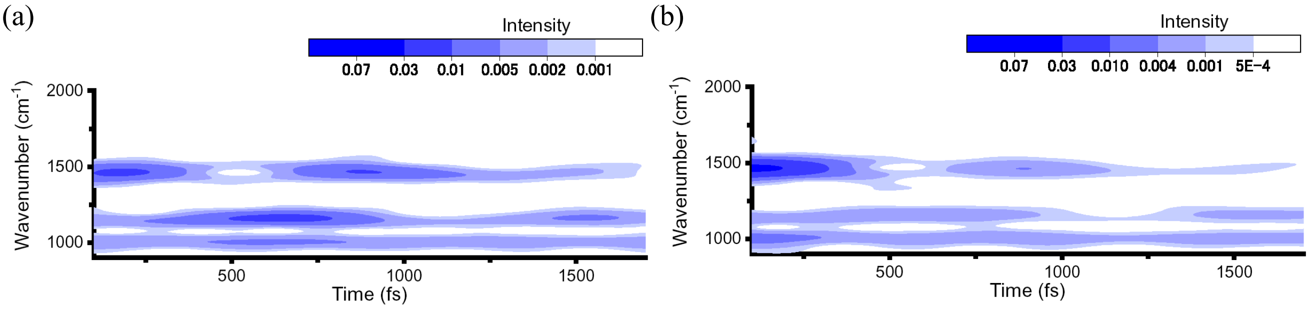

Figure S21. Spectrogram trace of acetonitrile solution of *t*-AB probed at 620 nm in the scan (a) increasing and (b) decreasing the optical delay.

The molecular vibrational frequency of acetonitrile, which are not excited under irradiation of visible 5-fs pulse laser, does not shift at all, whereas the molecular vibrational frequency of *t*-AB, which are excited under irradiation of visible 5-fs pulse laser, shifts slightly. Then, we have performed TD-DFT calculation to compare Raman spectra between Franck-Condon state and most stable structure in Sn–π* (Figure S22). The result shows that ν_{CN} shifts from 1075 to 1130 cm^{-1}, because the C–N bond length become shorter (from 1.42 to 1.36 Å) and the bond order becomes increase. Therefore, ν_{CN} appeared around 1100 cm^{-1} has upshifted at several hundred femtoseconds after the photoexcitation.

As demonstrated above, when main reaction is the relaxation from Sn–π* to S_0-state, it is hard to analyze molecular vibrational frequency change during the photoisomerization.

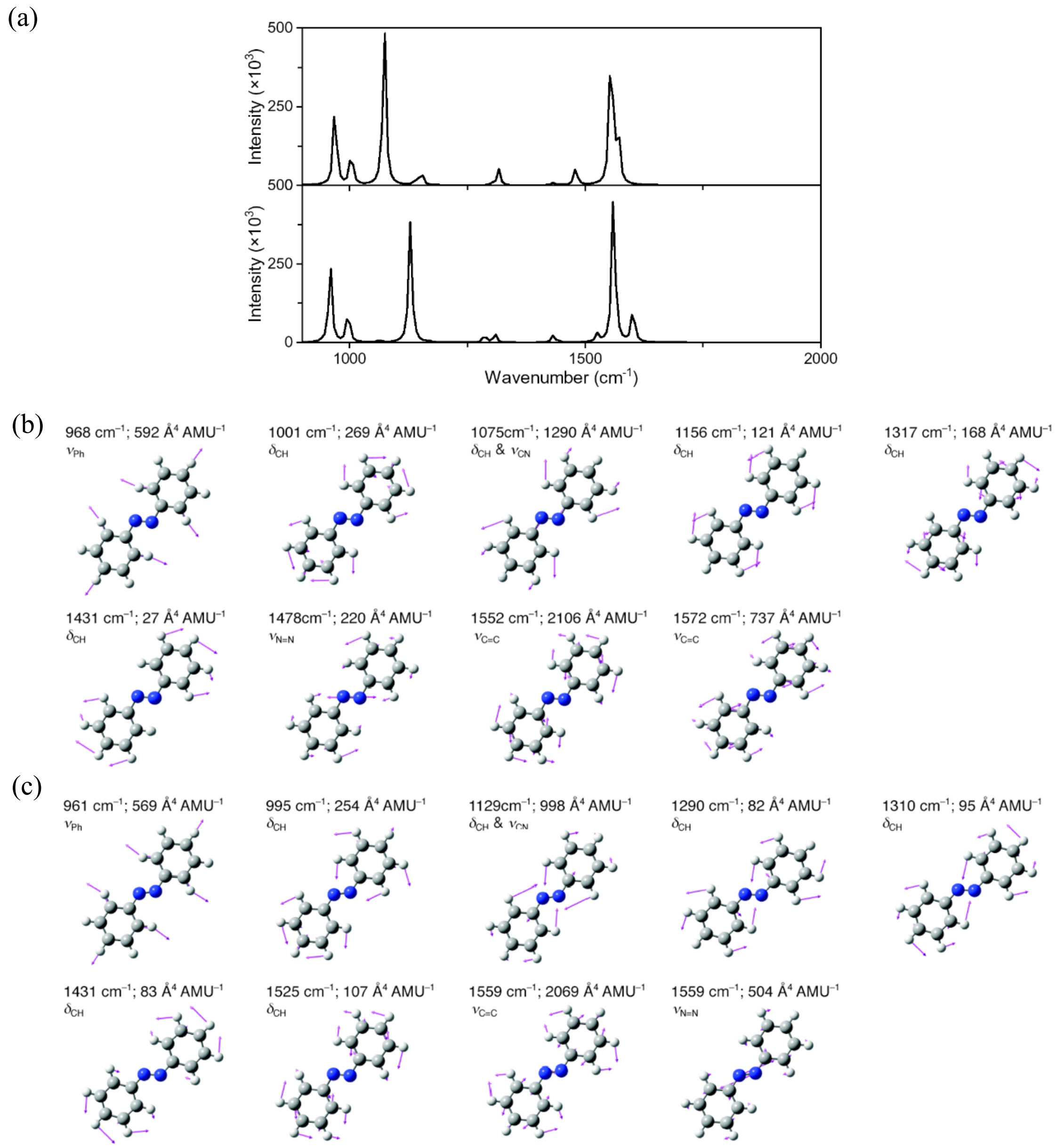

Figure S22. Calculated results of Raman-active molecular vibrational frequencies. (a) Raman spectra of *t*-AB in Sn–π* state for the Franck–Condon geometry (upper panel) and the optimized S_1 minimum (lower panel). (b) Raman-active vibrational modes of *t*-AB in Sn–π* state at the Franck–Condon geometry and (c) the optimized S_1 minimum structure.

Section S7. Optimized Structure

t-AB-S0

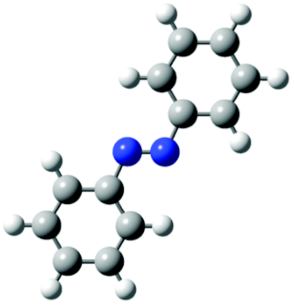

N,0,-0.0127868656,-0.3623075417,0.0218290645
N,0,0.1234526456,-0.3411320402,1.2721823392
C,0,1.1817119684,-0.1737286852,-0.7215873914
C,0,2.4547560923,0.0268259639,-0.1557726302
C,0,3.5564651359,0.2009206938,-0.9887450247
C,0,3.4053385542,0.1775361514,-2.3834725933
C,0,2.1411296543,-0.0216516245,-2.945315714
C,0,1.0315063506,-0.1969070224,-2.1161884826
H,0,0.0383424882,-0.3533418023,-2.5275953271
H,0,2.0195764801,-0.0404582843,-4.0251172284
H,0,4.271721954,0.3144113413,-3.0255476289
H,0,4.540990815,0.3561245664,-0.5543529536
H,0,2.5574812445,0.0427435843,0.9237469335
C,0,-1.0711108267,-0.5292821705,2.0156027174
C,0,-0.9210021852,-0.5054557156,3.410203625
C,0,-2.0307222869,-0.6800728755,4.239335464
C,0,-3.294941988,-0.8792078468,3.6774972699
C,0,-3.4459799027,-0.903186893,2.2827703519
C,0,-2.3441488426,-0.7298887462,1.4497929858
H,0,-2.4468283144,-0.7461285256,0.3702738695
H,0,-4.4305376204,-1.0581984113,1.8483820676
H,0,-4.1614148958,-1.0155012825,4.319575252
H,0,-1.9092368583,-0.6608081203,5.3191365451
H,0,0.0721562619,-0.3489757157,3.8216064116

Overview Tab Data Section:
Calculation Method = RB3LYP
Formula = C12H10N2
Basis Set = 6-31+G(d)
Charge = 0
Spin = Singlet
Solvation = None
E(RB3LYP) = -572.7729 Hartree
RMS Gradient Norm = 1.0801e-05 Hartree/Bohr
Imaginary Freq = 0
Dipole Moment = 0.0010198039 Debye
Polarizability (?) = 182.206 a.u.

Thermo Tab Data Section:
Imaginary Freq = 0
Temperature = 298.15 Kelvin
Pressure = 1 atm
Electronic Energy (EE) = -572.7729 Hartree
Zero-point Energy Correction = 0.190845 Hartree
Thermal Correction to Energy = 0.201701 Hartree
Thermal Correction to Enthalpy = 0.202645 Hartree
Thermal Correction to Free Energy = 0.15245 Hartree
EE + Zero-point Energy = -572.58205 Hartree
EE + Thermal Energy Correction = -572.5712 Hartree
EE + Thermal Enthalpy Correction = -572.57025 Hartree
EE + Thermal Free Energy Correction = -572.62045 Hartree

Opt Tab Data Section:
Maximum force = 3.3e-05 Converged
RMS force = 6e-06 Converged
Maximum displacement = 0.001155 Converged
RMS displacement = 0.00033 Converged
Predicted energy change = -1.472078e-08 Hartree

t-AB-S1

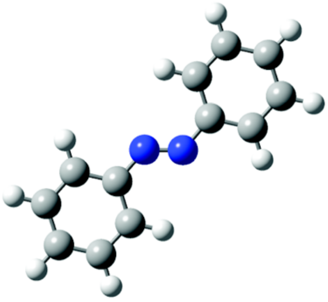

N,0,0.1294544661,-0.0001534127,0.0171345726
N,0,-0.129449625,0.0000063133,1.2408066703
C,0,1.3257423678,-0.0000216239,-0.6301627778
C,0,2.5644144968,0.0003698317,0.0702380367
C,0,3.7538436969,0.0004585757,-0.6475051038
C,0,3.754919398,0.0001716357,-2.0503644303
C,0,2.5315143114,-0.0002132154,-2.7387572749
C,0,1.3265393096,-0.0003163946,-2.048712699
H,0,0.3778679509,-0.0006207415,-2.5766641999
H,0,2.5206853735,-0.0004371413,-3.8257176182
H,0,4.692795696,0.0002453584,-2.597796914
H,0,4.6965777247,0.0007605067,-0.1054647568
H,0,2.5585715432,0.0005878417,1.155305797
C,0,-1.3257374315,0.0005570798,1.8881039179
C,0,-1.3265344831,0.0005601793,3.3066538642
C,0,-2.5315094388,0.0010943605,3.9966983195
C,0,-3.7549143854,0.0016255405,3.3083053319
C,0,-3.7538385901,0.0016176609,1.9054459774
C,0,-2.5644094262,0.0010953166,1.187702963
H,0,-2.5585664067,0.001083886,0.1026351801
H,0,-4.6965725088,0.002029435,1.3634055118
H,0,-4.6927906497,0.0020368881,3.8557377247
H,0,-2.5206805768,0.001095863,5.0836586863
H,0,-0.377863242,0.000141363,3.8346054927

Overview Tab Data Section:
Calculation Method = RB3LYP TD-FC
Formula = C12H10N2
Basis Set = 6-31+G(d)
Charge = 0
Spin = Singlet
Solvation = None
E(TD-HF/TD-DFT) = -572.69926 Hartree
RMS Gradient Norm = 6.783e-06 Hartree/Bohr
Imaginary Freq = 0
Dipole Moment = 0.001 Debye
Polarizability (?) = 213.932 a.u.

Thermo Tab Data Section:
Imaginary Freq = 0
Temperature = 298.15 Kelvin
Pressure = 1 atm
Electronic Energy (EE) = -572.69926 Hartree
Zero-point Energy Correction = 0.189507 Hartree
Thermal Correction to Energy = 0.200495 Hartree
Thermal Correction to Enthalpy = 0.201439 Hartree
Thermal Correction to Free Energy = 0.15099 Hartree
EE + Zero-point Energy = -572.50975 Hartree
EE + Thermal Energy Correction = -572.49876 Hartree
EE + Thermal Enthalpy Correction = -572.49782 Hartree
EE + Thermal Free Energy Correction = -572.54827 Hartree

Opt Tab Data Section:
Maximum force = 2e-05 Converged
RMS force = 4e-06 Converged
Maximum displacement = 0.001551 Converged
RMS displacement = 0.000421 Converged
Predicted energy change = -5.008367e-09 Hartree

CH_3CN

C,0,-0.0161909635,0.0280553961,-0.0114489598
H,0,-0.0089489605,0.0154840877,1.0832476319
H,0,1.0183134104,0.0154806038,-0.3695184768
H,0,-0.5225648334,-0.8741356746,-0.3695097901
C,0,-0.7056479428,1.2222226822,-0.4989702608
N,0,-1.252997567,2.1702460165,-0.8859982551

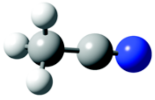

Overview Tab Data Section:
Calculation Method = RB3LYP
Formula = C2H3N
Basis Set = 6-31+G(d)
Charge = 0
Spin = Singlet
Solvation = None
E(RB3LYP) = -132.75853 Hartree
RMS Gradient Norm = 5.359e-06 Hartree/Bohr
Imaginary Freq = 0
Dipole Moment = 4.0725168 Debye
Polarizability (?) = 26.409667 a.u.

Thermo Tab Data Section:
Imaginary Freq = 0
Temperature = 298.15 Kelvin
Pressure = 1 atm
Electronic Energy (EE) = -132.75853 Hartree
Zero-point Energy Correction = 0.045479 Hartree
Thermal Correction to Energy = 0.049094 Hartree
Thermal Correction to Enthalpy = 0.050038 Hartree
Thermal Correction to Free Energy = 0.021445 Hartree
EE + Zero-point Energy = -132.71305 Hartree
EE + Thermal Energy Correction = -132.70943 Hartree
EE + Thermal Enthalpy Correction = -132.70849 Hartree
EE + Thermal Free Energy Correction = -132.73708 Hartree

Opt Tab Data Section:
Maximum force = 1.3e-05 Converged
RMS force = 4e-06 Converged
Maximum displacement = 2.2e-05 Converged
RMS displacement = 1.2e-05 Converged
Predicted energy change = -3.428248e-10 Hartree

t-PAI-S0 (*s*-*trans*)

C,0,0.,0.,0.
C,0,0.,0.,2.7941382337
C,0,1.2149254359,0.,0.7030255492
C,0,-1.2115388483,-0.0000014248,0.6929671251
C,0,-1.2142044319,-0.0000013484,2.0908845823
C,0,1.212740124,0.0000004784,2.1113123328
N,0,2.3834760652,-0.0000003385,-0.0941866782
N,0,3.475426867,-0.0000106994,0.5470336087
C,0,4.5968948677,-0.0000101693,-0.2630952766
N,0,5.8409002368,-0.000021276,0.2017531087
C,0,6.6410744701,-0.0000167782,-0.9035695053
C,0,5.8782978149,-0.0000024106,-2.0608303561
N,0,4.5767486818,0.0000017636,-1.6370541136
H,0,3.7309905802,0.0000125656,-2.1919390472
H,0,6.1469255849,0.0000056537,-3.1075353838
H,0,0.0271945981,0.0000000526,-1.0860817991
H,0,-2.1496994987,-0.0000024565,0.1444655105
H,0,-2.1558003428,-0.0000025914,2.63385537
H,0,-0.0048272079,-0.0000087106,3.8811979367
H,0,2.1584071197,0.0000002608,2.6424513778
H,0,7.7194132575,-0.000024162,-0.8206092909

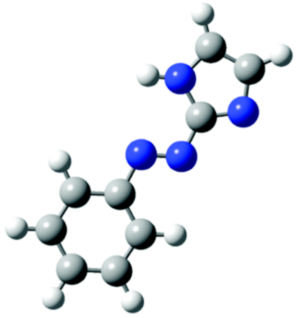

Overview Tab Data Section:
Calculation Method = RB3LYP
Formula = C9H8N4
Basis Set = 6-31+G(d)
Charge = 0
Spin = Singlet
Solvation = None
E(RB3LYP) = -566.7465 Hartree
RMS Gradient Norm = 4.88e-07 Hartree/Bohr
Imaginary Freq = 0
Dipole Moment = 3.6228858 Debye
Polarizability (?) = 166.39633 a.u.

Thermo Tab Data Section:
Imaginary Freq = 0
Temperature = 298.15 Kelvin
Pressure = 1 atm
Electronic Energy (EE) = -566.7465 Hartree
Zero-point Energy Correction = 0.161867 Hartree
Thermal Correction to Energy = 0.171793 Hartree
Thermal Correction to Enthalpy = 0.172737 Hartree
Thermal Correction to Free Energy = 0.125095 Hartree
EE + Zero-point Energy = -566.58463 Hartree
EE + Thermal Energy Correction = -566.57471 Hartree
EE + Thermal Enthalpy Correction = -566.57376 Hartree
EE + Thermal Free Energy Correction = -566.6214 Hartree

Opt Tab Data Section:
Maximum force = 6e-06 Converged
RMS force = 1e-06 Converged
Maximum displacement = 0.000131 Converged
RMS displacement = 4.2e-05 Converged
Predicted energy change = -1.679195e-10 Hartree

t-PAI-S0 (s-cis)

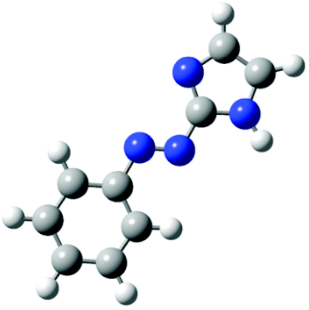

C,0,0.,0.,0.
C,0,0.,0.,2.7948923518
C,0,1.2156631108,0.,0.7023818719
C,0,-1.2113895773,0.0001633112,0.6933191723
C,0,-1.2141498197,0.0001386166,2.0912251019
C,0,1.2120747363,-0.0000497942,2.1108165143
N,0,2.3744800495,-0.0001006035,-0.1093297052
N,0,3.4685361855,0.0001685921,0.5207687862
C,0,4.6089264264,0.0000265047,-0.2679989732
N,0,4.7808788253,-0.0004488302,-1.5842950605
C,0,6.1319702242,-0.0003559433,-1.7717291715
C,0,6.802912734,0.0001721752,-0.5583185204
N,0,5.8167409656,0.0003968021,0.3899570077
H,0,5.9235299861,0.0007904592,1.3956632472
H,0,7.8531253198,0.0003851117,-0.3062240583
H,0,0.0330930916,-0.0000604299,-1.0856141356
H,0,-2.1496893665,0.0002221778,0.1450489251
H,0,-2.1556719659,0.000117785,2.6344962324
H,0,-0.0055704622,-0.00069473,3.8821537198
H,0,2.1562011302,-0.0002453655,2.6446464144
H,0,6.5625636651,-0.0006831922,-2.7644057037

Overview Tab Data Section:
Calculation Method = RB3LYP
Formula = C9H8N4
Basis Set = 6-31+G(d)
Charge = 0
Spin = Singlet
Solvation = None
E(RB3LYP) = -566.74195 Hartree
RMS Gradient Norm = 6.558e-06 Hartree/Bohr
Imaginary Freq = 0
Dipole Moment = 3.5699837 Debye
Polarizability (?) = 167.24167 a.u.

Thermo Tab Data Section:
Imaginary Freq = 0
Temperature = 298.15 Kelvin
Pressure = 1 atm
Electronic Energy (EE) = -566.74195 Hartree
Zero-point Energy Correction = 0.161632 Hartree
Thermal Correction to Energy = 0.17167 Hartree
Thermal Correction to Enthalpy = 0.172615 Hartree
Thermal Correction to Free Energy = 0.124529 Hartree
EE + Zero-point Energy = -566.58032 Hartree
EE + Thermal Energy Correction = -566.57028 Hartree
EE + Thermal Enthalpy Correction = -566.56933 Hartree
EE + Thermal Free Energy Correction = -566.61742 Hartree

Opt Tab Data Section:
Maximum force = 1.5e-05 Converged
RMS force = 3e-06 Converged
Maximum displacement = 0.001545 Converged
RMS displacement = 0.0003 Converged
Predicted energy change = -1.992631e-08 Hartree

c-PAI-S0 (*s*-*cis*)

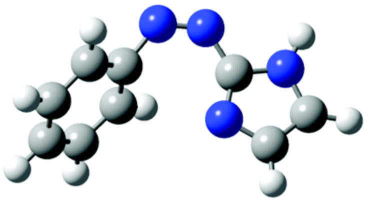

C,0,0.2218948515,-0.028839499,0.0389215828
C,0,0.0761474508,0.0888227926,2.8268168656
C,0,1.3662375923,-0.2639618321,0.8131657258
C,0,-0.9767263461,0.3242492517,0.6607093845
C,0,-1.0556725625,0.3794843191,2.0558163699
C,0,1.2875287392,-0.2245656225,2.2132758429
N,0,2.5245297363,-0.7484663472,0.1308560215
N,0,3.6755858444,-0.2591885662,0.248955536
C,0,3.9876038266,0.9359157722,0.9083686568
N,0,3.2680783058,1.9419977324,1.3944200461
C,0,4.1734789345,2.8864314117,1.7808757767
C,0,5.4691848317,2.4588485712,1.5333135938
N,0,5.3325044369,1.2222893453,0.9714369008
H,0,6.0596399589,0.6101224882,0.6241342487
H,0,6.431519699,2.9169698102,1.7081071563
H,0,0.2884243375,-0.1142330329,-1.0420446336
H,0,-1.8545672073,0.5351307787,0.0553050751
H,0,-1.9950161249,0.6325928773,2.5402616059
H,0,0.0158201644,0.1119680272,3.9120403599
H,0,2.166547841,-0.443159062,2.8118678871
H,0,3.8568706818,3.824613761,2.2169739567

Overview Tab Data Section:
Calculation Method = RB3LYP
Formula = C9H8N4
Basis Set = 6-31+G(d)
Charge = 0
Spin = Singlet
Solvation = None
E(RB3LYP) = -566.7209 Hartree
RMS Gradient Norm = 1.982e-06 Hartree/Bohr
Imaginary Freq = 0
Dipole Moment = 3.5711352 Debye
Polarizability (?) = 145.70967 a.u.

Thermo Tab Data Section:
Imaginary Freq = 0
Temperature = 298.15 Kelvin
Pressure = 1 atm
Electronic Energy (EE) = -566.7209 Hartree
Zero-point Energy Correction = 0.161182 Hartree
Thermal Correction to Energy = 0.171249 Hartree
Thermal Correction to Enthalpy = 0.172193 Hartree
Thermal Correction to Free Energy = 0.123674 Hartree
EE + Zero-point Energy = -566.55972 Hartree
EE + Thermal Energy Correction = -566.54965 Hartree
EE + Thermal Enthalpy Correction = -566.54871 Hartree
EE + Thermal Free Energy Correction = -566.59723 Hartree

Opt Tab Data Section:
Maximum force = 8e-06 Converged
RMS force = 1e-06 Converged
Maximum displacement = 0.00118 Converged
RMS displacement = 0.000278 Converged
Predicted energy change = -1.517264e-09 Hartree

c-PAI-S0 (*s-trans*)

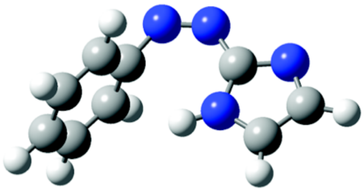

C,0,0.4780411472,0.2186913358,0.102762026
C,0,-0.0959422591,-0.1093616461,2.8225371318
C,0,1.4540703395,-0.2961522226,0.970317387
C,0,-0.772067004,0.587506653,0.6074900915
C,0,-1.0634565469,0.4289679158,1.9668953902
C,0,1.1578127314,-0.4819551159,2.3297346917
N,0,2.685240124,-0.8031143614,0.4349531304
N,0,3.7356238933,-0.1236600979,0.327286214
C,0,3.8846545002,1.2150868487,0.7025719143
N,0,5.0633488077,1.8091907537,0.5278635918
C,0,4.9162865484,3.0837975611,0.9741886302
C,0,3.6259805094,3.2912399603,1.4355875233
N,0,2.9778577741,2.1021033612,1.2601391603
H,0,2.0144224654,1.9052447105,1.4940490167
H,0,3.1344962484,4.1560346747,1.8587611383
H,0,0.7041405894,0.3226653659,-0.9549773767
H,0,-1.5224665269,0.9914196581,-0.067324411
H,0,-2.0391317929,0.7101904273,2.3531874509
H,0,-0.3184216883,-0.2495898331,3.8772689153
H,0,1.9069553936,-0.9171238277,2.9856006169
H,0,5.7332757634,3.7919308258,0.9463227874

Overview Tab Data Section:
Calculation Method = RB3LYP
Formula = C9H8N4
Basis Set = 6-31+G(d)
Charge = 0
Spin = Singlet
Solvation = None
E(RB3LYP) = -566.71775 Hartree
RMS Gradient Norm = 4.52e-07 Hartree/Bohr
Imaginary Freq = 0
Dipole Moment = 6.3571253 Debye
Polarizability (?) = 138.29167 a.u.

Thermo Tab Data Section:
Imaginary Freq = 0
Temperature = 298.15 Kelvin
Pressure = 1 atm
Electronic Energy (EE) = -566.71775 Hartree
Zero-point Energy Correction = 0.161062 Hartree
Thermal Correction to Energy = 0.171176 Hartree
Thermal Correction to Enthalpy = 0.17212 Hartree
Thermal Correction to Free Energy = 0.122342 Hartree
EE + Zero-point Energy = -566.55669 Hartree
EE + Thermal Energy Correction = -566.54657 Hartree
EE + Thermal Enthalpy Correction = -566.54563 Hartree
EE + Thermal Free Energy Correction = -566.59541 Hartree

Opt Tab Data Section:
Maximum force = 2e-06 Converged
RMS force = 0 Converged
Maximum displacement = 5.9e-05 Converged
RMS displacement = 1.3e-05 Converged
Predicted energy change = -3.036037e-11 Hartree

PAI-S0-R150 (*s-trans*; Φ_{CNNC} = 150°)

C
C,1,B1
C,1,B2,2,A1
C,1,B3,3,A2,2,D1,0
C,4,B4,1,A3,3,D2,0
C,2,B5,1,A4,4,D3,0
N,3,B6,1,A5,4,D4,0
N,7,B7,3,A6,1,D5,0
C,8,B8,7,A7,3,-149.9999784,0
N,9,B9,8,A8,7,D7,0
C,10,B10,9,A9,8,D8,0
C,11,B11,10,A10,9,D9,0
N,12,B12,11,A11,10,D10,0
H,13,B13,12,A12,11,D11,0
H,12,B14,11,A13,10,D12,0
H,1,B15,4,A14,5,D13,0
H,4,B16,1,A15,3,D14,0
H,5,B17,4,A16,1,D15,0
H,2,B18,1,A17,4,D16,0
H,6,B19,2,A18,1,D17,0
H,11,B20,10,A19,9,D18,0
Variables:
B1=2.79568664
B2=1.40658977
B3=1.39404821
B4=1.39940234
B5=1.39223733
B6=1.40768536
B7=1.27254017
B8=1.37752229
B9=1.33070063
B10=1.36246684
B11=1.38760401
B12=1.36759904
B13=1.01174647
B14=1.08062881
B15=1.08645546
B16=1.08678238
B17=1.08687899
B18=1.08708888
B19=1.08468375
B20=1.08162951
A1=59.96945003
A2=120.15992948
A3=119.95971613
A4=60.66102902
A5=116.16303705
A6=115.78908494
A7=114.12597166
A8=123.95509878
A9=105.41183671
A10=110.78984564
A11=105.33890088
A12=128.7918559
A13=132.17108123
A14=121.23740448
A15=119.89738692
A16=120.0662895
A17=179.43498062
A18=121.33792206
A19=121.51120345
D1=-0.16169197
D2=0.17675339
D3=179.8428565
D4=-177.49074496
D5=-172.98591789
D7=-178.2338565
D8=182.14989138
D9=-0.14931939
D10=-0.01170759
D11=-179.81455535
D12=-181.60617046
D13=-179.78492909
D14=-179.97147776
D15=179.41227044
D16=-63.52021087
D17=179.07159356
D18=-180.76382952

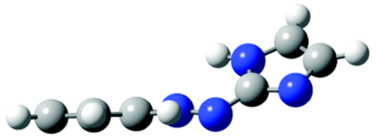

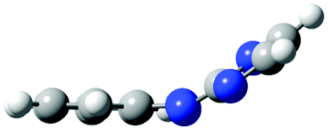

Overview Tab Data Section:
Calculation Method = RB3LYP
Formula = C9H8N4
Basis Set = 6-31+G(d)
Charge = 0
Spin = Singlet
Solvation = None
E(RB3LYP) = -566.73563 Hartree
RMS Gradient Norm = 0.004465715 Hartree/Bohr
Dipole Moment = 3.9504631 Debye
Polarizability (?) = 170.321 a.u.

Opt Tab Data Section:
Maximum force = 0 Converged
RMS force = 0 Converged
Maximum displacement = 1.4e-05 Converged
RMS displacement = 2e-06 Converged
Predicted energy change = -2.398737e-14 Hartree

PAI-S0-R120 (*s-trans*; Φ_{CNNC} = 120°)

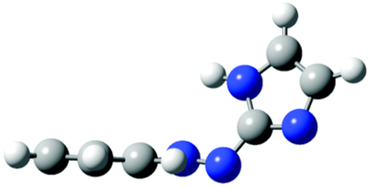

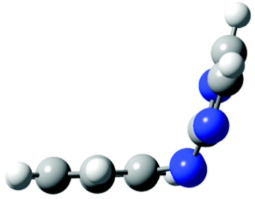

C
C,1,B1
C,1,B2,2,A1
C,1,B3,3,A2,2,D1,0
C,2,B4,1,A3,4,D2,0
C,2,B5,1,A4,4,D3,0
N,3,B6,1,A5,4,D4,0
N,7,B7,3,A6,1,D5,0
C,8,B8,7,A7,3,-120.00001448,0
N,9,B9,8,A8,7,D7,0
C,10,B10,9,A9,8,D8,0
C,11,B11,10,A10,9,D9,0
N,12,B12,11,A11,10,D10,0
H,13,B13,12,A12,11,D11,0
H,12,B14,11,A13,10,D12,0
H,1,B15,4,A14,5,D13,0
H,4,B16,1,A15,3,D14,0
H,5,B17,2,A16,1,D15,0
H,2,B18,1,A17,4,D16,0
H,6,B19,2,A18,1,D17,0
H,11,B20,10,A19,9,D18,0
Variables:
B1=2.7996749
B2=1.4145851
B3=1.39003579
B4=1.40138156
B5=1.39228665
B6=1.38758034
B7=1.29076396
B8=1.35770095
B9=1.33959225
B10=1.35580786
B11=1.39284898
B12=1.36386152
B13=1.01225576
B14=1.08068372
B15=1.08635846
B16=1.0869009
B17=1.0866881
B18=1.08718451
B19=1.08477967
B20=1.08192839
A1=60.08939756
A2=120.17478215
A3=59.86215627
A4=60.81703216
A5=117.09870662
A6=117.37569911
A7=115.27161101
A8=124.70770004
A9=105.44295208
A10=111.03905927
A11=105.32366524
A12=128.93607541
A13=132.00640267
A14=121.33659332
A15=119.79206739
A16=120.06987761
A17=179.36323246
A18=121.31691311
A19=121.54671572
D1=0.19932101
D2=-0.05831552
D3=179.77269
D4=-175.13881247
D5=-173.3297889
D7=-178.34468629
D8=183.58805344
D9=-0.36318075
D10=-0.04733351
D11=-179.1729543
D12=-183.18949764
D13=-179.77999638
D14=179.76489606
D15=-179.1946467
D16=-63.07581088
D17=178.69425706
D18=-181.54497139

Overview Tab Data Section:
Calculation Method = RB3LYP
Formula = C9H8N4
Basis Set = 6-31+G(d)
Charge = 0
Spin = Singlet
Solvation = None
E(RB3LYP) = -566.7062 Hartree
RMS Gradient Norm = 0.008659613 Hartree/Bohr
Dipole Moment = 4.6128357 Debye
Polarizability (?) = 182.48267 a.u.

Opt Tab Data Section:
Maximum force = 0 Converged
RMS force = 0 Converged
Maximum displacement = 0.000595 Converged
RMS displacement = 7.9e-05 Converged
Predicted energy change = -4.170573e-12 Hartree

PAI-S0-R90 (*s-trans*; Φ_{CNNC} = 90°)

```
C
C,1,B1
C,1,B2,2,A1
C,1,B3,3,A2,2,D1,0
C,4,B4,1,A3,3,D2,0
C,2,B5,1,A4,4,D3,0
N,3,B6,1,A5,4,D4,0
N,7,B7,3,A6,1,D5,0
C,8,B8,7,A7,3,-90.,0
N,9,B9,8,A8,7,D7,0
C,10,B10,9,A9,8,D8,0
C,11,B11,10,A10,9,D9,0
N,12,B12,11,A11,10,D10,0
H,13,B13,12,A12,11,D11,0
H,12,B14,11,A13,10,D12,0
H,1,B15,4,A14,5,D13,0
H,4,B16,1,A15,3,D14,0
H,5,B17,4,A16,1,D15,0
H,2,B18,1,A17,4,D16,0
H,6,B19,2,A18,1,D17,0
H,11,B20,10,A19,9,D18,0
     Variables:
B1=2.80606052
B2=1.42871934
B3=1.38358725
B4=1.40920081
B5=1.39245299
B6=1.35400639
B7=1.31387653
B8=1.32980906
B9=1.35549
B10=1.34248771
B11=1.40469705
B12=1.35510652
B13=1.01260996
B14=1.08084974
B15=1.08612297
B16=1.08722182
B17=1.08622765
B18=1.08758086
B19=1.084808
B20=1.0824304
A1=60.43134789
A2=120.25905961
A3=120.78228497
A4=61.11927089
A5=118.54247369
A6=121.93443234
A7=116.00038877
A8=125.56093695
A9=105.54166916
A10=111.39495208
A11=105.42798779
A12=128.71691012
A13=131.62884958
A14=121.43748192
A15=119.47185588
A16=120.20295981
A17=179.61810092
A18=120.9915831
A19=121.71002158
D1=0.70353908
D2=-0.42408379
D3=179.25196138
D4=186.10937722
D5=-178.131889
D7=182.22037113
D8=184.36325042
D9=-0.55548123
D10=-0.19597342
D11=-179.2759849
D12=175.62421504
D13=180.19777312
D14=-180.5486751
D15=-181.0196214
D16=-45.70802489
D17=179.24491304
D18=178.08575221
```

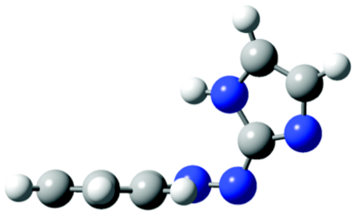

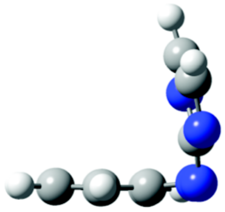

Overview Tab Data Section:
Calculation Method = RB3LYP
Formula = C9H8N4
Basis Set = 6-31+G(d)
Charge = 0
Spin = Singlet
Solvation = None
E(RB3LYP) = -566.66802 Hartree
RMS Gradient Norm = 0.010727983 Hartree/Bohr
Dipole Moment = 5.2734279 Debye
Polarizability (?) = 208.92833 a.u.

Opt Tab Data Section:
Maximum force = 1e-06 Converged
RMS force = 0 Converged
Maximum displacement = 0.000929 Converged
RMS displacement = 0.000124 Converged
Predicted energy change = -3.393017e-12 Hartree

PAI-S0-R60 (*s-trans*; Φ_{CNNC} = 60°)

C
C,1,B1
C,1,B2,2,A1
C,1,B3,3,A2,2,D1,0
C,4,B4,1,A3,3,D2,0
C,2,B5,1,A4,4,D3,0
N,3,B6,1,A5,4,D4,0
N,7,B7,3,A6,1,D5,0
C,8,B8,7,A7,3,-60.0000237,0
N,9,B9,8,A8,7,D7,0
C,10,B10,9,A9,8,D8,0
C,11,B11,10,A10,9,D9,0
N,12,B12,11,A11,10,D10,0
H,13,B13,12,A12,11,D11,0
H,12,B14,11,A13,10,D12,0
H,1,B15,4,A14,5,D13,0
H,4,B16,1,A15,3,D14,0
H,5,B17,4,A16,1,D15,0
H,2,B18,1,A17,4,D16,0
H,6,B19,2,A18,1,D17,0
H,11,B20,10,A19,9,D18,0
Variables:
B1=2.79651479
B2=1.41361075
B3=1.39174471
B4=1.40136367
B5=1.39237894
B6=1.3984411
B7=1.27737592
B8=1.37296937
B9=1.33626587
B10=1.35840247
B11=1.3880749
B12=1.36891185
B13=1.01127925
B14=1.08072438
B15=1.08635411
B16=1.08695135
B17=1.08637472
B18=1.08720446
B19=1.0858777
B20=1.08167579
A1=60.29454646
A2=120.29106263
A3=120.35722025
A4=60.75974356
A5=117.26994995
A6=123.71632544
A7=122.35673462
A8=122.74104123
A9=105.83948481
A10=110.87120109
A11=105.39004254
A12=126.65860625
A13=132.2035105
A14=121.18214464
A15=119.61551547
A16=120.2577359
A17=178.36865512
A18=120.38030634
A19=121.51853794
D1=1.39666642
D2=-1.50648279
D3=-178.54558695
D4=173.81562189
D5=167.61587031
D7=183.50158976
D8=-185.97193982
D9=-0.64387259
D10=1.08078804
D11=179.44517316
D12=183.9743406
D13=177.78994368
D14=179.54371023
D15=-178.46807964
D16=89.20314536
D17=-176.86441694
D18=180.78575374

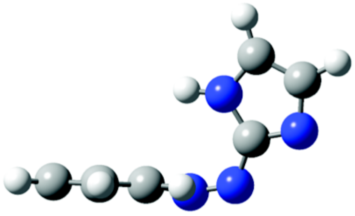

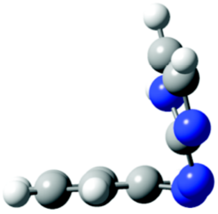

Overview Tab Data Section:
Calculation Method = RB3LYP
Formula = C9H8N4
Basis Set = 6-31+G(d)
Charge = 0
Spin = Singlet
Solvation = None
E(RB3LYP) = -566.69579 Hartree
RMS Gradient Norm = 0.008616289 Hartree/Bohr
Dipole Moment = 5.8501898 Debye
Polarizability (?) = 168.95367 a.u.

Opt Tab Data Section:
Maximum force = 0 Converged
RMS force = 0 Converged
Maximum displacement = 5e-06 Converged
RMS displacement = 1e-06 Converged
Predicted energy change = -1.484513e-13 Hartree

PAI-S0-R30 (*s-trans*; Φ_{CNNC} = 30°)

C
C,1,B1
C,1,B2,2,A1
C,1,B3,3,A2,2,D1,0
C,2,B4,1,A3,4,D2,0
C,2,B5,1,A4,4,D3,0
N,3,B6,1,A5,4,D4,0
N,7,B7,3,A6,1,D5,0
C,8,B8,7,A7,3,-30.00000445,0
N,9,B9,8,A8,7,D7,0
C,10,B10,9,A9,8,D8,0
C,11,B11,10,A10,9,D9,0
N,12,B12,11,A11,10,D10,0
H,13,B13,12,A12,11,D11,0
H,12,B14,11,A13,10,D12,0
H,1,B15,4,A14,5,D13,0
H,4,B16,1,A15,3,D14,0
H,5,B17,2,A16,1,D15,0
H,2,B18,1,A17,4,D16,0
H,6,B19,2,A18,1,D17,0
H,11,B20,10,A19,9,D18,0
Variables:
B1=2.8029939
B2=1.40894765
B3=1.39461496
B4=1.39951085
B5=1.39575507
B6=1.42290326
B7=1.26114718
B8=1.39473091
B9=1.3307143
B10=1.36114143
B11=1.38502632
B12=1.36915695
B13=1.01097452
B14=1.08075317
B15=1.08627275
B16=1.0870486
B17=1.08646581
B18=1.0869218
B19=1.0865726
B20=1.08151615
A1=59.96911804
A2=119.69001173
A3=60.01768256
A4=60.269444
A5=121.46682739
A6=123.6331157
A7=123.9152053
A8=120.92594968
A9=106.00632096
A10=110.61659287
A11=105.46085409
A12=126.34677448
A13=132.36369241
A14=120.54432125
A15=119.3873419
A16=120.20718383
A17=179.6955588
A18=121.04279955
A19=121.53858842
D1=0.12977694
D2=0.61411172
D3=-178.50968197
D4=-171.68448908
D5=-46.78145389
D7=175.88033046
D8=-186.43993489
D9=-1.21664268
D10=1.06912468
D11=177.83478845
D12=-177.44471509
D13=-179.08687206
D14=178.91840856
D15=-179.50203531
D16=-110.69930531
D17=178.4828723
D18=179.90137617

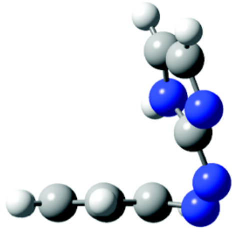

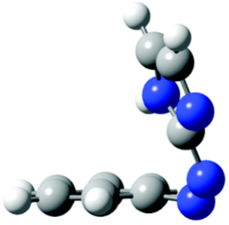

Overview Tab Data Section:
Calculation Method = RB3LYP
Formula = C9H8N4
Basis Set = 6-31+G(d)
Charge = 0
Spin = Singlet
Solvation = None
E(RB3LYP) = -566.71383 Hartree
RMS Gradient Norm = 0.003795634 Hartree/Bohr
Dipole Moment = 6.2103464 Debye
Polarizability (?) = 147.14967 a.u.

Opt Tab Data Section:
Maximum force = 0 Converged
RMS force = 0 Converged
Maximum displacement = 0.00037 Converged
RMS displacement = 4.9e-05 Converged
Predicted energy change = -5.838311e-13 Hartree

PAI-S0-R150 (*s-cis*; Φ_{CNNC} = 150°)

C
C,1,B1
C,1,B2,2,A1
C,1,B3,3,A2,2,D1,0
C,4,B4,1,A3,3,D2,0
C,2,B5,1,A4,4,D3,0
N,3,B6,1,A5,4,D4,0
N,7,B7,3,A6,1,D5,0
C,8,B8,7,A7,3,-150.,0
N,9,B9,8,A8,7,D7,0
C,10,B10,9,A9,8,D8,0
C,11,B11,10,A10,9,D9,0
N,12,B12,11,A11,10,D10,0
H,13,B13,12,A12,11,D11,0
H,12,B14,11,A13,10,D12,0
H,1,B15,4,A14,5,D13,0
H,4,B16,1,A15,3,D14,0
H,5,B17,4,A16,1,D15,0
H,2,B18,1,A17,4,D16,0
H,6,B19,2,A18,1,D17,0
H,11,B20,10,A19,9,D18,0
Variables:
B1=2.79659806
B2=1.40718742
B3=1.39371211
B4=1.3996254
B5=1.39221848
B6=1.40556944
B7=1.26902379
B8=1.37971818
B9=1.32971315
B10=1.36342313
B11=1.38699704
B12=1.3679731
B13=1.01136886
B14=1.08011042
B15=1.08610247
B16=1.08676961
B17=1.08695748
B18=1.08724943
B19=1.08461659
B20=1.08204007
A1=59.99586047
A2=120.1752144
A3=119.97132892
A4=60.61772118
A5=115.73712417
A6=115.63905704
A7=115.70580432
A8=131.39516368
A9=105.33215613
A10=111.07914319
A11=105.00031091
A12=127.79210677
A13=132.37953676
A14=121.48556915
A15=119.90632325
A16=120.06931992
A17=179.16200066
A18=121.16251474
A19=121.34611826
D1=-0.50265016
D2=0.49975037
D3=-180.31025053
D4=-177.54061185
D5=188.78900109
D7=10.4543732
D8=-183.89650885
D9=0.1984374
D10=-0.32395962
D11=-179.47404489
D12=180.8811587
D13=180.86157206
D14=180.04895801
D15=-180.79883479
D16=-68.95322666
D17=-181.4200534
D18=181.07081489

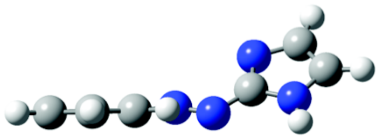

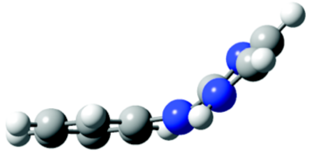

Overview Tab Data Section:
Calculation Method = RB3LYP
Formula = C9H8N4
Basis Set = 6-31+G(d)
Charge = 0
Spin = Singlet
Solvation = None
E(RB3LYP) = -566.73141 Hartree
RMS Gradient Norm = 0.004359353 Hartree/Bohr
Dipole Moment = 3.4234662 Debye
Polarizability (?) = 172.13067 a.u.

Opt Tab Data Section:
Maximum force = 1.3e-05 Converged
RMS force = 3e-06 Converged
Maximum displacement = 0.001693 Converged
RMS displacement = 0.000227 Converged
Predicted energy change = -8.318766e-10 Hartree

PAI-S0-R120 (*s-cis*; Φ_{CNNC} = 120°)

C
C,1,B1
C,1,B2,2,A1
C,1,B3,3,A2,2,D1,0
C,4,B4,1,A3,3,D2,0
C,2,B5,1,A4,4,D3,0
N,3,B6,1,A5,4,D4,0
N,7,B7,3,A6,1,D5,0
C,8,B8,7,A7,3,-120.,0
N,9,B9,8,A8,7,D7,0
C,10,B10,9,A9,8,D8,0
C,11,B11,10,A10,9,D9,0
N,12,B12,11,A11,10,D10,0
H,13,B13,12,A12,11,D11,0
H,12,B14,11,A13,10,D12,0
H,1,B15,4,A14,5,D13,0
H,4,B16,1,A15,3,D14,0
H,5,B17,4,A16,1,D15,0
H,2,B18,1,A17,4,D16,0
H,6,B19,2,A18,1,D17,0
H,11,B20,10,A19,9,D18,0
Variables:
B1=2.80088924
B2=1.41541841
B3=1.38921764
B4=1.40324937
B5=1.39172071
B6=1.38196196
B7=1.2873612
B8=1.35876992
B9=1.33796544
B10=1.36056431
B11=1.3890139
B12=1.36713477
B13=1.01120649
B14=1.08024014
B15=1.08594361
B16=1.08683116
B17=1.08679049
B18=1.08726039
B19=1.08439346
B20=1.08213041
A1=60.06878762
A2=120.17881262
A3=120.16262761
A4=60.81298653
A5=116.83473882
A6=117.46927032
A7=116.80349876
A8=130.13197244
A9=105.33368382
A10=111.21611702
A11=105.1592534
A12=127.76770039
A13=132.21673879
A14=121.50584368
A15=119.84580535
A16=120.04100356
A17=178.91547505
A18=121.21348075
A19=121.36127767
D1=-0.24936368
D2=0.2696182
D3=-180.43219854
D4=-175.26785715
D5=188.44950515
D7=13.49242539
D8=-186.44279184
D9=0.64917657
D10=-0.54681306
D11=-178.92705047
D12=181.5912947
D13=181.03472985
D14=179.74418438
D15=-181.1705028
D16=-68.54369525
D17=-182.20658732
D18=182.05732829

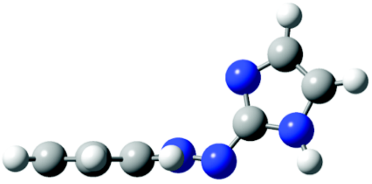

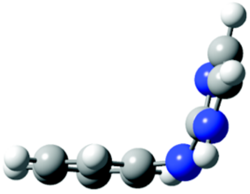

Overview Tab Data Section:
Calculation Method = RB3LYP
Formula = C9H8N4
Basis Set = 6-31+G(d)
Charge = 0
Spin = Singlet
Solvation = None
E(RB3LYP) = -566.70303 Hartree
RMS Gradient Norm = 0.008412634 Hartree/Bohr
Dipole Moment = 3.2918398 Debye
Polarizability (?) = 187.07533 a.u.

Opt Tab Data Section:
Maximum force = 2e-06 Converged
RMS force = 0 Converged
Maximum displacement = 0.000389 Converged
RMS displacement = 5.2e-05 Converged
Predicted energy change = -2.060237e-11 Hartree

PAI-S0-R90 (*s-cis*; Φ_{CNNC} = 90°)

C
C,1,B1
C,1,B2,2,A1
C,1,B3,3,A2,2,D1,0
C,4,B4,1,A3,3,D2,0
C,2,B5,1,A4,4,D3,0
N,3,B6,1,A5,4,D4,0
N,7,B7,3,A6,1,D5,0
C,8,B8,7,A7,3,-90.,0
N,9,B9,8,A8,7,D7,0
C,10,B10,9,A9,8,D8,0
C,11,B11,10,A10,9,D9,0
N,12,B12,11,A11,10,D10,0
H,13,B13,12,A12,11,D11,0
H,12,B14,11,A13,10,D12,0
H,1,B15,4,A14,5,D13,0
H,4,B16,1,A15,3,D14,0
H,5,B17,4,A16,1,D15,0
H,2,B18,1,A17,4,D16,0
H,6,B19,2,A18,1,D17,0
H,11,B20,10,A19,9,D18,0
Variables:
B1=2.8031573
B2=1.42257668
B3=1.38725032
B4=1.40424169
B5=1.38802966
B6=1.36324681
B7=1.29510259
B8=1.33901731
B9=1.34838431
B10=1.35568684
B11=1.39315735
B12=1.36562169
B13=1.01101655
B14=1.08006674
B15=1.08583919
B16=1.08697073
B17=1.08637738
B18=1.08740624
B19=1.08574158
B20=1.08246496
A1=60.27408944
A2=120.27423592
A3=120.50120905
A4=60.95843326
A5=117.16429276
A6=125.28161848
A7=126.52061094
A8=131.23810696
A9=105.46706752
A10=111.35970344
A11=105.21893238
A12=127.94730689
A13=132.07818351
A14=121.47329483
A15=119.60058261
A16=120.21038106
A17=178.6883832
A18=120.81945585
A19=121.50679996
D1=0.91111232
D2=-1.22830514
D3=-178.27651248
D4=-184.53900135
D5=182.3658952
D7=10.36466658
D8=-173.56037151
D9=-1.95833495
D10=1.46428409
D11=-185.18632844
D12=179.65355803
D13=178.52020675
D14=179.68699773
D15=-178.2109453
D16=73.09093344
D17=-177.42917411
D18=177.30389882

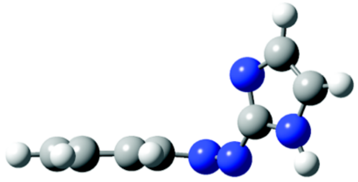

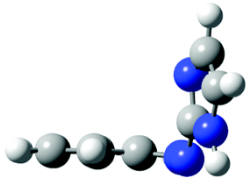

Overview Tab Data Section:
Calculation Method = RB3LYP
Formula = C9H8N4
Basis Set = 6-31+G(d)
Charge = 0
Spin = Singlet
Solvation = None
E(RB3LYP) = -566.66999 Hartree
RMS Gradient Norm = 0.010263576 Hartree/Bohr
Dipole Moment = 3.0912364 Debye
Polarizability (?) = 225.73533 a.u.

Opt Tab Data Section:
Maximum force = 0 Converged
RMS force = 0 Converged
Maximum displacement = 6.5e-05 Converged
RMS displacement = 9e-06 Converged
Predicted energy change = -7.064381e-13 Hartree

PAI-S0-R60 (*s-cis*; Φ_{CNNC} = 60°)

C
C,1,B1
C,1,B2,2,A1
C,1,B3,3,A2,2,D1,0
C,4,B4,1,A3,3,D2,0
C,2,B5,1,A4,4,D3,0
N,3,B6,1,A5,4,D4,0
N,7,B7,3,A6,1,D5,0
C,8,B8,7,A7,3,-60.,0
N,9,B9,8,A8,7,D7,0
C,10,B10,9,A9,8,D8,0
C,11,B11,10,A10,9,D9,0
N,12,B12,11,A11,10,D10,0
H,13,B13,12,A12,11,D11,0
H,12,B14,11,A13,10,D12,0
H,1,B15,4,A14,5,D13,0
H,4,B16,1,A15,3,D14,0
H,5,B17,4,A16,1,D15,0
H,2,B18,1,A17,4,D16,0
H,6,B19,2,A18,1,D17,0
H,11,B20,10,A19,9,D18,0
Variables:
B1=2.79472476
B2=1.41217793
B3=1.39144374
B4=1.40052678
B5=1.39039156
B6=1.39570902
B7=1.27647125
B8=1.37376947
B9=1.33475682
B10=1.3618983
B11=1.38848062
B12=1.36733991
B13=1.01148819
B14=1.08000522
B15=1.08609547
B16=1.08687088
B17=1.08665383
B18=1.08719034
B19=1.08488586
B20=1.08212545
A1=60.20757108
A2=120.35622858
A3=120.13595867
A4=60.72149611
A5=116.06043221
A6=124.31040116
A7=123.83429635
A8=131.88864167
A9=105.48469823
A10=111.05009282
A11=105.08441492
A12=127.93616213
A13=132.25567276
A14=121.31732805
A15=119.77056166
A16=120.20741769
A17=178.89593825
A18=120.30666035
A19=121.42344859
D1=1.3882375
D2=-1.64241019
D3=-178.28629248
D4=-186.04068198
D5=173.11362667
D7=1.39251983
D8=-171.34704494
D9=-1.08447996
D10=0.67621835
D11=-184.89664143
D12=179.62206079
D13=178.00729475
D14=179.32747219
D15=-178.58040168
D16=81.70372307
D17=-178.44639024
D18=178.46470048

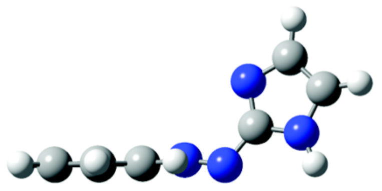

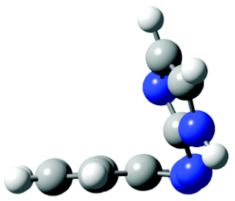

Overview Tab Data Section:
Calculation Method = RB3LYP
Formula = C9H8N4
Basis Set = 6-31+G(d)
Charge = 0
Spin = Singlet
Solvation = None
E(RB3LYP) = -566.6996 Hartree
RMS Gradient Norm = 0.008867912 Hartree/Bohr
Dipole Moment = 3.0953449 Debye
Polarizability (?) = 174.38567 a.u.

Opt Tab Data Section:
Maximum force = 0 Converged
RMS force = 0 Converged
Maximum displacement = 2.6e-05 Converged
RMS displacement = 4e-06 Converged
Predicted energy change = -5.31209e-14 Hartree

PAI-S0-R30 (*s-cis*; Φ_{CNNC} = 30°)

C
C,1,B1
C,1,B2,2,A1
C,1,B3,3,A2,2,D1,0
C,4,B4,1,A3,3,D2,0
C,2,B5,1,A4,4,D3,0
N,3,B6,1,A5,4,D4,0
N,7,B7,3,A6,1,D5,0
C,8,B8,7,A7,3,-30.,0
N,9,B9,8,A8,7,D7,0
C,10,B10,9,A9,8,D8,0
C,11,B11,10,A10,9,D9,0
N,12,B12,11,A11,10,D10,0
H,13,B13,12,A12,11,D11,0
H,12,B14,11,A13,10,D12,0
H,1,B15,4,A14,5,D13,0
H,4,B16,1,A15,3,D14,0
H,5,B17,4,A16,1,D15,0
H,2,B18,1,A17,4,D16,0
H,6,B19,2,A18,1,D17,0
H,11,B20,10,A19,9,D18,0
Variables:
B1=2.80251157
B2=1.40632792
B3=1.39230434
B4=1.40093849
B5=1.39416824
B6=1.41772543
B7=1.26142731
B8=1.39489647
B9=1.32915376
B10=1.36437425
B11=1.38648761
B12=1.36704278
B13=1.01178308
B14=1.08000221
B15=1.08532305
B16=1.08712359
B17=1.08675833
B18=1.08691489
B19=1.08629404
B20=1.08196259
A1=59.93365552
A2=119.70308843
A3=120.54475225
A4=60.16913332
A5=122.80104748
A6=124.23941368
A7=124.38013943
A8=132.91171136
A9=105.53061905
A10=110.90610739
A11=105.01867701
A12=127.9766356
A13=132.32171996
A14=120.28801537
A15=119.48361098
A16=120.06982617
A17=179.9024879
A18=121.12305
A19=121.35542369
D1=0.32430487
D2=-1.11719165
D3=181.98239725
D4=188.89860211
D5=-40.57850381
D7=-9.24245733
D8=-170.5510965
D9=-0.43381502
D10=0.5707325
D11=-183.91422584
D12=-179.62804188
D13=-179.94573545
D14=-180.93098035
D15=180.54712941
D16=207.04518491
D17=-181.0944407
D18=179.42144073

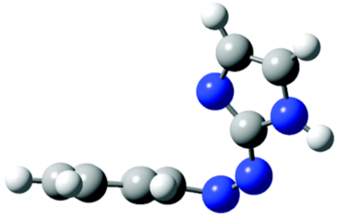

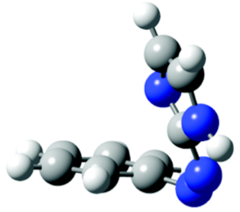

Overview Tab Data Section:
Calculation Method = RB3LYP
Formula = C9H8N4
Basis Set = 6-31+G(d)
Charge = 0
Spin = Singlet
Solvation = None
E(RB3LYP) = -566.71757 Hartree
RMS Gradient Norm = 0.003755123 Hartree/Bohr
Dipole Moment = 3.3627381 Debye
Polarizability (?) = 152.855 a.u.

Opt Tab Data Section:
Maximum force = 2e-06 Converged
RMS force = 0 Converged
Maximum displacement = 0.000952 Converged
RMS displacement = 0.000127 Converged
Predicted energy change = -2.524293e-11 Hartree

PAI-S0-I130 (*s-trans*; A_{NNC} = 130°)

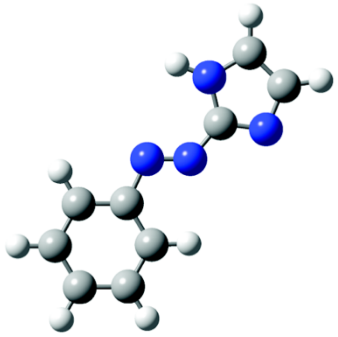

C
C,1,B1
C,1,B2,2,A1
C,1,B3,3,A2,2,D1,0
C,4,B4,1,A3,3,D2,0
C,2,B5,1,A4,4,D3,0
N,3,B6,1,A5,4,D4,0
N,7,B7,3,A6,1,D5,0
C,8,B8,7,130.7,3,D6,0
N,9,B9,8,A8,7,D7,0
C,10,B10,9,A9,8,D8,0
C,11,B11,10,A10,9,D9,0
N,12,B12,11,A11,10,D10,0
H,13,B13,12,A12,11,D11,0
H,12,B14,11,A13,10,D12,0
H,1,B15,4,A14,5,D13,0
H,4,B16,1,A15,3,D14,0
H,5,B17,4,A16,1,D15,0
H,2,B18,1,A17,4,D16,0
H,6,B19,2,A18,1,D17,0
H,11,B20,10,A19,9,D18,0
Variables:
B1=2.79454693
B2=1.40291952
B3=1.39587783
B4=1.39810538
B5=1.39204075
B6=1.41823544
B7=1.25482411
B8=1.35350005
B9=1.33147621
B10=1.36096037
B11=1.3884628
B12=1.36485944
B13=1.0118632
B14=1.08092271
B15=1.08635464
B16=1.08681532
B17=1.08703181
B18=1.08720016
B19=1.08458034
B20=1.0815162
A1=59.84933749
A2=120.07311422
A3=119.88246999
A4=60.61855013
A5=115.39272288
A6=114.72469404
A8=123.36634997
A9=105.63996973
A10=110.9663166
A11=105.28546652
A12=127.74691038
A13=132.01278345
A14=121.36753023
A15=119.92088262
A16=120.07509144
A17=179.73285203
A18=121.60041496
A19=121.41557926
D1=0.00000142
D2=-0.00000162
D3=180.00000099
D4=180.00000066
D5=-180.00000224
D6=-180.00000006
D7=-179.9999995
D8=180.00000011
D9=0.00000007
D10=-0.0000001
D11=179.99999992
D12=179.99999979
D13=179.99999927
D14=-180.00000077
D15=-179.99999845
D16=0.00118874
D17=-179.99999835
D18=180.00000001

Overview Tab Data Section:
Calculation Method = RB3LYP
Formula = C9H8N4
Basis Set = 6-31+G(d)
Charge = 0
Spin = Singlet
Solvation = None
E(RB3LYP) = -566.73408 Hartree
RMS Gradient Norm = 0.008552705 Hartree/Bohr
Dipole Moment = 3.1084119 Debye
Polarizability (?) = 168.86133 a.u.

Opt Tab Data Section:
Maximum force = 0 Converged
RMS force = 0 Converged
Maximum displacement = 2.9e-05 Converged
RMS displacement = 4e-06 Converged
Predicted energy change = -1.010124e-13 Hartree

PAI-S0-I145 (*s-trans*; A_{NNC} = 145°)

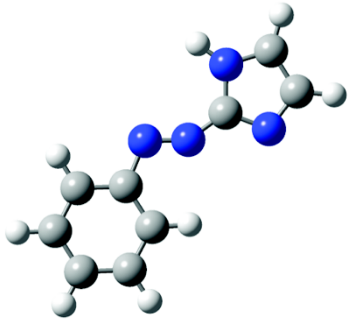

C
C,1,B1
C,1,B2,2,A1
C,1,B3,3,A2,2,D1,0
C,4,B4,1,A3,3,D2,0
C,2,B5,1,A4,4,D3,0
N,3,B6,1,A5,4,D4,0
N,7,B7,3,A6,1,D5,0
C,8,B8,7,145.7,3,D6,0
N,9,B9,8,A8,7,D7,0
C,10,B10,9,A9,8,D8,0
C,11,B11,10,A10,9,D9,0
N,12,B12,11,A11,10,D10,0
H,13,B13,12,A12,11,D11,0
H,12,B14,11,A13,10,D12,0
H,1,B15,4,A14,5,D13,0
H,4,B16,1,A15,3,D14,0
H,5,B17,4,A16,1,D15,0
H,2,B18,1,A17,4,D16,0
H,6,B19,2,A18,1,D17,0
H,11,B20,10,A19,9,D18,0
Variables:
B1=2.79525578
B2=1.40244764
B3=1.39594633
B4=1.39839793
B5=1.39227762
B6=1.42338626
B7=1.25272861
B8=1.33019268
B9=1.33901482
B10=1.356397
B11=1.39099157
B12=1.36009302
B13=1.01175655
B14=1.08128132
B15=1.08643245
B16=1.08689651
B17=1.08709956
B18=1.08730185
B19=1.08502373
B20=1.08166197
A1=59.77881385
A2=119.99624712
A3=119.88604286
A4=60.59505009
A5=115.23744309
A6=114.40038899
A8=123.56951752
A9=106.16503022
A10=111.06815495
A11=105.30301516
A12=127.1659989
A13=131.80455655
A14=121.47129285
A15=119.93630708
A16=120.06449355
A17=179.71468239
A18=121.79181414
A19=121.47191023
D1=0.00002518
D2=-0.00002947
D3=180.00001828
D4=180.0000123
D5=-180.00001125
D6=-179.99999973
D7=-179.99999885
D8=179.9999999
D9=0.00000006
D10=-0.00000016
D11=180.00000001
D12=179.99999965
D13=179.99998509
D14=-180.00001159
D15=-179.99996879
D16=0.02499459
D17=-179.99996945
D18=179.99999992

Overview Tab Data Section:
Calculation Method = RB3LYP
Formula = C9H8N4
Basis Set = 6-31+G(d)
Charge = 0
Spin = Singlet
Solvation = None
E(RB3LYP) = -566.71225 Hartree
RMS Gradient Norm = 0.010715132 Hartree/Bohr
Dipole Moment = 2.9675735 Debye
Polarizability (?) = 170.81967 a.u.

Opt Tab Data Section:
Maximum force = 1e-06 Converged
RMS force = 0 Converged
Maximum displacement = 0.000428 Converged
RMS displacement = 5.7e-05 Converged
Predicted energy change = -6.618323e-13 Hartree

PAI-S0-I160 (*s*-*trans*; A_{NNC} = 160°)

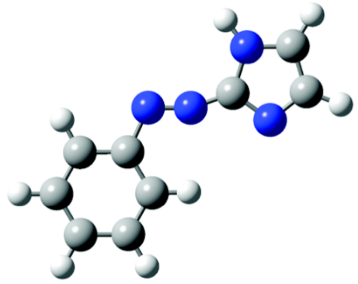

C
C,1,B1
C,1,B2,2,A1
C,1,B3,3,A2,2,D1,0
C,4,B4,1,A3,3,D2,0
C,2,B5,1,A4,4,D3,0
N,3,B6,1,A5,4,D4,0
N,7,B7,3,A6,1,D5,0
C,8,B8,7,160.7,3,D6,0
N,9,B9,8,A8,7,D7,0
C,10,B10,9,A9,8,D8,0
C,11,B11,10,A10,9,D9,0
N,12,B12,11,A11,10,D10,0
H,13,B13,12,A12,11,D11,0
H,12,B14,11,A13,10,D12,0
H,1,B15,4,A14,5,D13,0
H,4,B16,1,A15,3,D14,0
H,5,B17,4,A16,1,D15,0
H,2,B18,1,A17,4,D16,0
H,6,B19,2,A18,1,D17,0
H,11,B20,10,A19,9,D18,0
Variables:
B1=2.79621551
B2=1.40216882
B3=1.3958545
B4=1.39882113
B5=1.39262203
B6=1.42932364
B7=1.2544258
B8=1.30760826
B9=1.34902606
B10=1.35152943
B11=1.39448173
B12=1.35477055
B13=1.0118772
B14=1.08153465
B15=1.08659461
B16=1.08697502
B17=1.08713188
B18=1.08736158
B19=1.08590798
B20=1.08198607
A1=59.73690713
A2=119.94614912
A3=119.88932665
A4=60.5592028
A5=115.2275619
A6=114.5683518
A8=125.42990778
A9=106.69789281
A10=111.09300602
A11=105.37150578
A12=127.09343393
A13=131.55537544
A14=121.54336851
A15=119.96507503
A16=120.04830328
A17=179.70367606
A18=121.75002989
A19=121.65950365
D1=0.00000068
D2=-0.00000082
D3=180.0000005
D4=180.00000066
D5=-179.99999885
D6=-180.00000005
D7=-180.00000133
D8=180.00000001
D9=-0.00000025
D10=0.00000034
D11=179.99999995
D12=180.00000048
D13=179.99999959
D14=-180.00000023
D15=-179.99999899
D16=0.00114049
D17=-179.99999909
D18=179.99999993

Overview Tab Data Section:
Calculation Method = RB3LYP
Formula = C9H8N4
Basis Set = 6-31+G(d)
Charge = 0
Spin = Singlet
Solvation = None
E(RB3LYP) = -566.69199 Hartree
RMS Gradient Norm = 0.007743087 Hartree/Bohr
Dipole Moment = 2.9908483 Debye
Polarizability (?) = 171.84933 a.u.

Opt Tab Data Section:
Maximum force = 0 Converged
RMS force = 0 Converged
Maximum displacement = 9e-06 Converged
RMS displacement = 1e-06 Converged
Predicted energy change = -2.637423e-13 Hartree

PAI-S0-I175 (*s*-*trans*; A_{NNC} = 175°)

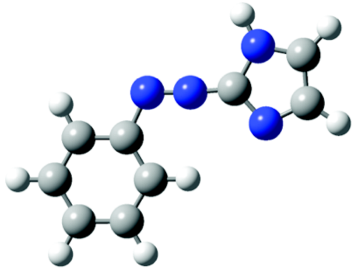

C
C,1,B1
C,1,B2,2,A1
C,1,B3,3,A2,2,D1,0
C,4,B4,1,A3,3,D2,0
C,2,B5,1,A4,4,D3,0
N,3,B6,1,A5,4,D4,0
N,7,B7,3,A6,1,D5,0
C,8,B8,7,175.7,3,D6,0
N,9,B9,8,A8,7,D7,0
C,10,B10,9,A9,8,D8,0
C,11,B11,10,A10,9,D9,0
N,12,B12,11,A11,10,D10,0
H,13,B13,12,A12,11,D11,0
H,12,B14,11,A13,10,D12,0
H,1,B15,4,A14,5,D13,0
H,4,B16,1,A15,3,D14,0
H,5,B17,4,A16,1,D15,0
H,2,B18,1,A17,4,D16,0
H,6,B19,2,A18,1,D17,0
H,11,B20,10,A19,9,D18,0
Variables:
B1=2.79670744
B2=1.40230323
B3=1.39553035
B4=1.39927027
B5=1.39319692
B6=1.43459493
B7=1.25680864
B8=1.29750697
B9=1.35621993
B10=1.34895349
B11=1.39679418
B12=1.3520072
B13=1.01204818
B14=1.08143102
B15=1.08675165
B16=1.08702466
B17=1.08713992
B18=1.08737409
B19=1.08675752
B20=1.08232739
A1=59.74557223
A2=119.95680845
A3=119.87632508
A4=60.55734448
A5=115.25487165
A6=115.36955402
A8=128.13257417
A9=106.9080996
A10=111.01956111
A11=105.41130576
A12=127.48227019
A13=131.46063419
A14=121.56690384
A15=120.00214735
A16=120.03032887
A17=179.7067092
A18=121.41509433
A19=121.87384145
D1=0.00000202
D2=-0.00000225
D3=180.00000044
D4=180.00000895
D5=-179.99997688
D6=-179.99999737
D7=-180.00003622
D8=180.00000352
D9=-0.00000512
D10=0.00000719
D11=179.99999925
D12=180.00001046
D13=180.00000087
D14=-180.00000065
D15=-179.99999666
D16=0.00443423
D17=-179.99999725
D18=179.99999808

Overview Tab Data Section:
Calculation Method = RB3LYP
Formula = C9H8N4
Basis Set = 6-31+G(d)
Charge = 0
Spin = Singlet
Solvation = None
E(RB3LYP) = -566.68258 Hartree
RMS Gradient Norm = 0.001045881 Hartree/Bohr
Dipole Moment = 3.0112058 Debye
Polarizability (?) = 170.53467 a.u.

Opt Tab Data Section:
Maximum force = 0 Converged
RMS force = 0 Converged
Maximum displacement = 6.6e-05 Converged
RMS displacement = 9e-06 Converged
Predicted energy change = -5.303035e-14 Hartree

PAI-S0-I190 (*s-cis*; A_{NNC} = 190°)

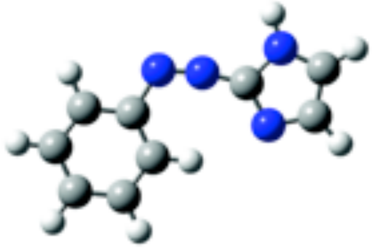

```
C
C,1,B1
C,1,B2,2,A1
C,1,B3,3,A2,2,D1,0
C,4,B4,1,A3,3,D2,0
C,2,B5,1,A4,4,D3,0
N,3,B6,1,A5,4,D4,0
N,7,B7,3,A6,1,D5,0
C,8,B8,7,169.99995051,3,D6,0
N,9,B9,8,A8,7,D7,0
C,10,B10,9,A9,8,D8,0
C,11,B11,10,A10,9,D9,0
N,12,B12,11,A11,10,D10,0
H,13,B13,12,A12,11,D11,0
H,12,B14,11,A13,10,D12,0
H,1,B15,4,A14,5,D13,0
H,4,B16,1,A15,3,D14,0
H,5,B17,4,A16,1,D15,0
H,2,B18,1,A17,4,D16,0
H,6,B19,2,A18,1,D17,0
H,11,B20,10,A19,9,D18,0
      Variables:
B1=2.7954343
B2=1.40342074
B3=1.39479329
B4=1.3996577
B5=1.39408954
B6=1.43642467
B7=1.25817105
B8=1.30765732
B9=1.35571777
B10=1.35063372
B11=1.39586199
B12=1.35332057
B13=1.01210169
B14=1.08098311
B15=1.08678274
B16=1.08701859
B17=1.08715147
B18=1.08736264
B19=1.08666567
B20=1.08245161
A1=59.83557327
A2=120.08204928
A3=119.82773874
A4=60.62733689
A5=114.86282805
A6=117.29738932
A8=131.03964412
A9=106.68961849
A10=110.88485587
A11=105.35977035
A12=128.12235485
A13=131.6184516
A14=121.54520543
A15=120.03989515
A16=120.02155609
A17=179.72829161
A18=120.90932535
A19=121.94015487
D1=0.00000729
D2=-0.00000819
D3=-180.00000188
D4=-179.99998424
D5=179.99980521
D6=-0.00024455
D7=0.00020732
D8=-180.00006616
D9=0.00008693
D10=-0.00009138
D11=-179.99992253
D12=-180.00007036
D13=179.99999897
D14=179.99999429
D15=-179.99999465
D16=0.01017785
D17=-179.99997601
D18=-179.99996341
```

Overview Tab Data Section:
Calculation Method = RB3LYP
Formula = C9H8N4
Basis Set = 6-31+G(d)
Charge = 0
Spin = Singlet
Solvation = None
E(RB3LYP) = -566.68711 Hartree
RMS Gradient Norm = 0.005342471 Hartree/Bohr
Dipole Moment = 2.9546738 Debye
Polarizability (?) = 167.07733 a.u.

Opt Tab Data Section:
Maximum force = 0.000192 Converged
RMS force = 4.2e-05 Converged
Maximum displacement = 0.000177 Converged
RMS displacement = 4.6e-05 Converged
Predicted energy change = -2.120232e-08 Hartree

PAI-S0-I205 (*s-cis*; A_{NNC} = 205°)

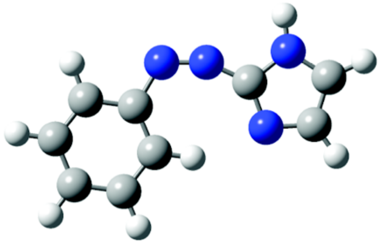

C
C,1,B1
C,1,B2,2,A1
C,1,B3,3,A2,2,D1,0
C,4,B4,1,A3,3,D2,0
C,2,B5,1,A4,4,D3,0
N,3,B6,1,A5,4,D4,0
N,7,B7,3,A6,1,D5,0
C,8,B8,7,155.00001985,3,D6,0
N,9,B9,8,A8,7,D7,0
C,10,B10,9,A9,8,D8,0
C,11,B11,10,A10,9,D9,0
N,12,B12,11,A11,10,D10,0
H,13,B13,12,A12,11,D11,0
H,12,B14,11,A13,10,D12,0
H,1,B15,4,A14,5,D13,0
H,4,B16,1,A15,3,D14,0
H,5,B17,4,A16,1,D15,0
H,2,B18,1,A17,4,D16,0
H,6,B19,2,A18,1,D17,0
H,11,B20,10,A19,9,D18,0
Variables:
B1=2.79119057
B2=1.40629264
B3=1.39345405
B4=1.39992657
B5=1.39522467
B6=1.43264878
B7=1.25900663
B8=1.3375745
B9=1.34843713
B10=1.35568862
B11=1.39214557
B12=1.35692723
B13=1.01210169
B14=1.08046074
B15=1.08657913
B16=1.08694613
B17=1.08717769
B18=1.08735482
B19=1.08509641
B20=1.08231078
A1=60.06654592
A2=120.39604825
A3=119.74170175
A4=60.76143308
A5=113.54486689
A6=121.24439969
A8=134.41381309
A9=106.32034289
A10=110.75102397
A11=105.24987403
A12=128.68358464
A13=131.8842576
A14=121.48475396
A15=120.06310882
A16=120.04823423
A17=179.79498793
A18=120.420066
A19=121.79952761
D1=0.00036256
D2=-0.00038267
D3=-180.
D4=180.
D5=179.99576447
D6=0.
D7=0.00454352
D8=179.99898628
D9=0.
D10=-0.00041324
D11=-180.
D12=179.99944581
D13=179.99970057
D14=180.
D15=-180.
D16=0.03634499
D17=-180.
D18=-179.99973734

Overview Tab Data Section:
Calculation Method = RB3LYP
Formula = C9H8N4
Basis Set = 6-31+G(d)
Charge = 0
Spin = Singlet
Solvation = None
E(RB3LYP) = -566.70196 Hartree
RMS Gradient Norm = 0.007929026 Hartree/Bohr
Dipole Moment = 2.8031482 Debye
Polarizability (?) = 162.642 a.u.

Opt Tab Data Section:
Maximum force = 0.000131 Converged
RMS force = 2.7e-05 Converged
Maximum displacement = 0.000629 Converged
RMS displacement = 9e-05 Converged
Predicted energy change = -1.112627e-08 Hartree

PAI-S0-I220 (*s-cis*; A_{NNC} = 220°)

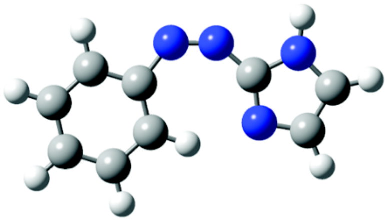

```
C
C,1,B1
C,1,B2,2,A1
C,1,B3,3,A2,2,D1,0
C,2,B4,1,A3,4,D2,0
C,2,B5,1,A4,4,D3,0
N,3,B6,1,A5,4,D4,0
N,7,B7,3,A6,1,D5,0
C,8,B8,7,140.,3,D6,0
N,9,B9,8,A8,7,D7,0
C,10,B10,9,A9,8,D8,0
C,11,B11,10,A10,9,D9,0
N,12,B12,11,A11,10,D10,0
H,13,B13,12,A12,11,D11,0
H,12,B14,11,A13,10,D12,0
H,1,B15,4,A14,5,D13,0
H,4,B16,1,A15,3,D14,0
H,5,B17,2,A16,1,D15,0
H,2,B18,1,A17,4,D16,0
H,6,B19,2,A18,1,D17,0
H,11,B20,10,A19,9,D18,0
     Variables:
B1=2.78439163
B2=1.41111061
B3=1.39181492
B4=1.39932151
B5=1.39611717
B6=1.42471536
B7=1.26094487
B8=1.37517454
B9=1.33972099
B10=1.36129356
B11=1.38755455
B12=1.36052529
B13=1.01205541
B14=1.08014779
B15=1.08609333
B16=1.08684896
B17=1.0872211
B18=1.087369
B19=1.08246029
B20=1.08204394
A1=60.46513158
A2=120.90602213
A3=60.08501677
A4=60.91622751
A5=111.44265727
A6=127.27463809
A8=137.25859096
A9=106.16036457
A10=110.67789176
A11=105.10747853
A12=128.68035417
A13=132.0898651
A14=121.37266086
A15=120.04630793
A16=120.05120413
A17=179.91071793
A18=120.19581443
A19=121.53862088
D1=0.00002063
D2=0.000002
D3=180.0000157
D4=180.00000801
D5=-180.00016123
D6=0.00000824
D7=0.00025002
D8=179.99991901
D9=-0.00000035
D10=-0.00000849
D11=-179.99995889
D12=-180.00001056
D13=179.99998627
D14=-180.00001291
D15=-180.00000928
D16=-0.00550114
D17=-179.99998833
D18=180.00000101
```

Overview Tab Data Section:
Calculation Method = RB3LYP
Formula = C9H8N4
Basis Set = 6-31+G(d)
Charge = 0
Spin = Singlet
Solvation = None
E(RB3LYP) = -566.71602 Hartree
RMS Gradient Norm = 0.004113548 Hartree/Bohr
Dipole Moment = 2.6341166 Debye
Polarizability (?) = 158.72433 a.u.

Opt Tab Data Section:
Maximum force = 3e-06 Converged
RMS force = 1e-06 Converged
Maximum displacement = 0.000125 Converged
RMS displacement = 1.7e-05 Converged
Predicted energy change = -9.869644e-12 Hartree

PAI-S0-I$_{Ph}$130 (*s-cis*; A_{NNPh} = 130°)

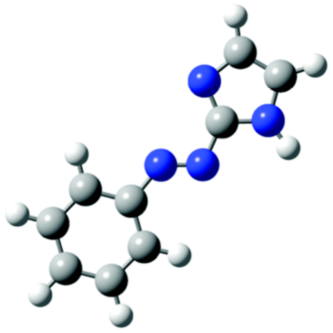

C
C,1,B1
C,1,B2,2,A1
C,1,B3,3,A2,2,D1,0
C,4,B4,1,A3,3,D2,0
C,2,B5,1,A4,4,D3,0
N,3,B6,1,A5,4,D4,0
N,7,B7,3,130.05,1,D5,0
C,8,B8,7,A7,3,D6,0
N,9,B9,8,A8,7,D7,0
C,10,B10,9,A9,8,D8,0
C,11,B11,10,A10,9,D9,0
N,12,B12,11,A11,10,D10,0
H,13,B13,12,A12,11,D11,0
H,12,B14,11,A13,10,D12,0
H,1,B15,4,A14,5,D13,0
H,4,B16,1,A15,3,D14,0
H,5,B17,4,A16,1,D15,0
H,2,B18,1,A17,4,D16,0
H,6,B19,2,A18,1,D17,0
H,11,B20,10,A19,9,D18,0
Variables:
B1=2.80632848
B2=1.40496655
B3=1.39494056
B4=1.39924384
B5=1.39149185
B6=1.3883053
B7=1.25270753
B8=1.38937119
B9=1.32638357
B10=1.36510519
B11=1.38601702
B12=1.36878512
B13=1.01145819
B14=1.08006132
B15=1.08552781
B16=1.08667149
B17=1.0870605
B18=1.08723614
B19=1.08629279
B20=1.08211189
A1=59.79718518
A2=119.79149444
A3=120.11961075
A4=60.3994346
A5=115.29670505
A7=114.93867212
A8=132.31975764
A9=105.24312404
A10=111.07034067
A11=104.90050897
A12=128.00233137
A13=132.47358582
A14=122.05729536
A15=119.82599501
A16=119.90765176
A17=179.51608589
A18=121.18777144
A19=121.31628665
D1=-0.00029395
D2=0.00036317
D3=179.9997596
D4=179.99994685
D5=-179.99956865
D6=-179.9999103
D7=0.00009093
D8=-180.00000688
D9=-0.00000069
D10=-0.00000231
D11=179.99998593
D12=-180.00000129
D13=-179.99975333
D14=-179.99988325
D15=179.99957114
D16=-0.23209922
D17=179.99954097
D18=-179.99999813

Overview Tab Data Section:
Calculation Method = RB3LYP
Formula = C9H8N4
Basis Set = 6-31+G(d)
Charge = 0
Spin = Singlet
Solvation = None
E(RB3LYP) = -566.73127 Hartree
RMS Gradient Norm = 0.008525136 Hartree/Bohr
Dipole Moment = 2.9093077 Debye
Polarizability (?) = 0 a.u.

Opt Tab Data Section:
Maximum force = 1.7e-05 Converged
RMS force = 3e-06 Converged
Maximum displacement = 0.000787 Converged
RMS displacement = 0.000105 Converged
Predicted energy change = -7.662018e-11 Hartree

PAI-S0-I$_{Ph}$145 (*s-cis*; A_{NNPh} = 145°)

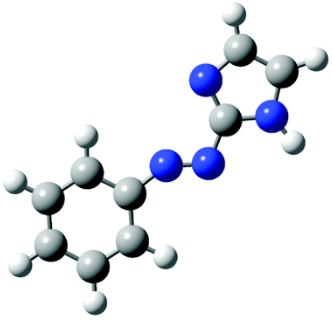

C
C,1,B1
C,1,B2,2,A1
C,1,B3,3,A2,2,D1,0
C,4,B4,1,A3,3,D2,0
C,2,B5,1,A4,4,D3,0
N,3,B6,1,A5,4,D4,0
N,7,B7,3,145.,1,D5,0
C,8,B8,7,A7,3,D6,0
N,9,B9,8,A8,7,D7,0
C,10,B10,9,A9,8,D8,0
C,11,B11,10,A10,9,D9,0
N,12,B12,11,A11,10,D10,0
H,13,B13,12,A12,11,D11,0
H,12,B14,11,A13,10,D12,0
H,1,B15,4,A14,5,D13,0
H,4,B16,1,A15,3,D14,0
H,5,B17,4,A16,1,D15,0
H,2,B18,1,A17,4,D16,0
H,6,B19,2,A18,1,D17,0
H,11,B20,10,A19,9,D18,0
Variables:
B1=2.8098722
B2=1.40902245
B3=1.39424383
B4=1.39974638
B5=1.39138095
B6=1.37048966
B7=1.2491284
B8=1.39548818
B9=1.32548928
B10=1.3661657
B11=1.38559604
B12=1.36952011
B13=1.01159
B14=1.08011077
B15=1.08516163
B16=1.08671778
B17=1.08711812
B18=1.08726869
B19=1.08661472
B20=1.08220634
A1=60.02034986
A2=119.93329166
A3=120.20764628
A4=60.34846221
A5=115.58463176
A7=114.45003628
A8=132.38978056
A9=105.17426942
A10=111.06876743
A11=104.87624451
A12=128.14504615
A13=132.48725722
A14=122.24868908
A15=119.77692289
A16=119.85503514
A17=179.46851515
A18=120.96095654
A19=121.32176735
D1=-0.00225712
D2=0.00253857
D3=179.9985554
D4=179.99889273
D5=-179.99828875
D6=180.01450332
D7=-0.00021766
D8=180.00062572
D9=-0.00011619
D10=-0.00040136
D11=179.99956087
D12=180.00105106
D13=-179.99894891
D14=-179.99895981
D15=179.9973545
D16=-2.30980579
D17=179.99707875
D18=180.00098624

Overview Tab Data Section:
Calculation Method = RB3LYP
Formula = C9H8N4
Basis Set = 6-31+G(d)
Charge = 0
Spin = Singlet
Solvation = None
E(RB3LYP) = -566.70786 Hartree
RMS Gradient Norm = 0.011931943 Hartree/Bohr
Dipole Moment = 2.1598972 Debye

Opt Tab Data Section:
Maximum force = 7.2e-05 Converged
RMS force = 2.2e-05 Converged
Maximum displacement = 0.000326 Converged
RMS displacement = 5.9e-05 Converged
Predicted energy change = -1.974273e-08 Hartree

PAI-S0-I$_{Ph}$160 (*s-cis*; A_{NNPh} = 160°)

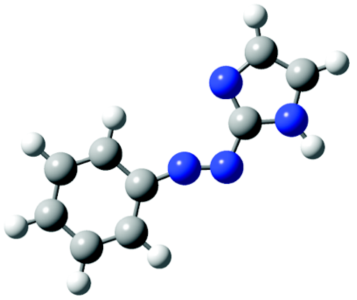

C
C,1,B1
C,1,B2,2,A1
C,1,B3,3,A2,2,D1,0
C,4,B4,1,A3,3,D2,0
C,2,B5,1,A4,4,D3,0
N,3,B6,1,A5,4,D4,0
N,7,B7,3,160.05,1,D5,0
C,8,B8,7,A7,3,D6,0
N,9,B9,8,A8,7,D7,0
C,10,B10,9,A9,8,D8,0
C,11,B11,10,A10,9,D9,0
N,12,B12,11,A11,10,D10,0
H,13,B13,12,A12,11,D11,0
H,12,B14,11,A13,10,D12,0
H,1,B15,4,A14,5,D13,0
H,4,B16,1,A15,3,D14,0
H,5,B17,4,A16,1,D15,0
H,2,B18,1,A17,4,D16,0
H,6,B19,2,A18,1,D17,0
H,11,B20,10,A19,9,D18,0
Variables:
B1=2.81004931
B2=1.41529027
B3=1.39369896
B4=1.40011728
B5=1.39127977
B6=1.35421798
B7=1.24894141
B8=1.40507467
B9=1.32499515
B10=1.36750961
B11=1.38502398
B12=1.37065033
B13=1.01173229
B14=1.08017798
B15=1.08530226
B16=1.08690735
B17=1.08720286
B18=1.0872883
B19=1.08649915
B20=1.08228085
A1=60.33657562
A2=120.27097676
A3=120.16696281
A4=60.37515174
A5=117.17666836
A7=114.29459196
A8=132.06453475
A9=105.14072044
A10=111.01845126
A11=104.88330828
A12=128.2171605
A13=132.46869141
A14=122.1636143
A15=119.84081134
A16=119.83571312
A17=179.43209592
A18=120.93234677
A19=121.37009679
D1=-0.03577412
D2=0.04336234
D3=179.97137187
D4=179.97656205
D5=-180.05555675
D6=-179.91800685
D7=0.00699763
D8=-179.99760889
D9=0.00040506
D10=-0.0000166
D11=180.00142106
D12=-180.00027039
D13=-179.97441556
D14=-179.98618028
D15=179.94960684
D16=-21.91228815
D17=179.94845579
D18=-179.99958911

Overview Tab Data Section:
Calculation Method = RB3LYP
Formula = C9H8N4
Basis Set = 6-31+G(d)
Charge = 0
Spin = Singlet
Solvation = None
E(RB3LYP) = -566.68396 Hartree
RMS Gradient Norm = 0.009345356 Hartree/Bohr
Dipole Moment = 1.3692362 Debye

Opt Tab Data Section:
Maximum force = 0.000113 Converged
RMS force = 3.2e-05 Converged
Maximum displacement = 0.00164 Converged
RMS displacement = 0.000295 Converged
Predicted energy change = -7.48648e-08 Hartree

PAI-S0-I_{Ph}175 (*s-cis*; A_{NNPh} = 175°)

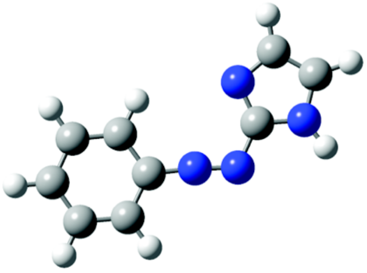

C
C,1,B1
C,1,B2,2,A1
C,1,B3,3,A2,2,D1,0
C,2,B4,1,A3,4,D2,0
C,2,B5,1,A4,4,D3,0
N,3,B6,1,A5,4,D4,0
N,7,B7,3,175.,1,D5,0
C,8,B8,7,A7,3,D6,0
N,9,B9,8,A8,7,D7,0
C,10,B10,9,A9,8,D8,0
C,11,B11,10,A10,9,D9,0
N,9,B12,8,A11,7,D10,0
H,13,B13,9,A12,8,D11,0
H,12,B14,11,A13,10,D12,0
H,1,B15,4,A14,5,D13,0
H,4,B16,1,A15,3,D14,0
H,5,B17,2,A16,1,D15,0
H,2,B18,1,A17,4,D16,0
H,6,B19,2,A18,1,D17,0
H,11,B20,10,A19,9,D18,0
Variables:
B1=2.80873478
B2=1.42113004
B3=1.39315737
B4=1.40240533
B5=1.39142669
B6=1.34627941
B7=1.24984345
B8=1.41672327
B9=1.32530313
B10=1.36908579
B11=1.38412793
B12=1.36871254
B13=1.01181078
B14=1.08020986
B15=1.08581037
B16=1.08717445
B17=1.08729306
B18=1.08718455
B19=1.08611983
B20=1.08227581
A1=60.44125779
A2=120.46716329
A3=59.48398318
A4=60.48777163
A5=119.73502045
A7=114.66733397
A8=131.35849428
A9=105.16317521
A10=110.92103797
A11=117.13904118
A12=124.35258945
A13=132.41595033
A14=122.03632645
A15=120.0098037
A16=119.71925568
A17=179.4140565
A18=121.24325546
A19=121.44444204
D1=0.00581194
D2=0.00138148
D3=-179.99553643
D4=-179.9991139
D5=-180.03628042
D6=180.03921357
D7=-0.00067536
D8=-179.9995973
D9=0.00004908
D10=179.99995436
D11=-0.0007623
D12=-179.99994151
D13=179.99606584
D14=179.99734879
D15=179.99311833
D16=3.43813415
D17=-179.9919286
D18=-180.00001993

Overview Tab Data Section:
Calculation Method = RB3LYP
Formula = C9H8N4
Basis Set = 6-31+G(d)
Charge = 0
Spin = Singlet
Solvation = None
E(RB3LYP) = -566.67215 Hartree
RMS Gradient Norm = 0.001381069 Hartree/Bohr
Dipole Moment = 0.70646323 Debye

Opt Tab Data Section:
Maximum force = 7e-06 Converged
RMS force = 1e-06 Converged
Maximum displacement = 0.001156 Converged
RMS displacement = 0.000241 Converged
Predicted energy change = -9.768749e-10 Hartree

PAI-S0-I$_{Ph}$190 (*s-trans*; A_{NNPh} = 190°)

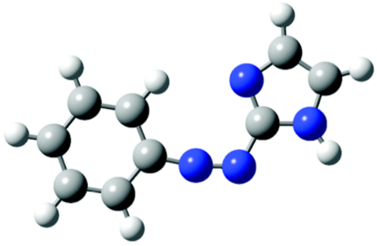

```
C
C,1,B1
C,1,B2,2,A1
C,1,B3,3,A2,2,D1,0
C,2,B4,1,A3,4,D2,0
C,2,B5,1,A4,4,D3,0
N,3,B6,1,A5,4,D4,0
N,7,B7,3,170.,1,D5,0
C,8,B8,7,A7,3,D6,0
N,9,B9,8,A8,7,D7,0
C,10,B10,9,A9,8,D8,0
C,11,B11,10,A10,9,D9,0
N,9,B12,8,A11,7,D10,0
H,13,B13,9,A12,8,D11,0
H,12,B14,11,A13,10,D12,0
H,1,B15,4,A14,5,D13,0
H,4,B16,1,A15,3,D14,0
H,5,B17,2,A16,1,D15,0
H,2,B18,1,A17,4,D16,0
H,6,B19,2,A18,1,D17,0
H,11,B20,10,A19,9,D18,0
     Variables:
B1=2.80830139
B2=1.42234952
B3=1.39310579
B4=1.40193602
B5=1.39176376
B6=1.35463864
B7=1.25100267
B8=1.42307569
B9=1.32664484
B10=1.37012715
B11=1.38324397
B12=1.36822302
B13=1.01181192
B14=1.08014718
B15=1.08576211
B16=1.08734802
B17=1.08734041
B18=1.08696445
B19=1.08551728
B20=1.08217256
A1=60.2486664
A2=120.27502678
A3=59.5046158
A4=60.54395082
A5=122.82686438
A7=116.3880188
A8=131.28307235
A9=105.27145783
A10=110.81214566
A11=117.36501161
A12=124.35943232
A13=132.34376043
A14=122.00960368
A15=120.05699224
A16=119.72045625
A17=179.49464109
A18=121.6993384
A19=121.49493565
D1=0.01111079
D2=0.00284161
D3=180.00895533
D4=180.00643467
D5=-0.01593597
D6=0.0135118
D7=-0.00214991
D8=-179.99975079
D9=0.00017401
D10=179.99929661
D11=-0.00933507
D12=-180.00095697
D13=-180.00802116
D14=179.99581541
D15=-180.01097994
D16=6.22507661
D17=180.01238334
D18=179.99971515
```

Overview Tab Data Section:
Calculation Method = RB3LYP
Formula = C9H8N4
Basis Set = 6-31+G(d)
Charge = 0
Spin = Singlet
Solvation = None
E(RB3LYP) = -566.67851 Hartree
RMS Gradient Norm = 0.006964474 Hartree/Bohr
Dipole Moment = 0.9671481 Debye

Opt Tab Data Section:
Maximum force = 5e-05 Converged
RMS force = 1.3e-05 Converged
Maximum displacement = 0.000351 Converged
RMS displacement = 7.6e-05 Converged
Predicted energy change = -7.510461e-09 Hartree

PAI-S0-I_{Ph}205 (*s-trans*; A_{NNPh} = 205°)

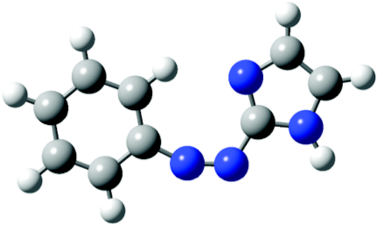

C
C,1,B1
C,1,B2,2,A1
C,1,B3,3,A2,2,D1,0
C,4,B4,1,A3,3,D2,0
C,2,B5,1,A4,4,D3,0
N,3,B6,1,A5,4,D4,0
N,7,B7,3,155.31431251,1,D5,0
C,8,B8,7,A7,3,D6,0
N,9,B9,8,A8,7,D7,0
C,10,B10,9,A9,8,D8,0
C,11,B11,10,A10,9,D9,0
N,12,B12,11,A11,10,D10,0
H,13,B13,12,A12,11,D11,0
H,12,B14,11,A13,10,D12,0
H,1,B15,4,A14,5,D13,0
H,4,B16,1,A15,3,D14,0
H,5,B17,4,A16,1,D15,0
H,2,B18,1,A17,4,D16,0
H,6,B19,2,A18,1,D17,0
H,11,B20,10,A19,9,D18,0
Variables:
B1=2.80993409
B2=1.41922895
B3=1.39386493
B4=1.40048609
B5=1.39182128
B6=1.37744837
B7=1.25378598
B8=1.4172505
B9=1.32867303
B10=1.36945934
B11=1.38311952
B12=1.37024799
B13=1.01183198
B14=1.08004796
B15=1.08447704
B16=1.08737682
B17=1.08732424
B18=1.08679313
B19=1.08513516
B20=1.0820333
A1=60.05335166
A2=119.88887113
A3=120.30243519
A4=60.39810422
A5=126.27832392
A7=120.48950738
A8=133.00064738
A9=105.50092081
A10=110.72879559
A11=105.04757497
A12=128.10896932
A13=132.2724799
A14=121.73744074
A15=119.81626004
A16=119.90528928
A17=179.7143916
A18=121.90291739
A19=121.4834675
D1=0.00458424
D2=-0.00531124
D3=-179.9960914
D4=-179.98623927
D5=-0.01837907
D6=-0.00787429
D7=0.00111927
D8=179.99819601
D9=-0.00038938
D10=0.
D11=-179.99769044
D12=-179.99901605
D13=179.99897905
D14=179.99739086
D15=-179.9985947
D16=0.07934309
D17=179.99916203
D18=179.99907975

Overview Tab Data Section:
Calculation Method = RB3LYP
Formula = C9H8N4
Basis Set = 6-31+G(d)
Charge = 0
Spin = Singlet
Solvation = None
E(RB3LYP) = -566.69701 Hartree
RMS Gradient Norm = 0.009762423 Hartree/Bohr
Dipole Moment = 1.6588827 Debye
Polarizability (?) = 159.82267 a.u.

Opt Tab Data Section:
Maximum force = 7.8e-05 Converged
RMS force = 1.6e-05 Converged
Maximum displacement = 0.001406 Converged
RMS displacement = 0.000199 Converged
Predicted energy change = -8.191551e-09 Hartree

PAI-S0-I_{Ph}220 (*s-trans*; A_{NNPh} = 220°)

C
C,1,B1
C,1,B2,2,A1
C,1,B3,3,A2,2,D1,0
C,4,B4,1,A3,3,D2,0
C,2,B5,1,A4,4,D3,0
N,3,B6,1,A5,4,D4,0
N,7,B7,3,140.,1,D5,0
C,8,B8,7,A7,3,D6,0
N,9,B9,8,A8,7,D7,0
C,10,B10,9,A9,8,D8,0
C,11,B11,10,A10,9,D9,0
N,12,B12,11,A11,10,D10,0
H,13,B13,12,A12,11,D11,0
H,12,B14,11,A13,10,D12,0
H,1,B15,4,A14,5,D13,0
H,4,B16,1,A15,3,D14,0
H,5,B17,4,A16,1,D15,0
H,2,B18,1,A17,4,D16,0
H,6,B19,2,A18,1,D17,0
H,11,B20,10,A19,9,D18,0
Variables:
B1=2.81314038
B2=1.41468001
B3=1.39533579
B4=1.39942819
B5=1.39137894
B6=1.40546088
B7=1.25890795
B8=1.40279248
B9=1.33215376
B10=1.36655283
B11=1.38411096
B12=1.36558157
B13=1.01192068
B14=1.0800267
B15=1.08230637
B16=1.08737127
B17=1.08727965
B18=1.08676386
B19=1.08535833
B20=1.08192772
A1=60.10700101
A2=119.59722932
A3=120.81635631
A4=60.02987542
A5=129.55638514
A7=127.25681102
A8=136.18528797
A9=105.89038508
A10=110.678984
A11=105.04477928
A12=128.29922386
A13=132.20257194
A14=120.92798686
A15=119.34630113
A16=120.02364655
A17=179.9728976
A18=121.62810741
A19=121.42638888
D1=-0.00009911
D2=0.00012626
D3=179.9999111
D4=179.99990309
D5=0.00017967
D6=0.0000301
D7=-0.0000833
D8=180.00000767
D9=0.00000118
D10=0.00000215
D11=-180.00000034
D12=180.00000081
D13=-179.9999116
D14=-179.99994952
D15=179.99987676
D16=180.99314772
D17=-180.00008893
D18=179.99999828

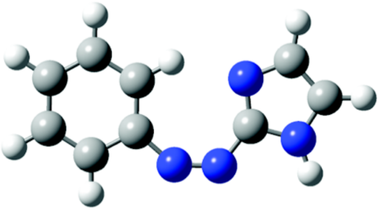

Overview Tab Data Section:
Calculation Method = RB3LYP
Formula = C9H8N4
Basis Set = 6-31+G(d)
Charge = 0
Spin = Singlet
Solvation = None
E(RB3LYP) = -566.71489 Hartree
RMS Gradient Norm = 0.005230146 Hartree/Bohr
Dipole Moment = 2.2672032 Debye
Polarizability (?) = 0 a.u.

Opt Tab Data Section:
Maximum force = 1.9e-05 Converged
RMS force = 4e-06 Converged
Maximum displacement = 0.001246 Converged
RMS displacement = 0.000167 Converged
Predicted energy change = -2.084467e-10 Hartree

PAI-S1-I130 (*s-trans*; A_{NNC} = 130°)

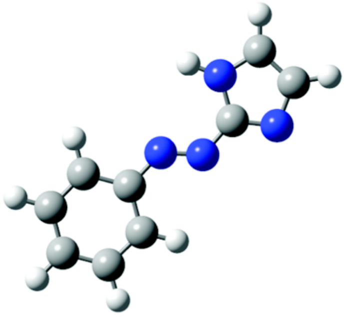

C
C,1,B1
C,1,B2,2,A1
C,1,B3,3,A2,2,D1,0
C,2,B4,1,A3,4,D2,0
C,2,B5,1,A4,4,D3,0
N,3,B6,1,A5,4,D4,0
N,7,B7,3,A6,1,D5,0
C,8,B8,7,128.40461061,3,D6,0
N,9,B9,8,A8,7,D7,0
C,10,B10,9,A9,8,D8,0
C,11,B11,10,A10,9,D9,0
N,12,B12,11,A11,10,D10,0
H,13,B13,12,A12,11,D11,0
H,12,B14,11,A13,10,D12,0
H,1,B15,4,A14,5,D13,0
H,4,B16,1,A15,3,D14,0
H,5,B17,2,A16,1,D15,0
H,2,B18,1,A17,4,D16,0
H,6,B19,2,A18,1,D17,0
H,11,B20,10,A19,9,D18,0
Variables:
B1=2.80278185
B2=1.41913958
B3=1.38852671
B4=1.40307462
B5=1.3886597
B6=1.35723713
B7=1.25738782
B8=1.34476777
B9=1.33559161
B10=1.36663667
B11=1.38063988
B12=1.38293468
B13=1.00988778
B14=1.07989029
B15=1.08577487
B16=1.08702608
B17=1.08585543
B18=1.08742277
B19=1.08505865
B20=1.081647
A1=59.99970054
A2=119.77145672
A3=60.05845152
A4=61.19381844
A5=118.88231079
A6=130.78713425
A8=124.77132219
A9=104.99965708
A10=111.51608498
A11=105.294596
A12=127.4612493
A13=132.60049792
A14=121.00649188
A15=119.17464677
A16=120.34021728
A17=179.92818422
A18=121.6135723
A19=120.97208569
D1=0.
D2=0.
D3=180.
D4=-180.
D5=-180.
D6=179.9968935
D7=179.99959727
D8=-180.
D9=0.
D10=0.
D11=-180.
D12=180.
D13=-180.
D14=-180.
D15=-180.
D16=0.02611141
D17=180.
D18=-180.

Overview Tab Data Section:
Calculation Method = RB3LYP TD-FC
Formula = C9H8N4
Basis Set = 6-31+G(d)
Charge = 0
Spin = Singlet
Solvation = None
E(TD-HF/TD-DFT) = -566.66405 Hartree
RMS Gradient Norm = 7.708e-06 Hartree/Bohr
Dipole Moment = 3.6682572 Debye
Polarizability (?) = 185.899 a.u.

Opt Tab Data Section:
Maximum force = 2.4e-05 Converged
RMS force = 6e-06 Converged
Maximum displacement = 0.001142 Converged
RMS displacement = 0.000154 Converged
Predicted energy change = -1.099213e-09 Hartree

PAI-S1-I145 (*s-trans*; A_{NNC} = 145°)

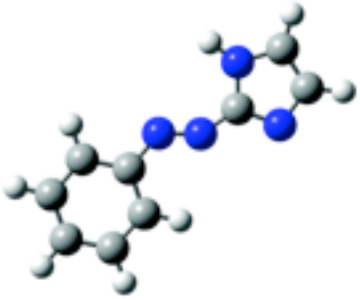

C
C,1,B1
C,1,B2,2,A1
C,1,B3,3,A2,2,D1,0
C,2,B4,1,A3,4,D2,0
C,2,B5,1,A4,4,D3,0
N,3,B6,1,A5,4,D4,0
N,7,B7,3,A6,1,D5,0
C,8,B8,7,145.,3,D6,0
N,9,B9,8,A8,7,D7,0
C,10,B10,9,A9,8,D8,0
C,11,B11,10,A10,9,D9,0
N,12,B12,11,A11,10,D10,0
H,13,B13,12,A12,11,D11,0
H,12,B14,11,A13,10,D12,0
H,1,B15,4,A14,5,D13,0
H,4,B16,1,A15,3,D14,0
H,5,B17,2,A16,1,D15,0
H,2,B18,1,A17,4,D16,0
H,6,B19,2,A18,1,D17,0
H,11,B20,10,A19,9,D18,0
Variables:
B1=2.80239287
B2=1.41662705
B3=1.38918746
B4=1.40263004
B5=1.38977351
B6=1.36603312
B7=1.24459344
B8=1.32586067
B9=1.34134875
B10=1.36533506
B11=1.38097777
B12=1.38263462
B13=1.00993882
B14=1.07986595
B15=1.08578082
B16=1.08703923
B17=1.08600609
B18=1.08736469
B19=1.08524826
B20=1.08173711
A1=59.93240874
A2=119.73367757
A3=60.04879075
A4=61.09042736
A5=118.40868559
A6=128.88667353
A8=124.50733336
A9=104.94146318
A10=111.75934458
A11=105.3484069
A12=127.25387652
A13=132.52516106
A14=121.11311581
A15=119.25037591
A16=120.30779903
A17=179.92946021
A18=121.4960413
A19=120.82500731
D1=-0.00000053
D2=0.00000307
D3=180.00000125
D4=-180.00000685
D5=-179.99997846
D6=179.99990651
D7=180.00003642
D8=-179.9999999
D9=0.00000118
D10=-0.00000054
D11=-179.99999845
D12=179.99999589
D13=-180.00000033
D14=-179.99999794
D15=-180.00000273
D16=-0.00002205
D17=179.99999692
D18=-179.99999899

Overview Tab Data Section:
Calculation Method = RB3LYP TD-FC
Formula = C9H8N4
Basis Set = 6-31+G(d)
Charge = 0
Spin = Singlet
Solvation = None
E(TD-HF/TD-DFT) = -566.65882 Hartree
RMS Gradient Norm = 0.003788161 Hartree/Bohr
Dipole Moment = 2.9721835 Debye
Polarizability (?) = 186.96267 a.u.

Opt Tab Data Section:
Maximum force = 0 Converged
RMS force = 0 Converged
Maximum displacement = 9.1e-05 Converged
RMS displacement = 1.2e-05 Converged
Predicted energy change = -5.47865e-14 Hartree

PAI-S1-I160 (*s-trans*; A_{NNC} = 160°)

C
C,1,B1
C,1,B2,2,A1
C,1,B3,3,A2,2,D1,0
C,2,B4,1,A3,4,D2,0
C,2,B5,1,A4,4,D3,0
N,3,B6,1,A5,4,D4,0
N,7,B7,3,A6,1,D5,0
C,8,B8,7,160.,3,D6,0
N,9,B9,8,A8,7,D7,0
C,10,B10,9,A9,8,D8,0
C,11,B11,10,A10,9,D9,0
N,12,B12,11,A11,10,D10,0
H,13,B13,12,A12,11,D11,0
H,12,B14,11,A13,10,D12,0
H,1,B15,4,A14,5,D13,0
H,4,B16,1,A15,3,D14,0
H,5,B17,2,A16,1,D15,0
H,2,B18,1,A17,4,D16,0
H,6,B19,2,A18,1,D17,0
H,11,B20,10,A19,9,D18,0
Variables:
B1=2.80256647
B2=1.4148523
B3=1.38960786
B4=1.40232759
B5=1.39058664
B6=1.37297839
B7=1.23682629
B8=1.31334056
B9=1.34697198
B10=1.36417478
B11=1.38133365
B12=1.38181793
B13=1.00984153
B14=1.07984033
B15=1.08577603
B16=1.08705358
B17=1.08608923
B18=1.08731959
B19=1.08559973
B20=1.08188147
A1=59.85329714
A2=119.66338811
A3=60.03458018
A4=61.04029812
A5=118.25292635
A6=128.28097403
A8=124.77377496
A9=104.98355905
A10=111.88133518
A11=105.39148681
A12=127.16790285
A13=132.45999031
A14=121.18568215
A15=119.29295249
A16=120.2860892
A17=179.93362896
A18=121.44149545
A19=120.76988899
D1=0.
D2=0.
D3=180.
D4=180.
D5=180.
D6=179.99966733
D7=-180.
D8=-180.
D9=0.
D10=0.
D11=-180.
D12=-180.
D13=180.
D14=-180.
D15=180.
D16=-0.02037101
D17=-180.
D18=-180.

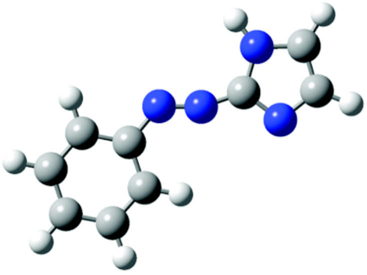

Overview Tab Data Section:
Calculation Method = RB3LYP TD-FC
Formula = C9H8N4
Basis Set = 6-31+G(d)
Charge = 0
Spin = Singlet
Solvation = None
E(TD-HF/TD-DFT) = -566.65022 Hartree
RMS Gradient Norm = 0.003889673 Hartree/Bohr
Dipole Moment = 2.583714 Debye
Polarizability (?) = 185.651 a.u.

Opt Tab Data Section:
Maximum force = 0.00031 Converged
RMS force = 5.8e-05 Converged
Maximum displacement = 0.000557 Converged
RMS displacement = 0.000143 Converged
Predicted energy change = -1.745043e-07 Hartree

PAI-S1-I175 (*s-trans*; A_{NNC} = 175°)

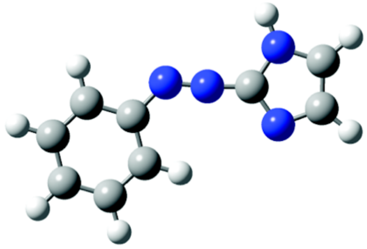

C
C,1,B1
C,1,B2,2,A1
C,1,B3,3,A2,2,D1,0
C,2,B4,1,A3,4,D2,0
C,2,B5,1,A4,4,D3,0
N,3,B6,1,A5,4,D4,0
N,7,B7,3,A6,1,D5,0
C,8,B8,7,175.,3,D6,0
N,9,B9,8,A8,7,D7,0
C,10,B10,9,A9,8,D8,0
C,11,B11,10,A10,9,D9,0
N,12,B12,11,A11,10,D10,0
H,13,B13,12,A12,11,D11,0
H,12,B14,11,A13,10,D12,0
H,1,B15,4,A14,5,D13,0
H,4,B16,1,A15,3,D14,0
H,5,B17,2,A16,1,D15,0
H,2,B18,1,A17,4,D16,0
H,6,B19,2,A18,1,D17,0
H,11,B20,10,A19,9,D18,0
Variables:
B1=2.80327768
B2=1.41421972
B3=1.38965591
B4=1.40223212
B5=1.39098341
B6=1.37624762
B7=1.23157059
B8=1.30803255
B9=1.3505386
B10=1.36406977
B11=1.38140619
B12=1.38147839
B13=1.00969322
B14=1.07975408
B15=1.08574464
B16=1.08707061
B17=1.08612209
B18=1.08728222
B19=1.08607813
B20=1.0820194
A1=59.77797048
A2=119.58483679
A3=60.01190082
A4=61.05481458
A5=118.38478434
A6=129.18268408
A8=125.71985183
A9=104.96967723
A10=111.89634598
A11=105.43655742
A12=127.23305646
A13=132.42069176
A14=121.22804431
A15=119.31210582
A16=120.27221278
A17=179.93096303
A18=121.40645092
A19=120.78204303
D1=0.
D2=0.
D3=180.
D4=180.
D5=180.
D6=179.99913947
D7=-179.9991396
D8=-180.
D9=0.
D10=0.
D11=-180.
D12=-180.
D13=-180.
D14=-180.
D15=-180.
D16=-0.00204978
D17=180.
D18=-180.

Overview Tab Data Section:
Calculation Method = RB3LYP TD-FC
Formula = C9H8N4
Basis Set = 6-31+G(d)
Charge = 0
Spin = Singlet
Solvation = None
E(TD-HF/TD-DFT) = -566.64481 Hartree
RMS Gradient Norm = 0.001062674 Hartree/Bohr
Dipole Moment = 2.337404 Debye
Polarizability (?) = 183.20467 a.u.

Opt Tab Data Section:
Maximum force = 0.000316 Converged
RMS force = 6.9e-05 Converged
Maximum displacement = 0.000732 Converged
RMS displacement = 0.000122 Converged
Predicted energy change = -1.039208e-07 Hartree

PAI-S1-I190 (*s*-cis; A_{NNC} = 190°)

C
C,1,B1
C,1,B2,2,A1
C,1,B3,3,A2,2,D1,0
C,4,B4,1,A3,3,D2,0
C,2,B5,1,A4,4,D3,0
N,3,B6,1,A5,4,D4,0
N,7,B7,3,A6,1,D5,0
C,8,B8,7,170.,3,D6,0
N,9,B9,8,A8,7,D7,0
C,10,B10,9,A9,8,D8,0
C,11,B11,10,A10,9,D9,0
N,12,B12,11,A11,10,D10,0
H,13,B13,12,A12,11,D11,0
H,12,B14,11,A13,10,D12,0
H,1,B15,4,A14,5,D13,0
H,4,B16,1,A15,3,D14,0
H,5,B17,4,A16,1,D15,0
H,2,B18,1,A17,4,D16,0
H,6,B19,2,A18,1,D17,0
H,11,B20,10,A19,9,D18,0
Variables:
B1=2.80370142
B2=1.41498187
B3=1.38926442
B4=1.40389723
B5=1.3909421
B6=1.37463663
B7=1.22834251
B8=1.31289715
B9=1.35018536
B10=1.36538365
B11=1.38095096
B12=1.38184732
B13=1.00955946
B14=1.07962108
B15=1.08570242
B16=1.08708572
B17=1.08613476
B18=1.08728657
B19=1.08639527
B20=1.08204211
A1=59.73251749
A2=119.55172236
A3=120.73005167
A4=61.15630159
A5=118.5695035
A6=131.47045574
A8=126.7272644
A9=104.87015104
A10=111.82055902
A11=105.45709095
A12=127.35764598
A13=132.41452558
A14=121.24839516
A15=119.32272258
A16=120.27140859
A17=179.90505995
A18=121.42083321
A19=120.81247
D1=-0.00118964
D2=0.00131913
D3=-180.00076317
D4=-180.00097905
D5=179.99967859
D6=0.00148007
D7=0.00008624
D8=-180.00015116
D9=0.00036401
D10=-0.00049618
D11=-179.99976615
D12=-180.0003373
D13=180.00035221
D14=180.00054943
D15=-180.00144921
D16=-3.65736651
D17=-180.00150097
D18=-179.99978881

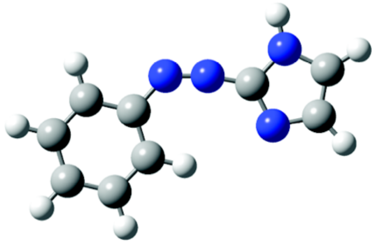

Overview Tab Data Section:
Calculation Method = RB3LYP TD-FC
Formula = C9H8N4
Basis Set = 6-31+G(d)
Charge = 0
Spin = Singlet
Solvation = None
E(TD-HF/TD-DFT) = -566.64614 Hartree
RMS Gradient Norm = 0.002105048 Hartree/Bohr
Dipole Moment = 2.1883735 Debye

Opt Tab Data Section:
Maximum force = 1e-05 Converged
RMS force = 2e-06 Converged
Maximum displacement = 5e-05 Converged
RMS displacement = 1.2e-05 Converged
Predicted energy change = -1.495069e-10 Hartree

PAI-S1-I205 (s-cis; A_{NNC} = 205°)

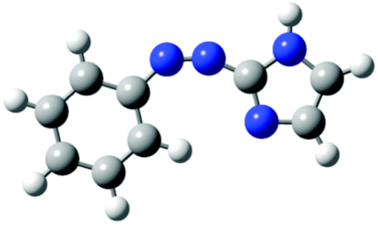

C
C,1,B1
C,1,B2,2,A1
C,1,B3,3,A2,2,D1,0
C,4,B4,1,A3,3,D2,0
C,2,B5,1,A4,4,D3,0
N,3,B6,1,A5,4,D4,0
N,7,B7,3,A6,1,D5,0
C,8,B8,7,155.00001985,3,D6,0
N,9,B9,8,A8,7,D7,0
C,10,B10,9,A9,8,D8,0
C,11,B11,10,A10,9,D9,0
N,12,B12,11,A11,10,D10,0
H,13,B13,12,A12,11,D11,0
H,12,B14,11,A13,10,D12,0
H,1,B15,4,A14,5,D13,0
H,4,B16,1,A15,3,D14,0
H,5,B17,4,A16,1,D15,0
H,2,B18,1,A17,4,D16,0
H,6,B19,2,A18,1,D17,0
H,11,B20,10,A19,9,D18,0
Variables:
B1=2.8030455
B2=1.41783266
B3=1.38826181
B4=1.40428386
B5=1.3903679
B6=1.36692369
B7=1.2266184
B8=1.32550945
B9=1.34654704
B10=1.36755477
B11=1.38007106
B12=1.38193443
B13=1.00954666
B14=1.07953406
B15=1.08563145
B16=1.08711138
B17=1.08611607
B18=1.0873661
B19=1.08594094
B20=1.08194027
A1=59.75595425
A2=119.60723546
A3=120.73635107
A4=61.3518296
A5=118.54135038
A6=136.56077414
A8=127.68726125
A9=104.82618208
A10=111.69002435
A11=105.4255987
A12=127.43259976
A13=132.40449581
A14=121.2437541
A15=119.30366564
A16=120.28692183
A17=179.86859695
A18=121.56749182
A19=120.82841804
D1=-0.00105701
D2=0.00101751
D3=-180.00031457
D4=180.00094974
D5=180.00198886
D6=-0.0214233
D7=0.01428742
D8=179.99704427
D9=-0.00078307
D10=0.00100678
D11=-179.99750932
D12=180.00028317
D13=180.00034952
D14=180.00030276
D15=-180.00128743
D16=-2.69927748
D17=-180.00231204
D18=-180.00060678

Overview Tab Data Section:
Calculation Method = RB3LYP TD-FC
Formula = C9H8N4
Basis Set = 6-31+G(d)
Charge = 0
Spin = Singlet
Solvation = None
E(TD-HF/TD-DFT) = -566.65154 Hartree
RMS Gradient Norm = 0.002598623 Hartree/Bohr
Dipole Moment = 2.1302946 Debye

Opt Tab Data Section:
Maximum force = 9e-06 Converged
RMS force = 2e-06 Converged
Maximum displacement = 0.000518 Converged
RMS displacement = 7.1e-05 Converged
Predicted energy change = -2.683891e-10 Hartree

PAI-S1-I220 (*s*-cis; A_{NNC} = 220°)

```
C
C,1,B1
C,1,B2,2,A1
C,1,B3,3,A2,2,D1,0
C,2,B4,1,A3,4,D2,0
C,2,B5,1,A4,4,D3,0
N,3,B6,1,A5,4,D4,0
N,7,B7,3,A6,1,D5,0
C,8,B8,7,140.,3,D6,0
N,9,B9,8,A8,7,D7,0
C,10,B10,9,A9,8,D8,0
C,11,B11,10,A10,9,D9,0
N,12,B12,11,A11,10,D10,0
H,13,B13,12,A12,11,D11,0
H,12,B14,11,A13,10,D12,0
H,1,B15,4,A14,5,D13,0
H,4,B16,1,A15,3,D14,0
H,5,B17,2,A16,1,D15,0
H,2,B18,1,A17,4,D16,0
H,6,B19,2,A18,1,D17,0
H,11,B20,10,A19,9,D18,0
        Variables:
B1=2.80186482
B2=1.42288649
B3=1.38682382
B4=1.40367642
B5=1.38904725
B6=1.35435614
B7=1.22922971
B8=1.34157558
B9=1.3408569
B10=1.36987385
B11=1.37910803
B12=1.38102883
B13=1.00971122
B14=1.0795552
B15=1.08548272
B16=1.08716235
B17=1.08601348
B18=1.087491
B19=1.0845578
B20=1.0817821
A1=59.87369293
A2=119.73799491
A3=60.03317648
A4=61.58422419
A5=118.28856511
A6=145.35200294
A8=128.69893002
A9=104.92754037
A10=111.50821951
A11=105.34545694
A12=127.45093088
A13=132.39291487
A14=121.20988406
A15=119.21048879
A16=120.36088767
A17=179.84373342
A18=121.89949337
A19=120.86354107
D1=-0.00041129
D2=0.00001089
D3=179.99992532
D4=180.00209308
D5=-179.97904482
D6=-0.04108923
D7=0.01482111
D8=179.99639911
D9=0.00237839
D10=-0.00301624
D11=-179.99836673
D12=-180.00144363
D13=179.99947475
D14=-179.99954708
D15=-179.99980966
D16=-0.04637293
D17=-180.00198712
D18=180.00160373
```

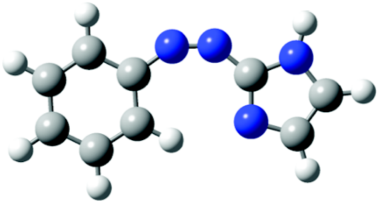

Overview Tab Data Section:
Calculation Method = RB3LYP TD-FC
Formula = C9H8N4
Basis Set = 6-31+G(d)
Charge = 0
Spin = Singlet
Solvation = None
E(TD-HF/TD-DFT) = -566.65501 Hartree
RMS Gradient Norm = 0.000403798 Hartree/Bohr
Dipole Moment = 2.027956 Debye
Polarizability (?) = 175.322 a.u.

Opt Tab Data Section:
Maximum force = 8e-06 Converged
RMS force = 2e-06 Converged
Maximum displacement = 0.000846 Converged
RMS displacement = 0.00016 Converged
Predicted energy change = -4.071649e-09 Hartree

PAI-S1-I_{Ph}130 (*s*-cis; A_{NNPh} = 130°)

C
C,1,B1
C,1,B2,2,A1
C,1,B3,3,A2,2,D1,0
C,4,B4,1,A3,3,D2,0
C,2,B5,1,A4,4,D3,0
N,3,B6,1,A5,4,D4,0
N,7,B7,3,130.05,1,D5,0
C,8,B8,7,A7,3,D6,0
N,9,B9,8,A8,7,D7,0
C,10,B10,9,A9,8,D8,0
C,11,B11,10,A10,9,D9,0
N,12,B12,11,A11,10,D10,0
H,13,B13,12,A12,11,D11,0
H,12,B14,11,A13,10,D12,0
H,1,B15,4,A14,5,D13,0
H,4,B16,1,A15,3,D14,0
H,5,B17,4,A16,1,D15,0
H,2,B18,1,A17,4,D16,0
H,6,B19,2,A18,1,D17,0
H,11,B20,10,A19,9,D18,0
Variables:
B1=2.80358228
B2=1.42060269
B3=1.38765865
B4=1.40435103
B5=1.38868597
B6=1.35382544
B7=1.25637695
B8=1.34743342
B9=1.33779623
B10=1.36928159
B11=1.38001367
B12=1.38073908
B13=1.00985936
B14=1.07962264
B15=1.08538689
B16=1.08701486
B17=1.08586533
B18=1.08759111
B19=1.08489208
B20=1.08194679
A1=60.02859664
A2=119.77879222
A3=120.94206473
A4=61.22008121
A5=118.70868438
A7=127.47388387
A8=128.58438853
A9=104.77115214
A10=111.59360339
A11=105.22630437
A12=127.25895204
A13=132.55224119
A14=121.24613478
A15=119.14572124
A16=120.41009877
A17=179.89981094
A18=121.5200478
A19=120.8515966
D1=0.01055795
D2=-0.01260826
D3=180.00794608
D4=180.00677556
D5=-179.99840593
D6=-180.00149536
D7=-0.00327025
D8=-179.99969919
D9=0.00006335
D10=0.00004806
D11=180.00052507
D12=-179.99999859
D13=-180.00613093
D14=-180.0049217
D15=180.01396797
D16=9.03560518
D17=180.01453606
D18=-180.00004607

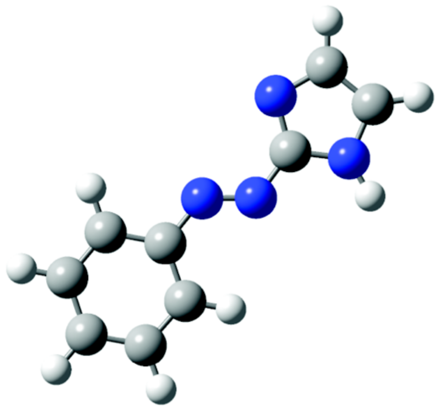

Overview Tab Data Section:
Calculation Method = RB3LYP TD-FC
Formula = C9H8N4
Basis Set = 6-31+G(d)
Charge = 0
Spin = Singlet
Solvation = None
E(TD-HF/TD-DFT) = -566.66437 Hartree
RMS Gradient Norm = 0.001010837 Hartree/Bohr
Dipole Moment = 3.4354723 Debye

Opt Tab Data Section:
Maximum force = 0.000314 Converged
RMS force = 7.2e-05 Converged
Maximum displacement = 0.000515 Converged
RMS displacement = 0.000116 Converged
Predicted energy change = -1.152124e-07 Hartree

PAI-S1-I_{Ph}145 (*s*-cis; A_{NNPh} = 145°)

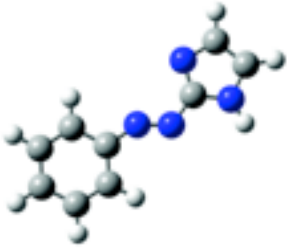

```
C
C,1,B1
C,1,B2,2,A1
C,1,B3,3,A2,2,D1,0
C,2,B4,1,A3,4,D2,0
C,2,B5,1,A4,4,D3,0
N,3,B6,1,A5,4,D4,0
N,7,B7,3,145.,1,D5,0
C,8,B8,7,A7,3,D6,0
N,9,B9,8,A8,7,D7,0
C,10,B10,9,A9,8,D8,0
C,11,B11,10,A10,9,D9,0
N,9,B12,8,A11,7,D10,0
H,13,B13,9,A12,8,D11,0
H,12,B14,11,A13,10,D12,0
H,1,B15,4,A14,5,D13,0
H,4,B16,1,A15,3,D14,0
H,5,B17,2,A16,1,D15,0
H,2,B18,1,A17,4,D16,0
H,6,B19,2,A18,1,D17,0
H,11,B20,10,A19,9,D18,0
        Variables:
B1=2.81003732
B2=1.42532353
B3=1.38628292
B4=1.4039013
B5=1.38739782
B6=1.33598363
B7=1.24686276
B8=1.3533392
B9=1.3351017
B10=1.37038555
B11=1.37971734
B12=1.37853505
B13=1.00996219
B14=1.07963895
B15=1.08495938
B16=1.08709509
B17=1.08574158
B18=1.08749373
B19=1.08528669
B20=1.08187638
A1=59.92913023
A2=119.52560198
A3=59.93658979
A4=61.3185928
A5=118.67966006
A7=125.22109185
A8=128.74513275
A9=104.79497676
A10=111.48389378
A11=119.64861506
A12=125.71784785
A13=132.54562912
A14=121.54246626
A15=118.95465579
A16=120.35207419
A17=179.78333039
A18=121.50433442
A19=120.92733043
D1=-0.00004639
D2=0.00000581
D3=179.99999731
D4=179.99995865
D5=180.00003362
D6=179.99983071
D7=-0.00006841
D8=180.00000863
D9=0.00000101
D10=-180.00005901
D11=-0.00001372
D12=-179.99999654
D13=-180.00000424
D14=-179.99997422
D15=-179.99998208
D16=-0.02680112
D17=179.99998097
D18=-180.00000277
```

Overview Tab Data Section:
Calculation Method = RB3LYP TD-FC
Formula = C9H8N4
Basis Set = 6-31+G(d)
Charge = 0
Spin = Singlet
Solvation = None
E(TD-HF/TD-DFT) = -566.66151 Hartree
RMS Gradient Norm = 0.003162391 Hartree/Bohr
Dipole Moment = 2.5840274 Debye
Polarizability (?) = 187.17467 a.u.

Opt Tab Data Section:
Maximum force = 0 Converged
RMS force = 0 Converged
Maximum displacement = 0.000468 Converged
RMS displacement = 6.3e-05 Converged
Predicted energy change = -5.136427e-13 Hartree

PAI-S1-I$_{Ph}$160 (*s*-cis; A_{NNPh} = 160°)

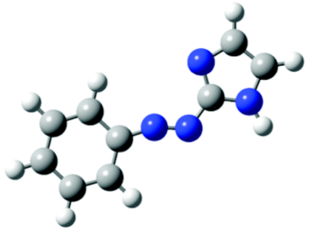

```
C
C,1,B1
C,1,B2,2,A1
C,1,B3,3,A2,2,D1,0
C,2,B4,1,A3,4,D2,0
C,2,B5,1,A4,4,D3,0
N,3,B6,1,A5,4,D4,0
N,7,B7,3,160.,1,D5,0
C,8,B8,7,A7,3,D6,0
N,9,B9,8,A8,7,D7,0
C,10,B10,9,A9,8,D8,0
C,11,B11,10,A10,9,D9,0
N,9,B12,8,A11,7,D10,0
H,13,B13,9,A12,8,D11,0
H,12,B14,11,A13,10,D12,0
H,1,B15,4,A14,5,D13,0
H,4,B16,1,A15,3,D14,0
H,5,B17,2,A16,1,D15,0
H,2,B18,1,A17,4,D16,0
H,6,B19,2,A18,1,D17,0
H,11,B20,10,A19,9,D18,0
     Variables:
B1=2.813098
B2=1.43038985
B3=1.38521765
B4=1.40431935
B5=1.38656886
B6=1.3254804
B7=1.24026563
B8=1.35884723
B9=1.33319801
B10=1.37178383
B11=1.37906335
B12=1.37664639
B13=1.00996946
B14=1.07963285
B15=1.08478822
B16=1.08719847
B17=1.08567069
B18=1.08742522
B19=1.0853443
B20=1.08180548
A1=59.94253388
A2=119.45199511
A3=59.8902486
A4=61.39379786
A5=118.75216789
A7=124.2050905
A8=128.54578121
A9=104.77755527
A10=111.40642506
A11=119.72326931
A12=125.70035626
A13=132.54304781
A14=121.74552962
A15=118.81678369
A16=120.37271276
A17=179.72838501
A18=121.41093444
A19=120.9702042
D1=0.
D2=0.
D3=-180.
D4=-180.
D5=-180.
D6=180.
D7=0.
D8=-180.
D9=0.
D10=-180.
D11=0.
D12=180.
D13=-180.
D14=180.
D15=180.
D16=0.055621
D17=-180.
D18=-180.
```

Overview Tab Data Section:
Calculation Method = RB3LYP TD-FC
Formula = C9H8N4
Basis Set = 6-31+G(d)
Charge = 0
Spin = Singlet
Solvation = None
E(TD-HF/TD-DFT) = -566.65352 Hartree
RMS Gradient Norm = 0.003789804 Hartree/Bohr
Dipole Moment = 1.7326372 Debye
Polarizability (?) = 185.11867 a.u.

Opt Tab Data Section:
Maximum force = 2.6e-05 Converged
RMS force = 5e-06 Converged
Maximum displacement = 0.000955 Converged
RMS displacement = 0.000128 Converged
Predicted energy change = -1.47819e-09 Hartree

PAI-S1-I$_{Ph}$175 (*s*-cis; A_{NNPh} = 175°)

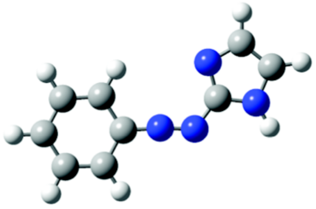

C
C,1,B1
C,1,B2,2,A1
C,1,B3,3,A2,2,D1,0
C,2,B4,1,A3,4,D2,0
C,2,B5,1,A4,4,D3,0
N,3,B6,1,A5,4,D4,0
N,7,B7,3,175.,1,D5,0
C,8,B8,7,A7,3,D6,0
N,9,B9,8,A8,7,D7,0
C,10,B10,9,A9,8,D8,0
C,11,B11,10,A10,9,D9,0
N,9,B12,8,A11,7,D10,0
H,13,B13,9,A12,8,D11,0
H,12,B14,11,A13,10,D12,0
H,1,B15,4,A14,5,D13,0
H,4,B16,1,A15,3,D14,0
H,5,B17,2,A16,1,D15,0
H,2,B18,1,A17,4,D16,0
H,6,B19,2,A18,1,D17,0
H,11,B20,10,A19,9,D18,0
Variables:
B1=2.81467412
B2=1.43433868
B3=1.3846201
B4=1.4047156
B5=1.38592917
B6=1.32109943
B7=1.23469699
B8=1.3603228
B9=1.33295087
B10=1.37306828
B11=1.37819651
B12=1.37572909
B13=1.00983349
B14=1.07959878
B15=1.08483908
B16=1.08730974
B17=1.08561376
B18=1.08735804
B19=1.08521567
B20=1.08171029
A1=59.86685283
A2=119.32635289
A3=59.87827939
A4=61.4827501
A5=119.43336368
A7=125.15777762
A8=127.95011582
A9=104.74162165
A10=111.3773832
A11=120.22593903
A12=125.79661147
A13=132.53394265
A14=121.96468856
A15=118.72581798
A16=120.3858527
A17=179.70310467
A18=121.40517757
A19=120.97336401
D1=0.00416927
D2=0.00024976
D3=180.00242631
D4=180.00096314
D5=179.84382081
D6=-179.84081028
D7=0.00157973
D8=180.00031196
D9=0.00012377
D10=-179.99768828
D11=-0.00089744
D12=180.00012801
D13=-180.00141516
D14=-180.00218856
D15=-180.00440241
D16=3.72859079
D17=180.00569416
D18=-179.99995976

Overview Tab Data Section:
Calculation Method = RB3LYP TD-FC
Formula = C9H8N4
Basis Set = 6-31+G(d)
Charge = 0
Spin = Singlet
Solvation = None
E(TD-HF/TD-DFT) = -566.64823 Hartree
RMS Gradient Norm = 0.000960212 Hartree/Bohr
Dipole Moment = 1.0750643 Debye
Polarizability (?) = 185.86833 a.u.

Opt Tab Data Section:
Maximum force = 1.4e-05 Converged
RMS force = 3e-06 Converged
Maximum displacement = 0.00053 Converged
RMS displacement = 0.000114 Converged
Predicted energy change = -1.434368e-09 Hartree

PAI-S1-I_{Ph}190 (*s-trans*; A_{NNPh} = 190°)

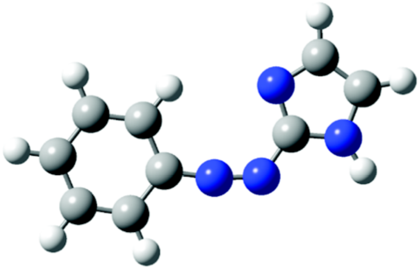

C
C,1,B1
C,1,B2,2,A1
C,1,B3,3,A2,2,D1,0
C,2,B4,1,A3,4,D2,0
C,2,B5,1,A4,4,D3,0
N,3,B6,1,A5,4,D4,0
N,7,B7,3,170.,1,D5,0
C,8,B8,7,A7,3,D6,0
N,9,B9,8,A8,7,D7,0
C,10,B10,9,A9,8,D8,0
C,11,B11,10,A10,9,D9,0
N,9,B12,8,A11,7,D10,0
H,13,B13,9,A12,8,D11,0
H,12,B14,11,A13,10,D12,0
H,1,B15,4,A14,5,D13,0
H,4,B16,1,A15,3,D14,0
H,5,B17,2,A16,1,D15,0
H,2,B18,1,A17,4,D16,0
H,6,B19,2,A18,1,D17,0
H,11,B20,10,A19,9,D18,0
Variables:
B1=2.8162535
B2=1.43452596
B3=1.38528557
B4=1.40486505
B5=1.38597608
B6=1.32690295
B7=1.23331692
B8=1.35932405
B9=1.33424061
B10=1.37289805
B11=1.3782615
B12=1.37614365
B13=1.00983122
B14=1.07959297
B15=1.08486408
B16=1.08740064
B17=1.08571168
B18=1.08728575
B19=1.08514067
B20=1.08169091
A1=59.70675579
A2=119.13030226
A3=59.84586354
A4=61.43404908
A5=120.32955654
A7=126.72233384
A8=127.82472583
A9=104.78276907
A10=111.35486712
A11=120.4398936
A12=125.74057925
A13=132.48199093
A14=122.26130962
A15=118.71613978
A16=120.35055208
A17=179.68934475
A18=121.40448087
A19=120.99179567
D1=0.
D2=0.
D3=180.
D4=180.
D5=-0.03281133
D6=0.04540783
D7=-0.00326735
D8=-179.99865213
D9=0.
D10=179.99788202
D11=0.00064378
D12=-180.
D13=-180.
D14=180.
D15=-179.99967825
D16=-0.04218888
D17=179.99949599
D18=180.

Overview Tab Data Section:
Calculation Method = RB3LYP TD-FC
Formula = C9H8N4
Basis Set = 6-31+G(d)
Charge = 0
Spin = Singlet
Solvation = None
E(TD-HF/TD-DFT) = -566.64965 Hartree
RMS Gradient Norm = 0.002034229 Hartree/Bohr
Dipole Moment = 1.1034832 Debye
Polarizability (?) = 178.70767 a.u.

Opt Tab Data Section:
Maximum force = 0.000191 Converged
RMS force = 5.6e-05 Converged
Maximum displacement = 0.001125 Converged
RMS displacement = 0.000239 Converged
Predicted energy change = -1.936866e-07 Hartree

PAI-S1-I_{Ph}205 (*s-trans*; A_{NNPh} = 205°)

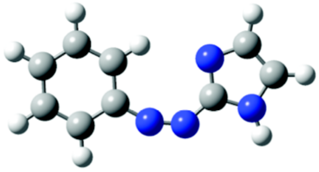

```
C
C,1,B1
C,1,B2,2,A1
C,1,B3,3,A2,2,D1,0
C,4,B4,1,A3,3,D2,0
C,2,B5,1,A4,4,D3,0
N,3,B6,1,A5,4,D4,0
N,7,B7,3,155.31431251,1,D5,0
C,8,B8,7,A7,3,D6,0
N,9,B9,8,A8,7,D7,0
C,10,B10,9,A9,8,D8,0
C,11,B11,10,A10,9,D9,0
N,12,B12,11,A11,10,D10,0
H,13,B13,12,A12,11,D11,0
H,12,B14,11,A13,10,D12,0
H,1,B15,4,A14,5,D13,0
H,4,B16,1,A15,3,D14,0
H,5,B17,4,A16,1,D15,0
H,2,B18,1,A17,4,D16,0
H,6,B19,2,A18,1,D17,0
H,11,B20,10,A19,9,D18,0
     Variables:
B1=2.81867249
B2=1.43101615
B3=1.38715473
B4=1.40492279
B5=1.38627562
B6=1.34147811
B7=1.23158579
B8=1.35064515
B9=1.33728461
B10=1.37140013
B11=1.37873457
B12=1.38132154
B13=1.00981153
B14=1.07958296
B15=1.0845001
B16=1.08748098
B17=1.08587443
B18=1.08721262
B19=1.08526494
B20=1.08171654
A1=59.58480574
A2=118.8846227
A3=121.59406185
A4=61.1838868
A5=121.45656263
A7=132.44708263
A8=128.45072644
A9=104.89230273
A10=111.40399801
A11=105.31963121
A12=127.45954855
A13=132.41791481
A14=122.22527091
A15=118.61133356
A16=120.3827892
A17=179.76343228
A18=121.29888289
A19=120.94157842
D1=-0.02130509
D2=0.03213183
D3=-180.01596965
D4=-179.98354524
D5=-0.76195877
D6=0.92629201
D7=-0.15276827
D8=180.01410056
D9=0.00664929
D10=-0.00744257
D11=-179.98887881
D12=-179.99734124
D13=180.02819806
D14=180.03857394
D15=-180.02349572
D16=-7.21077667
D17=179.99384976
D18=180.01312173
```

Overview Tab Data Section:
Calculation Method = RB3LYP TD-FC
Formula = C9H8N4
Basis Set = 6-31+G(d)
Charge = 0
Spin = Singlet
Solvation = None
E(TD-HF/TD-DFT) = -566.65401 Hartree
RMS Gradient Norm = 0.00163117 Hartree/Bohr
Dipole Moment = 1.6862427 Debye

Opt Tab Data Section:
Maximum force = 0.00037 Converged
RMS force = 9.6e-05 Converged
Maximum displacement = 0.001606 Converged
RMS displacement = 0.000393 Converged
Predicted energy change = -6.522358e-07 Hartree

PAI-S1-I_{Ph}220 (*s-trans*; A_{NNPh} = 220°)

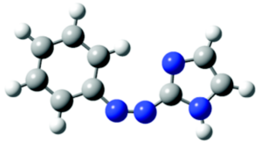

C
C,1,B1
C,1,B2,2,A1
C,1,B3,3,A2,2,D1,0
C,2,B4,1,A3,4,D2,0
C,2,B5,1,A4,4,D3,0
N,3,B6,1,A5,4,D4,0
N,7,B7,3,140.,1,D5,0
C,8,B8,7,A7,3,D6,0
N,9,B9,8,A8,7,D7,0
C,10,B10,9,A9,8,D8,0
C,11,B11,10,A10,9,D9,0
N,12,B12,11,A11,10,D10,0
H,13,B13,12,A12,11,D11,0
H,12,B14,11,A13,10,D12,0
H,1,B15,4,A14,5,D13,0
H,4,B16,1,A15,3,D14,0
H,5,B17,2,A16,1,D15,0
H,2,B18,1,A17,4,D16,0
H,6,B19,2,A18,1,D17,0
H,11,B20,10,A19,9,D18,0
Variables:
B1=2.79811826
B2=1.42166784
B3=1.38700365
B4=1.40275049
B5=1.3902911
B6=1.3626488
B7=1.23038804
B8=1.33802366
B9=1.3424784
B10=1.36924594
B11=1.37907658
B12=1.38082192
B13=1.00967876
B14=1.07954198
B15=1.08562138
B16=1.08713258
B17=1.08609581
B18=1.08751784
B19=1.08427242
B20=1.08181178
A1=59.93226575
A2=119.88557799
A3=60.0987484
A4=61.57515045
A5=117.74960445
A7=143.32475222
A8=128.99695093
A9=104.97990718
A10=111.55404071
A11=105.34912029
A12=127.43065234
A13=132.38078133
A14=121.14122689
A15=119.30144014
A16=120.3615056
A17=179.91430491
A18=121.63667134
A19=120.80833926
D1=0.00077346
D2=0.0002481
D3=180.00078144
D4=-179.99632818
D5=-179.98467746
D6=-0.09071306
D7=0.02637122
D8=179.99336393
D9=-0.00384275
D10=0.00589743
D11=-180.0018432
D12=180.00183487
D13=179.99925274
D14=-180.00001058
D15=-180.00148666
D16=4.20175192
D17=179.99848609
D18=-180.00370197

Overview Tab Data Section:
Calculation Method = RB3LYP TD-FC
Formula = C9H8N4
Basis Set = 6-31+G(d)
Charge = 0
Spin = Singlet
Solvation = None
E(TD-HF/TD-DFT) = -566.65433 Hartree
RMS Gradient Norm = 0.001556511 Hartree/Bohr
Dipole Moment = 2.2241108 Debye

Opt Tab Data Section:
Maximum force = 0.000164 Converged
RMS force = 3e-05 Converged
Maximum displacement = 0.000937 Converged
RMS displacement = 0.00015 Converged
Predicted energy change = -3.025275e-08 Hartree
